# Model updating method for detect and localize structural damage using generalized flexibility matrix and improved grey wolf optimizer algorithm (I-GWO)

**DOI:** 10.1038/s41598-025-09499-6

**Published:** 2025-07-07

**Authors:** Sina Sadraei, Majid Gholhaki, Omid Rezaifar

**Affiliations:** Faculty of Civil Engineering, Semnan University, Semnan, Iran

**Keywords:** Structural Health Monitoring, Damage Detection, Improved Grey Wolf Optimization Algorithm, Model updating Flexibility Matrix, Civil engineering, Mechanical engineering

## Abstract

Various civil engineering-based infrastructures have been strategically planned to implement the structural health monitoring (SHM) system, considering their significance. A key objective faced by this system is the automatic identification and damage detection at the appropriate moment. Employing optimization algorithms in structural model updating is one approach to achieve this objective. This study’s main goal is to evaluate the location and extent of damage by combining two dynamically evolving parameters: the structure’s frequency and the generalized flexibility matrix. It is determined that the suggested approach produces more accurate and effective outcomes than the previous modal flexibility techniques. This is achieved by applying various noises and extracting the damaged structure’s data using the Improved Grey Wolf Optimizer (I-GWO). The accuracy of this method in locating the 15-story shear frame, the 25-member two-dimensional truss bridge, and the 23-member two-dimensional frame, as well as in identifying all damages, is demonstrated by the fact that the error between the simulated and estimated results in an average of twenty runs and each damage scenario was less than 3 percent. The findings demonstrate that the technique can precisely pinpoint the position and extent of damage in various structures, hence increasing the effectiveness of damage identification. Furthermore, they show that when compared to grey wolf optimizer (GWO) and particle swarm optimizer (PSO), I-GWO can offer a dependable method for precisely detecting damage.

## Introduction

Upon prolonged utilization, it is imperative to assess the infrastructures with regards to their safety and sustainability. As time elapses, structural integrity may deteriorate leading to diminished performance, attributed to various factors like seismic activities, flooding, storms, among others. In extreme cases, this degradation could ultimately result in structural collapse.

With the introduction of cutting-edge technologies like information and signal processing, sensor networks and management systems, structural health monitoring, or SHM, has significantly improved safety, sustainability, infrastructure development, cost estimate, and exploitation management. Monitoring applications deliver essential data for constructing intelligent structures, including the necessary instruments for data acquisition prior to structure gets damaged more.

The evaluation of structural damage through conventional techniques of on-site examination or testing becomes impractical as a result of the growth in both the quantity and size of structures and their degradation. This is primarily due to the time-intensive and expensive nature of inspecting such structures, which is further compounded by the potential for human error. As a solution to this issue, novel approaches have been introduced for monitoring the remaining lifespan of intricate and substantial structures, which rely on alterations in the structural vibration characteristics. These approaches are commonly known as methodologies for identifying structural damage. The fundamental tenet is that the structure’s physical attributes have an impact on the modal data associated with it, including the frequency and flexibility matrix. Consequently, modifications to the physical qualities of the structure lead to modifications in its modal aspects. Therefore, the exact location and extent of the structural damage can be determined by comparing the modal features of the structure before and after damage^1^.

Damage detection methods usually involve two main components, static data and methods based on vibration analysis. Static data-based methods can measure displacement and strain, whereas dynamic methods which are chosen according to the particular dynamic properties involved include flexibility matrix approaches, frequency, modal strain energy, mode shape, and mode shape curvature.

It may be argued that static tests exhibit higher precision compared to dynamic tests; nevertheless, static methods demonstrate a lower sensitivity to variations in structural parameters. The utilization of static methods solely necessitates knowledge of stiffness properties, while dynamic methods involve stiffness, mass, and damping properties. When comparing static methods to dynamic methods, the accuracy of measured responses is higher; additionally, taking measurement errors into account improves the dependability of results from static methods compared to structural responses from modal testing.

Static techniques have been widely used by researchers in the field of structural health monitoring. Noteworthy original research on this subject has been conducted by Gudmundson^2^, Sanaeyi et al.^3^ and Wang et al.^4^. Bakhtiari-nejad et al.^5^ devised an approach for damage detection grounded on static test data. Through the resolution of non-linear simultaneous equations, their goal was to reduce the difference between the damaged structure’s and the undamaged one’s load vectors.

With the use of strain data on a plane frame and a plane truss, Esfandiari et al.^6^ presented a sensitivity-driven finite element model update technique for detecting alterations in a structure’s stiffness and mass parameters. In an analytical study of damage identification, Abdo^7^ proposed to identify changes in displacement curvatures based only on a static response for a two-span continuous beam and an overhanging beam.

Ni and Law proposed an approach that involves amalgamating and scrutinizing responses collected from diverse measurement setups. To directly analyze local damages, a parallel computing strategy and the Pattern Search method are used. Moreover, an assessment is conducted on the impact of employing a large Generating Matrix on the outcomes and precision. It is demonstrated that through the utilization of this approach, the identification of damage in a vast structure through short-term trials is feasible with the utilization of a limited number of sensors^8^. Seyedpoor^9^ introduced a technique for crack localization through an effective static database indicator. According to this study, finite element simulation was used to ascertain the static reactions of an Euler–Bernoulli beam.

Sanayei et al.^10^ outlined a strategy for updating the finite element model, taking into account the load scenarios and measurement points from nondestructive assessments. Using experimental data, the simultaneous calculation of mass and stiffness parameters was made possible by Monte Carlo simulation.

Dynamic techniques are an additional approach to damage detection. Lin^11^ noted that the higher modes of the system contribute significantly to the stiffness matrix compared to the lower modes. Thus, an accurate assessment of the stiffness matrix and its alterations is essential to detect damage by capturing all modes, particularly the higher ones. Furthermore, higher frequency modes are more difficult to capture during structural modal testing than lower frequency modes. A number of innovative methods for damage detection have been put forth in order to overcome this practical difficulty. These methods use the flexibility matrix to forecast changes in the stiffness matrix.

The flexibility matrix of a structure is the inverse of its global stiffness matrix and comes in two different types: static flexibility and dynamic flexibility. A unit force is applied to the structure to evaluate its static flexibility, whereas modal data analysis is used to estimate its dynamic flexibility. The lower modes of the structure are used to precisely compute the modal flexibility matrix, which is an approximation of the flexibility matrix. This study presents a novel method by using both categories at the same time.

In comparison to mode shapes and natural frequencies, a number of researchers have shown that the modal flexibility characteristic alone is more sensitive in identifying damages. In a theoretical investigation by Zhao et al.^12^, The usefulness of modal flexibility, natural frequencies, and mode shapes for structural health monitoring was compared. The findings indicated that modal flexibility outperformed the other parameters in damage localization. Pandey et al.^13^ described a damage detection system that used both numerical and experimental changes in the structure’s measured flexibility. Nevertheless, this method was imprecise in detecting situations in which the structure was damaged in several places. As a result, Yan et al.^14^ developed a damage indicator for a five-story steel frame and a truss based on strain flexibility. This unique approach was useful in cases where baseline data of the undamaged structure was unavailable and was helpful in identifying various damages within individual members.

Kim^15^ introduced a novel approach to assess damage in a slender beam subjected to axial force without causing destruction. This method relies on the utilization of dynamically obtained modal flexibility. Determining how to calculate the variations in modal flexibility brought on by the structural parameters—particularly the sensitivity of the modal flexibility—is a critical component of the many investigative theories pertaining to problems involving modal flexibility-based damage identification. In recent years, numerous techniques for assessing flexibility’s sensitivity have been proposed. Li et al.^16^ demonstrated a novel method that employs modifications in the generalized flexibility matrix to identify the location and degree of structural damage using a numerical example of a simply supported beam. This approach effectively mitigates the impact of omitting higher-order modes.

Another technique was suggested by Zhao et al.^17^, involving the addition of known masses to the structure, followed by the utilization of this "supplementary data" together with the original test data, enabling the detection of structural damages using the generalized flexibility perturbation method. Additionally, to ascertain the extent and location of structural damage, A closed-form equation for modal flexibility sensitivity based on the algebraic Eigen sensitivity technique was presented by Yan and Ren^18^.

This study introduces a metaheuristic-based damage identification approach using a Hybrid Quantum Genetic Algorithm (HQGA). By combining quantum computing principles with genetic algorithms, the method enhances global search capability for complex optimization problems. Applied to strain response data, the HQGA demonstrates high accuracy, robustness, and improved convergence efficiency.

Xu et al.^19^ introduced a metaheuristic-based damage identification approach using a Hybrid Quantum Genetic Algorithm (HQGA). By combining quantum computing principles with genetic algorithms, the method enhances global search capability for complex optimization problems. Applied to strain response data, the HQGA demonstrates high accuracy, robustness, and improved convergence efficiency. Ding et al.^20^ proposed a novel metaheuristic framework for structural damage identification based on a piecewise multi-objective function and Sparse Bayesian Learning (SBL). Their method combines rough global search and fine local refinement, enhanced by sparsity-driven strategies like elite clustering. Numerical and experimental results demonstrate high accuracy and robustness, even when limited modal data is available. Wan et al.^21^ proposed an iterative damage identification strategy using the Modified Jaya (M-Jaya) algorithm combined with Tikhonov regularization for force identification. The M-Jaya algorithm, a metaheuristic method, is enhanced with probabilistic clustering learning and nonlinear updating equations to improve convergence and solution quality. An improved L-curve method based on B-spline interpolation addresses the ill-posedness in force identification. Numerical and experimental studies demonstrate that the approach can simultaneously identify structural damages and unknown input excitation without force measurements, showing robustness against noise. Zhang et al.^22^ developed a novel output-only structural damage identification approach using a hybrid metaheuristic algorithm called QHEA, which combines Jaya, Differential Evolution (DE), and Q-learning. The method iteratively updates unknown structural parameters and input forces by minimizing the difference between measured and reconstructed acceleration responses. QHEA adaptively selects search strategies to balance exploration and exploitation, showing superior performance over other metaheuristics like MDE and I-Jaya. Numerical and experimental studies on truss and frame structures demonstrated high accuracy even under noise and modeling uncertainties. Despite requiring known excitation locations and sufficient sensors, the method offers robust and accurate damage detection without direct input measurements.

These developments motivate the present study, which integrates dynamic information through flexibility matrices and modal frequencies, combined with an enhanced metaheuristic optimization strategy, to improve the accuracy and robustness of damage identification in practical applications.

In recent years, dynamic-based damage detection methods have gained significant attention due to their higher sensitivity to local stiffness degradations compared to static approaches. Unlike static methods, which may overlook small damages or require large deformations, dynamic techniques leverage changes in natural frequencies, mode shapes, and derived parameters such as generalized flexibility matrices to provide more reliable and sensitive identification of structural damages. Several studies have demonstrated the effectiveness of dynamic-based approaches under noisy and uncertain measurement conditions.

Alkayem et al.^23^ proposed a dynamic structural damage identification method using a hybrid modal objective function combining strain energy, kinetic energy, and modal assurance criterion. This approach extracts detailed dynamic features of the structure, enhancing robustness against noise. To solve the inverse dynamic problem, they developed OL-UPSGBO by integrating oppositional learning with UPSO and GBO algorithms. Their method was validated on an ASCE benchmark frame under various damage cases. Results showed accurate and reliable dynamic damage detection even under noisy conditions.

Alkayem et al.^24^ developed a dynamic structural health monitoring (SHM) method using the Social Engineering Particle Swarm Optimization (SEPSO) algorithm, combining PSO and SEO strategies. SEPSO improves search efficiency and convergence for solving the dynamic inverse problem of structural damage detection. Applied to an ASCE frame under partial and noisy modal data, it showed strong accuracy and robustness. Results confirm SEPSO’s effectiveness for dynamic damage identification in complex engineering scenarios.

Ding et al.^25^ developed a Modified Artificial Bee Colony (ABC) algorithm for dynamic structural damage identification under temperature variations. A novel objective function based on frequency shifts, mode shape curvature, and a sparse penalty term enhanced sensitivity to dynamic changes. Numerical and experimental results confirmed that the Modified ABC accurately and robustly detected dynamic damages even under noise and environmental effects.

Zhang et al.^26^ developed an output-only dynamic damage identification method using strain response correlations and swarm intelligence algorithms. Tested on beam and grid structures under unknown excitations, the method accurately detected damages with limited sensors and noisy data, with MS-Jaya showing the best performance.

This method offers the advantage of mitigating the adverse effects of neglecting higher-order modes. Furthermore, it demonstrates the capability to yield accurate outcomes by employing single or multiple modes, while also addressing issues related to operational mode shape normalization through the utilization of scaling factors. The precise formulation of the sensitive equation in this method exhibits enhanced accuracy in comparison to Yan’s approach.

Considering the advantages and disadvantages of employing flexible methods, the focus shifts to the utilization of this approach for damage detection. This study explores a technique for identifying damage that integrates elements from the two aforementioned categories. To put it another way, the strategy depends on using both static and dynamic flexibilities at the same time. To put it briefly, a proposed method known as modified modal flexibility is introduced, which makes use of both changes in the modal flexibility matrix’s sensitivity and recorded static flexibility data.

It is demonstrated how to use flexibility and sensitivity data to update finite element models. In order to determine the position and extent of damage in structural elements, this method seeks to reduce the differences between the measured data and the analytical model. Damage assessment is predicated on linear stiffness reduction, in which stiffness reduction is proportionate to the drop in Young’s modulus and damping factors are absent from the equation of motion. The basic idea of this strategy is to modify the modal flexibility sensitivity matrix by using the measured frequency of the damaged structure. The proposed technique focuses on the direct utilization of the damaged structure’s measured frequency, altering the modal flexibility sensitivity equation and replacing the derivative of frequency. The flexibility method, characterized by the nature of its formula, exhibits a higher sensitivity to low-frequency modes, prompting the calculation of only the first one or two modes due to challenges in measuring all modes. Through adjustments and comparisons with existing methods, the proposed approach demonstrates effective performance in damage localization and severity assessment. The robustness of the updated results from this method is evident against incomplete, noisy data, and errors in measurement. Furthermore, even with increased noise levels, the proposed method maintains convergence and provides accurate estimations of structural damage. Furthermore, the suggested approach investigates how sensitive the results are to noise intensity, enabling the modification of noise levels to assess damage detection and result convergence.

The reason for the I-GWO algorithm’s selection is inherently tied to the drawbacks of alternative metaheuristic optimization algorithms in the face of inverse problems (such as inadequate speed and accuracy in complex solution landscapes).

One of the key improvements lies in its faster convergence rate, achieved through adaptive parameter tuning mechanisms. I-GWO balances exploration and exploitation more effectively across the optimization process. This allows the algorithm to search the solution space more thoroughly in early stages and refine the best solutions in later stages. An essential enhancement of I-GWO is its ability to escape local optima, a common limitation in the standard GWO. This is accomplished by introducing stochastic perturbations or modified encircling strategies that maintain population diversity. The improved mechanism increases the chances of reaching the global optimum, especially in complex and multimodal search spaces. Furthermore, I-GWO employs advanced leader selection or weighting strategies to better guide the search process. This results in improved stability and lower variance in the optimization results over multiple independent runs. The algorithm’s robustness to initial population distribution and parameter sensitivity is also notably increased. In applications such as structural damage detection, I-GWO achieves higher accuracy and better localization of damaged elements. It also reduces the rate of false positives and enhances detection reliability under measurement noise. Compared to GWO, the improved variant shows superior performance in both convergence behavior and solution quality. Benchmark tests on standard optimization functions confirm I-GWO’s effectiveness in handling complex landscapes. The algorithm adapts more efficiently to non-linear and high-dimensional problems. Additionally, I-GWO often requires fewer iterations to reach a satisfactory solution, reducing computational cost. It maintains a flexible framework that can be hybridized or further customized for specific engineering applications. These advantages make I-GWO a compelling choice for real-world optimization problems. Its simplicity, coupled with improved performance, ensures wide applicability and ease of implementation. Overall, I-GWO is a robust, accurate, and efficient alternative to the traditional GWO algorithm.

Finally, the methods discussed are supported by three numerical examples that tackle various issues that affect the idealized presumptions of theoretical approaches in real-world health assessment projects. To evaluate the speed and effectiveness of I-GWO in resolving the damage detection conundrum, a comparative study involving I-GWO, GWO, and one of the widely used optimization algorithms, PSO, is also carried out.

To clearly position the motivation and contributions of this study, the main research gaps and corresponding contributions are summarized as follows:

### Research gaps

• Existing flexibility-based damage detection methods show limited accuracy, particularly under noisy conditions and in complex structures.

• Traditional frequency-based approaches are inadequate for detecting minor or multiple damages using limited modal data.

• Static and dynamic flexibility methods individually face challenges, such as reduced sensitivity or vulnerability to measurement noise.

• Optimization algorithms like standard GWO often suffer from premature convergence and entrapment in local optima when applied to complex inverse problems.

• Generalized flexibility-based techniques have insufficient robustness against high levels of environmental and measurement noise.

### Contributions

• Proposing a hybrid model updating method that integrates generalized flexibility matrices with frequency data for improved damage identification.

• Developing an Improved Grey Wolf Optimizer (I-GWO) incorporating dimension learning-based hunting to enhance convergence and escape local minima.

• Designing a modified sensitivity-based objective function using generalized flexibility for accurate damage localization under noisy and incomplete data conditions.

• Validating the method through numerical studies on truss, frame, and shear frame structures across various damage scenarios and noise levels.

• Demonstrating that the proposed I-GWO method consistently outperforms GWO and PSO in accuracy and robustness, achieving less than 3% average error.

In this paper, an improved framework for structural damage identification is developed by combining the generalized flexibility matrix with an enhanced Grey Wolf Optimizer. The paper begins with an introduction that highlights the motivation, research gaps, and recent advances in optimization-based damage detection. Following the introduction, a concise review of related works in structural damage identification using metaheuristic algorithms is presented. The next section details the proposed methodology, including the formulation of a novel objective function that integrates flexibility variations and frequency shifts, and the improvements incorporated into the Grey Wolf Optimizer to enhance convergence and robustness. Subsequently, numerical studies are conducted on different structural models, such as truss, frame, and shear frame structures, to validate the effectiveness and noise resilience of the proposed method. A comprehensive discussion of the results is provided, analyzing the performance achievements, strengths, and existing limitations of the approach. Finally, the study concludes with a summary of key findings and suggestions for future research directions, emphasizing the potential of the developed framework for practical applications in structural health monitoring.

## Theory

### Gray wolf optimizer

The GWO algorithm^27^ offers a revolutionary global search strategy that is modelled after the social dominating hierarchy and hunting habits of grey wolves in the wild.

Grey wolves, known for their social nature, typically form groups consisting of five to twelve individuals for living and hunting purposes. Within this hierarchical structure, the alphas hold the highest position, leading the pack and making crucial decisions. Subordinate to the alphas are the betas, who support the alphas in decision-making and various pack activities. In situations where an alpha member is no longer able to fulfill their role, a beta wolf often emerges as a suitable replacement. The omegas, positioned at the lowest level of the hierarchy, play a significant role in maintaining pack integrity by obeying dominant wolves. Any pack member not classified as an alpha, beta, or omega is referred to as a subordinate or delta, displaying submission towards higher-ranking wolves while exerting dominance over the omegas.

Grey wolves also display an interesting social behavior related to their hunting behavior, which may be divided into three phases. The wolves first track, pursue, and get close to their intended victim. Then, in the second stage, they continue to hunt, surround, and threaten the victim until it stops trying to escape. At last, in the final phase, the wolves assault the victim. A mathematical model based on grey wolf social structure and hunting tactics was introduced by GWO. According to this concept, alpha (α), beta (β), and delta (δ) wolves stand in for the top three optimization process solutions, while omega (Ω) wolves stand in for the remainder solutions. Throughout the optimization process, the Ω wolves emulate the behaviors of the α, β, and δ wolves. To simulate the encircling behavior observed in grey wolves during hunting, a specific procedure has been put forth:

1 $${\text{D}}_{j} \& = \left| {{\text{C}}_{j} \cdot X_{p} \left( t \right) - X_{j} \left( t \right)} \right|$$

2 $$X_{j} \left( {t + 1} \right) = X_{p} \left( t \right) - {\text{A}}_{j} \cdot {\text{D}}_{j}$$

3 $${\text{A}}_{j} \& = 2{\text{a}}_{j} \cdot {\text{R}}_{1j} - {\text{a}}_{j}$$

4 $${\text{C}}_{j} \& = 2{\text{R}}_{2j}$$

In the analysis, ${A}_{j}$ and ${C}_{j}$ are denoted as coefficient vectors, ${X}_{p}(t)$ represents the prey’s position vector at iteration t, ${X}_{j}(t)$ signifies the j-th grey wolf at iteration t, ${R}_{1j}$ and ${R}_{2j}$ refer to random vectors within the range of [0,1] that are produced by the MATLAB® software, and ${a}_{j}$ is a vector with components that linearly decrease from 2 to 0 during iterative increments.

According to the mathematical model of grey wolf predation, alpha, beta, and delta wolves are better informed than omega wolves about the probable location of the prey. The top three solutions are thus maintained, and the optimal arrangement of the wolves surrounding the prey dictates the movement of the omega wolves.

In this context, the subsequent equations are put forth:

5 $${\text{D}}_{\alpha } = \left| {{\text{C}}_{1} X_{\alpha } - X_{j} \left( t \right)} \right|$$

6 $${\text{D}}_{\beta } = \left| {{\text{C}}_{2} X_{\beta } - X_{j} \left( t \right)} \right|$$

7 $${\text{D}}_{\delta } = \left| {{\text{C}}_{3} X_{\delta } - X_{j} \left( t \right)} \right|$$

8 $$X_{1} \left( {t + 1} \right) = X_{\alpha } \left( t \right) - {\text{A}}_{1} \cdot {\text{D}}_{\alpha }$$

9 $$X_{2} \left( {t + 1} \right) = X_{\beta } \left( t \right) - {\text{A}}_{2} \cdot {\text{D}}_{\beta }$$

10 $$X_{3} \left( {t + 1} \right) = X_{\delta } \left( t \right) - {\text{A}}_{3} \cdot {\text{D}}_{\delta }$$

11 $$X\left( {t + 1} \right) = \frac{1}{3}\left( {X_{1} \left( t \right) + X_{2} \left( t \right) + X_{3} \left( t \right)} \right)$$

Alpha, beta, and delta wolves’ spatial arrangements are taken into account while estimating the prey’s location within a hypersphere, and omega wolves’ positions are arbitrarily modified in relation to the prey. During the last stage of their hunting procedure, known as the exploitation phase, grey wolves leap on their victim as soon as it stops moving. Within the mathematical construct, the approach towards the prey is symbolized by the gradual reduction of the values of ${a}_{j}$ from 2 to 0 across successive iterations. As ${A}_{j}$ ranges between [-2 ${a}_{j}$, 2 ${a}_{j}$], the scope of fluctuation of ${A}_{j}$ diminishes, thereby enhancing the exploitation capacity of the algorithm. When the magnitude of ${A}_{j}$ is below 1.0, the wolves are compelled to launch an attack towards the prey. Conversely, if the magnitude of ${A}_{j}$ exceeds 1.0, the wolves are compelled to veer away from the prey through more erratic movements, in pursuit of a more suitable prey target. This systematic process fosters a comprehensive exploration strategy within the solution space, ultimately leading to the identification of the optimal solution with heightened probability.

#### Improved grey wolf optimizer algorithm

In the Grey Wolf Optimizer (GWO), the parameters α, β, and δ guide ω wolves towards regions within the search space that exhibit a higher likelihood of yielding the optimal solution. This particular behavior may result in the phenomenon of entrapment within locally optimal solutions. Additionally, a further consequence of this process is the diminished diversity within the population, which may lead the GWO to converge prematurely on local optima. To tackle these problems, an improved version of the Grey Wolf Optimizer (I-GWO) is presented in this section^28^. The enhancements encompass a novel inquiry methodology correlated with the selection and modification phase. the I-GWO encompasses three stages: initialization, locomotion, and selection and modification as delineated below.

Initializing phase: N wolves are dispersed at random across a specified range in the search space [${l}_{I}, {u}_{J}$] by Eq. (12).

12 $$X_{IJ} = l_{J} + rand_{J} \left[ {0,1} \right] \times \left( {u_{J} - l_{J} } \right), i \in \left[ {1, N} \right], j \in \left[ {1, D} \right]$$

The vector of real-valued values ${X}_{i}(t) = \{{x}_{i1}, {x}_{i2}, \dots ,{ x}_{iD}\}$ represents the location of the i-th wolf in the t-th iteration, where D is the problem’s dimensionality. The whole wolf population is arranged in a matrix Pop with N rows and D columns. The fitness function, $f({X}_{t}(t))$, is used to calculate the fitness measure of ${X}_{I}(t)$.

Movement phase: In addition to hunting in packs, grey wolves also engage in the intriguing social behavior of lone hunting^29^. The dimension learning-based hunting (DLH) search strategy is an extra locomotion technique that the I-GWO incorporates. Each wolf in DLH absorbs information from its peers to become a different candidate for the new role of ${X }_{i}\left(t\right)$ The next steps show how two different candidates are obtained using canonical GWO and DLH search techniques.

The typical method for GWO search: In GWO, the first three superior wolves from Pop are called α, β, and δ. The linearly decreased coefficient and, together with coefficients A and C, are then determined using Eqs. (3–4). Thereafter, the prey encirclement is ascertained by taking into account the positions of ${X}_{\alpha }$, ${X}_{\beta }$, and ${X}_{\delta }$ as delineated by Eqs. (5–10). Ultimately, the primary candidate for the updated position of wolf ${X}_{i}$ (t), designated as ${X}_{i-GWO}$(t + 1), is derived through Eq. (11).

The search approach known as "dimension learning-based hunting" (DLH): In the conventional Grey Wolf Optimizer (GWO), each wolf’s position is recalibrated utilizing the information from three leading wolves within the population. This methodology results in a gradual convergence of GWO, whereby the population experiences a premature loss of diversity, consequently leading to wolves becoming ensnared in local optima. Individual hunting among wolves, which is based on observations of their nearby counterparts, is incorporated into the suggested Dynamic Learning Hunting (DLH) search approach in order to overcome these drawbacks. ${X}_{i}$ (t).

Each element of the updated position of wolf ${X}_{i}$ (t) in the DLH search methodology is found using Eq. (15), in which this specific wolf integrates data from its different neighbors and a randomly selected wolf from the population.

Subsequently, in addition to ${X}_{i-GWO} (t+1)$, the DLH search methodology produces an alternative candidate for the updated position of wolf ${X}_{i} (t)$, referred to as ${X}_{i-DLH }(t+1)$. To accomplish this, an initial radius ${R}_{i}(t)$ is computed utilizing the Euclidean distance between the current location of ${X}_{i} (t)$ and the candidate location ${X}_{i-GWO}(t+1)$, as articulated in Eq. (13).

13 $$R_{i} \left( t \right) = X_{i} \left( t \right) - X_{i - GWO} \left( {t + 1} \right)$$

Subsequently, the neighborhood of ${X}_{i} (t),$ represented as ${N}_{i} (t)$, is formulated in accordance with Eq. (14) concerning the radius ${R}_{i} (t)$, where ${D}_{i}$ signifies the Euclidean distance between the points ${X}_{i} (t)$ and ${X}_{j} (t).$

14 $$N_{i} \left( t \right) = \left\{ {X_{j} \left( t \right)\left| {D_{i} \left( {X_{i} \left( t \right),X_{j} \left( t \right)} \right) \le R_{i} \left( t \right),X_{j} \left( t \right) \in Pop} \right.} \right\}$$

Upon the establishment of the neighborhood ${X}_{i} (t)$, the process of multi-neighbors learning is executed in accordance with Eq. (15), whereby the d-th dimension of ${X}_{i-DLH,d} (t+1)$ is derived through the utilization of the d-th dimension of a randomly selected neighbor ${X }_{\text{n},\text{d }}(t)$ from the set ${N}_{i} (t)$, as well as a randomly chosen wolf ${X}_{\text{r},\text{d }}(t)$ from the population Pop.

15 $$X_{i - DLH,d} \left( {t + 1} \right) = X_{i,d} \left( t \right) + {\text{rand}} \times \left( {X_{n,d} \left( t \right) - X_{r,d} \left( t \right)} \right)$$

Phase of selection and updating: During this phase, the optimal candidate is identified through the comparative analysis of the fitness values of two candidates, ${X}_{\text{i}-\text{GWO }} (t+1)$ and ${X}_{i-DLH } (t+1)$, as articulated by Eq. (16).

16 $$X_{i} \left( {t + 1} \right) = \left\{ {\begin{array}{*{20}c} {X_{i - GWO} \left( {t + 1} \right),} & {{\text{ if }}f\left( {X_{i - GWO} } \right) < f\left( {X_{i - DLH} } \right)} \\ {X_{i - DLH} \left( {t + 1} \right)} & {\text{ otherwise }} \\ \end{array} } \right.$$

**Fig. 1:**
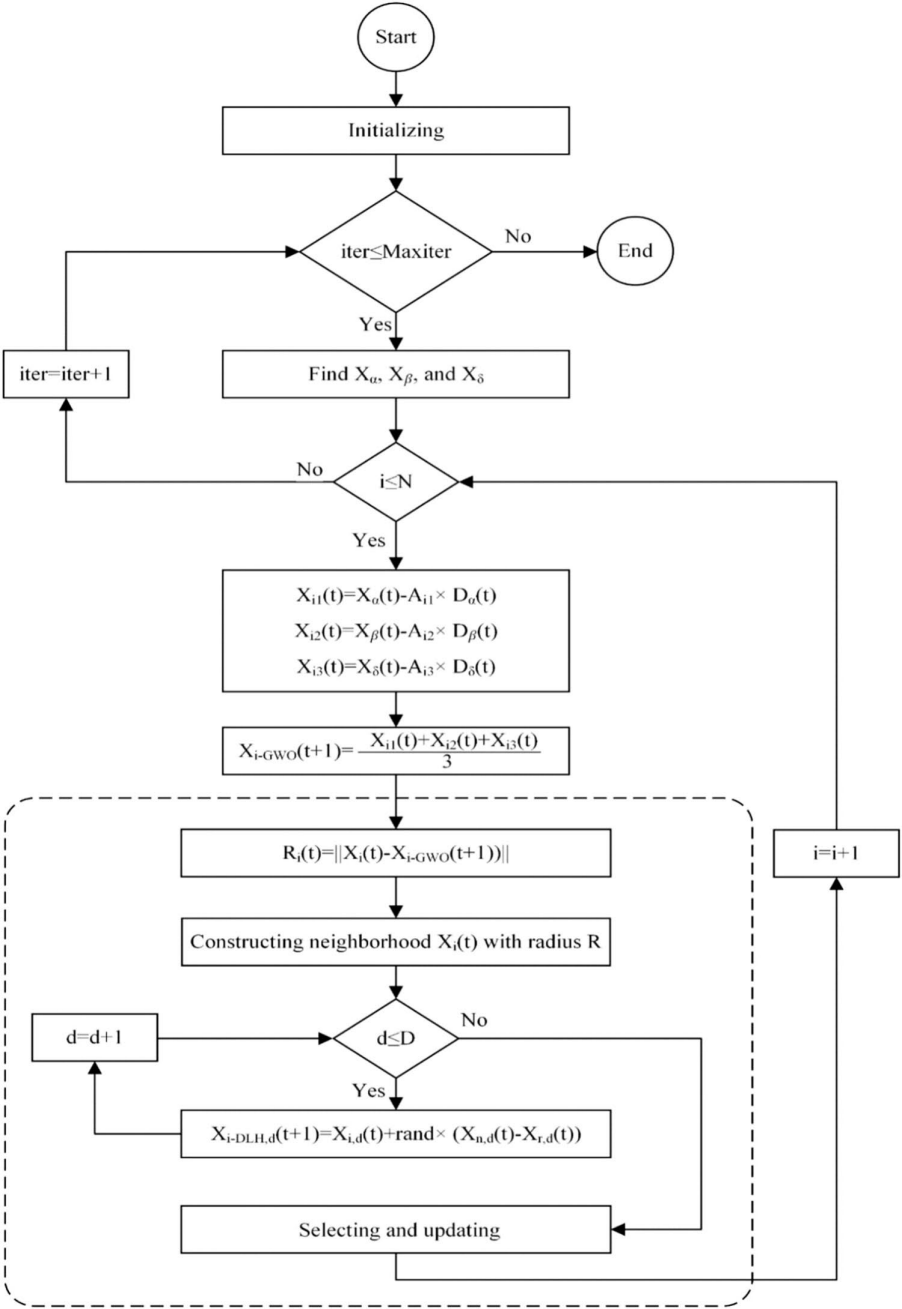
The flowchart of the I-GWO algorithm.

Subsequently, to revise the new position of ${X}_{t}$ (t + 1), should the fitness value of the chosen candidate be inferior to ${X}_{i} (t)$, then ${X}_{i} (t)$ is modified to reflect the selected candidate. Conversely, if the fitness value does not meet this criterion, ${X}_{i} (t)$ is retained in its current state within the population. When this process is completed for each individual, the iteration counter is then increased by one, allowing the search to continue until the predetermined number of iterations is reached. Figure 1 illustrates the overall workflow of the proposed I-GWO algorithm.

### Generalized modal flexibility

For a structural system that is linear and undamped, the equation governing its motion is expressed as:

17 $$\left[ M \right]\left\{ {\ddot{u}\left( t \right)} \right\} + \left[ K \right]\left\{ {u\left( t \right)} \right\} = \left\{ {P\left( t \right)} \right\}$$

The intact structure is represented by mass matrix [M] and the global stiffness matrix [K]. Based on the dynamic of structural sources, the steady-state harmonic response of Eq. (17) to the harmonic stimulation is as follows:

18 $$u = \left( { - \omega^{2} \left[ M \right] + \left[ K \right]} \right)^{ - 1} P = G\left( \omega \right)P$$

As a dynamic extension of the static flexibility matrix, the matrix G(ω) is known as the dynamic flexibility matrix, represented as $G\left(0\right)={k}^{-1}$ (ω, excitation frequency, is equal to zero)^30^. Upon multiplication of both sides by the eigenvector and its transpose, Eq. (18) produces:

19 $$\varphi^{{\text{T}}} G^{ - 1} \varphi = \varphi^{{\text{T}}} \left[ K \right]\varphi$$

For a structure with multiple degrees of freedom, when the mode shape vector $\{{\varphi }_{r}\}$ is normalized such that $\left\{{\varphi }_{r}\right\}\left[M\right]\left\{{\varphi }_{r}\right\}=1$, the flexibility matrix [*F*] can be represented for the initial low-frequency modes (*N*) as:

20 $$\left[ K \right] - 1 = \left[ F \right] = \sum\limits_{r = 1}^{N} {\frac{1}{{\lambda_{r} }}} \left\{ {\varphi_{r} } \right\}\left\{ {\varphi_{r} } \right\}^{T} = \sum\limits_{r = 1}^{N} {\left[ F \right]_{r} }$$

Equation (20) illustrates how the modal contribution to the flexibility matrix diminishes as frequency increases. Conversely, the flexibility tends to swiftly approach a satisfactory estimation with a limited number of low-frequency modes^31^. The determination of the flexibility matrix can be achieved through two approaches: utilizing either dynamic or static data. The precision of measuring static tests surpasses that of dynamic tests; nevertheless, static methods exhibit a minimal sensitivity to alterations in structural parameters. Specifically, dynamic approaches need the inclusion of mass, stiffness, and damping qualities, whereas static approaches only need the stiffness parameters.

The accuracy of responses obtained from static methods surpasses that of dynamic methods; furthermore, static results exhibit higher reliability than modal testing results when accounting for measurement errors. This study presents an approach to establishing the flexibility matrix using static data.

the generalized flexibility matrix within the framework of the following equation^15^:

21 $$f_{d}^{g} \left( \alpha \right) = F_{d} \left( {MF_{d} } \right)^{l} = {\Phi }_{d} {\Lambda }_{d}^{ - 1} {\Phi }_{d}^{T} \left( {M{\Phi }_{d} {\Lambda }_{d}^{ - 1} {\Phi }_{d}^{T} } \right)^{l} = {\Phi }_{d} {\Lambda }_{d}^{ - 1 - l} {\Phi }_{d }^{T} l = 0,1,2, \ldots$$

In this equation, [M] represents the structure’s total mass matrix, $\Phi_{d}$ is the mass-normalized modal shape matrix, and ${\Lambda }_{d}$ is the diagonal matrix that contains the structure’s squared frequency. It is crucial to emphasize that the first-order generalized flexibility matrix, in which the parameter *l* has a value of one, was used to establish the objective function in this investigation.

### Damage detection using the suggested approach

As a function of the modulus of elasticity, the stiffness of the damaged element decreases when the structure is damaged. Therefore, through the manipulation of Eq. (22) to decrease the modulus of elasticity in the components, the model accurately replicates the damage incurred by the structure.

22 $$E_{e}^{d} = \left( {1 - \alpha_{e} } \right)E_{e}$$

In the scenario where ${E}_{d}$ represents the modulus of elasticity pertaining to the damaged element, ${\alpha }_{e}$ signifies how much the element has been damaged, falling within the numerical range of zero to one. Here, a value of zero signifies the absence of any damage, while a value of one denotes complete damage amounting to 100% of the element. Additionally, ${E}_{e}$ stands for the modulus of elasticity associated with the component in its original, undamaged state.

23 $$\left[ {K - \omega_{i}^{2} M} \right]\left[ {\phi_{i} } \right] = 0,i = 1,2, \ldots ,n$$

Equation (23), for n of DOFs, yields the mode shapes and the square of the structure’s natural frequencies, respectively:

24 $$\left[ \omega \right] = \left[ {\begin{array}{*{20}c} {\omega_{11}^{2} } & \cdots & 0 \\ \vdots & \ddots & \vdots \\ 0 & \cdots & {\omega_{nn}^{2} } \\ \end{array} } \right]$$

25 $$\left[ \phi \right] = \left[ {\begin{array}{*{20}c} {\phi_{11} } & \cdots & {\phi_{1n} } \\ \vdots & \ddots & \vdots \\ {\phi_{n1} } & \cdots & {\phi_{nn} } \\ \end{array} } \right]$$

Regardless of the accuracy and computing difficulties, the approaches for updating models that account for the differences in flexibilities are limited to situations where accurate flexibility data of the damaged structures is available. Researchers usually incorporate a low degree of random errors (up to 8% of random errors) to assess how robust such model updating techniques are to measurement errors.

External interferences such as environmental noises from ambient loads or unstable sensor positions may influence the derived flexibilities, thus, encountering a higher degree of measurement inaccuracy should not be unexpected.

Measurement of natural frequencies can be conducted with precision through the utilization of advanced accelerometers. This observation has prompted certain scholars to posit the notion of noiseless natural frequencies when undertaking model refinement. The formation of a sensitivity matrix for the assessment of stiffness characteristics is accomplished by incorporating the ascertained natural frequencies. Nevertheless, the sensitivity equations are notably responsive to and impacted by any unforeseen inaccuracies in the natural frequencies.

In the event of noise, the impact within this particular domain is incorporated into the compromised structure through the subsequent mathematical expression:

26 $$\left[ {\omega_{P} } \right] = \left[ \omega \right] \times \left( {1 + N \times rand} \right)$$

where ${\omega }_{P}$ denotes the resultant output, while the parameter *N* signifies the level of noise.

As previously stated, the generalized flexibility matrix can be determined by utilizing the following equation:

27 $$f_{d}^{g} \left( \alpha \right) = F_{d} \left( {MF_{d} } \right)^{l} = {\Phi }_{d} {\Lambda }_{d}^{ - 1} {\Phi }_{d}^{T} \left( {M{\Phi }_{d} {\Lambda }_{d}^{ - 1} {\Phi }_{d}^{T} } \right)^{l} = {\Phi }_{d} {\Lambda }_{d}^{ - 1 - l} {\Phi }_{d }^{T} l = 0,1,2, \ldots$$

Therefore, through the utilization of Eqs. (24) and (25), it is possible to express the flexibility matrix in the following manner:

28 $$\left[ {F]_{n \times n} = } \right[\phi ]_{n \times nm} \left[ {\omega ]_{nm \times nm}^{ - 1} } \right[\phi ]_{n \times nm}^{T}$$

in which, *nm* is the number of the modes used. ${GFM}_{m}$ and ${GFM}_{d}$ are the generalized flexibility matrix for the damaged structure and for the model structure, respectively and can be determined by utilizing the following equation:

29 $$\,\,\,\begin{array}{*{20}c} {GFM_{d} = \left[ {\begin{array}{*{20}c} {a_{11} } & \cdots & {a_{1n} } \\ \vdots & \ddots & \vdots \\ {a_{n1} } & \cdots & {a_{nn} } \\ \end{array} } \right]\Lambda_{d} = \left[ {\begin{array}{*{20}c} {g_{11} } & \cdots & 0 \\ \vdots & \ddots & \vdots \\ 0 & \cdots & {g_{nn} } \\ \end{array} } \right]} \\ {GFM_{m} = \left[ {\begin{array}{*{20}c} {b_{11} } & \cdots & {b_{1n} } \\ \vdots & \ddots & \vdots \\ {b_{n1} } & \cdots & {b_{nn} } \\ \end{array} } \right]\Lambda_{m} = \left[ {\begin{array}{*{20}c} {h_{11} } & \cdots & 0 \\ \vdots & \ddots & \vdots \\ 0 & \cdots & {h_{nn} } \\ \end{array} } \right]} \\ \end{array}$$

${\Lambda }_{m}$ and ${\Lambda }_{d}$ respectively, the diagonal matrix contains the frequency square for the damaged structure and for the model structure. ${f}_{1}$ and ${f}_{2}$ is obtained as follows:

30 $$\begin{array}{*{20}c} {c_{n} = \max \left( {a_{nn} ,b_{nn} } \right)} \\ {d_{n} = \min \left( {a_{nn} ,b_{nn} } \right)} \\ {l_{n} = \frac{{d_{n} }}{{c_{n} }}} \\ \end{array} \Rightarrow f_{1} = \frac{{\mathop \sum \nolimits_{i = 1}^{n} {l_{i} } }}{n}$$

31 $$\begin{array}{*{20}c} {j_{n} = \max \left( {g_{nn} ,h_{nn} } \right)} \\ {k_{n} = \min \left( {g_{nn} ,h_{nn} } \right)} \\ {r_{n} = \frac{{k_{n} }}{{j_{n} }}} \\ \end{array} \Rightarrow f_{2} = \frac{{\sum\nolimits_{i = 1}^{n} {r_{i} } }}{n}$$

In the proposed damage identification framework, the objective function is designed to simultaneously consider the flexibility variation and the dynamic characteristics of the structure. Specifically, the objective function is defined as the multiplication of two components, ${f}_{1}$ and ${f}_{2}$.

The first term, ${f}_{1}$, represents the discrepancy between the generalized flexibility matrices of the measured (damaged) structure and the predicted (model) structure. Since the generalized flexibility matrix is a function of the structural stiffness, even minor damage affecting local stiffness can lead to noticeable changes in flexibility values. Thus, ${f}_{1}$ captures the global stiffness degradation information resulting from structural damages.

The second term, ${f}_{2}$, quantifies the error between the natural frequencies of the measured and model structures. Natural frequencies are sensitive to changes in mass and stiffness distribution; in the context of this work, they primarily reflect stiffness alterations caused by damage. Therefore, ${f}_{2}$ supplements the flexibility-based information with additional dynamic response features, enhancing the robustness of the damage detection process.

The multiplication of ${f}_{1}$ and ${f}_{2}$ ensures that both flexibility deviations and frequency shifts are simultaneously emphasized. Rather than treating the flexibility matrix and frequency information independently, the product formulation couples them in a way that penalizes inconsistencies across both domains. This design intensifies the sensitivity of the objective function to structural damages, particularly in noisy environments where relying on a single feature may be insufficient.

Consequently, the objective function facilitates a more reliable differentiation between intact and damaged structures. It leads the optimization process toward solutions that minimize both the flexibility and frequency mismatches, thereby achieving more accurate localization and quantification of structural damages, even under high noise levels and incomplete measurement conditions.

32 $$F = \left| {1 - \left( {f_{1} \times f_{2} } \right)} \right|$$

**Fig. 2:**
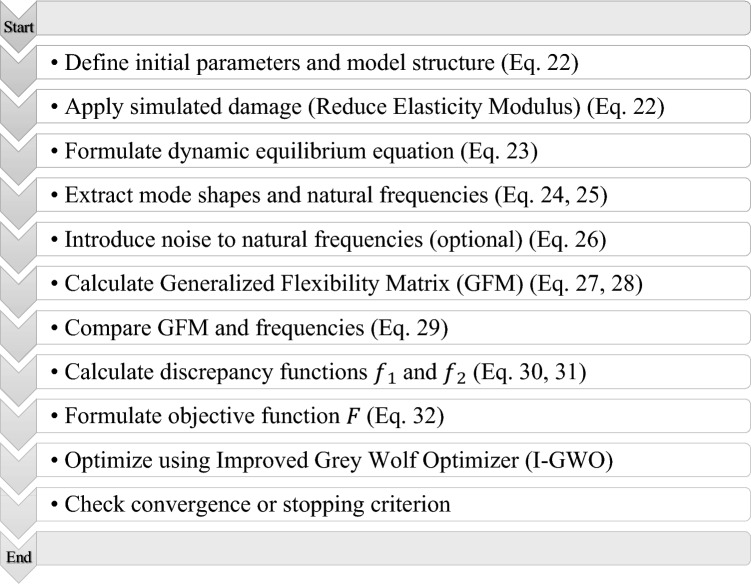
Overall damage detection method.

Hence, the objective function is articulated in the subsequent manner:

The I-GWO algorithm ultimately solves Eq. (32) to identify the optimal solution to the problem, which is given as the damage detection findings. The overall procedure of the structural damage detection approach is depicted in Fig. 2.

## Numerical results

In this section, the suitability of the methodology put forth is illustrated through an examination of two-dimensional steel shear frame, two-dimensional steel truss and two-dimensional steel moment frame structures subjected to diverse patterns of damage. Furthermore, the evaluation of the effectiveness of the objective function and the accuracy of the I-GWO algorithm is carried out by utilizing the minimum number of modes, introducing noise, and considering various scenarios. It is imperative to note that all analyses have been conducted within the computational environment of MATLAB software.

### Two-dimensional truss bridge

**Fig. 3:**
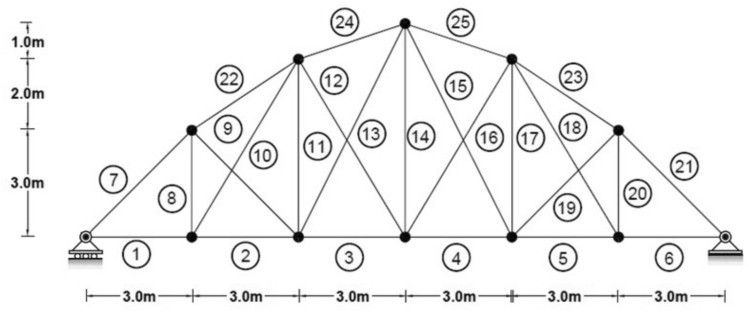
Two-Dimensional Truss.

Figure 3 shows the 25 elements that make up the finite element model for the 2-D truss element. Table 1 lists the material attributes that correlate to the damage scenarios in Table 2. Furthermore, the parameters of the optimization algorithm are detailed as follows: the maximum number of iterations is set to 900, the number of the population of wolves is 120, with an upper bound of 1 and a lower bound of 0. The outcomes of the damage identification process for the two-dimensional truss dataset under conditions of 0% noise, 2% noise and 4% noise are illustrated for the respective first, second, and third scenarios.

**Table 1:** Cross-sectional area a density of truss elements.

Element number	sectional area (m^2^)	Modulus of elasticity	Weight per unit length (kg/L)
1,2,3,4,5,6	0.01	11e + 2	3500
7,21,22,23,24,25	0.01	11e + 2	78.5
8,11,14,17,20	0.005	11e + 2	39.25
9,10,12,13,15,16,18,19	0.008	11e + 2	62.8

**Table 2:** Different damage scenarios for the truss.

The number of modes	Damage scenario	Damaged element	Damage rate
2	Scenario 1	23	25%
	Scenario 2	10	5%
		18	15%
	Scenario 3	2	10%
		12	20%
		17	15%
4	Scenario 1	23	25%
	Scenario 2	10	5%
		18	15%
	Scenario 3	2	10%
		12	20%
		17	15%

**Fig. 4:**
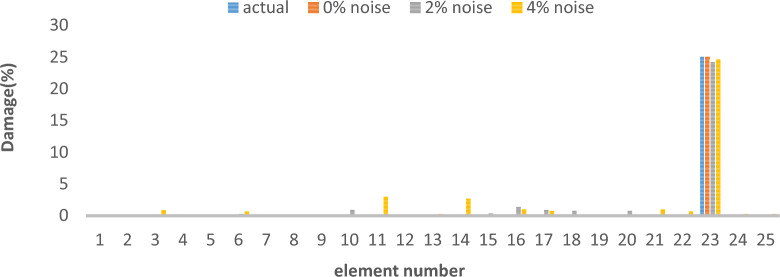
Damage identification results of 25-member flat truss in the first damage scenario using the information of the first 2 modes.

**Fig. 5:**
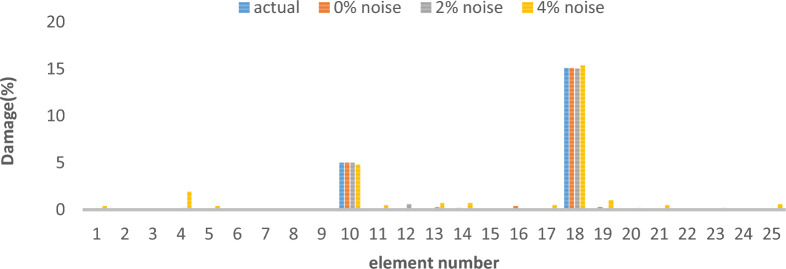
Damage identification results of 25-member flat truss in the second damage scenario using the information of the first 2 modes.

**Fig. 6:**
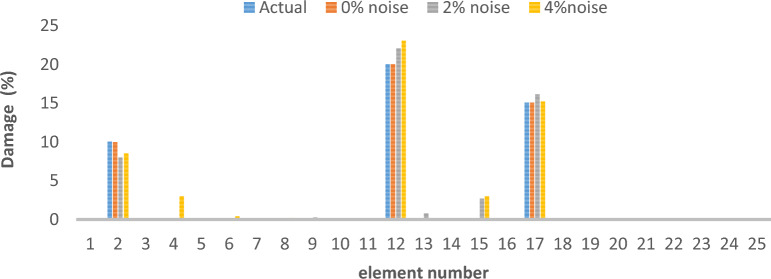
Damage identification results of 25-member flat truss in the third damage scenario using the information of the first 2 modes.

**Fig. 7:**
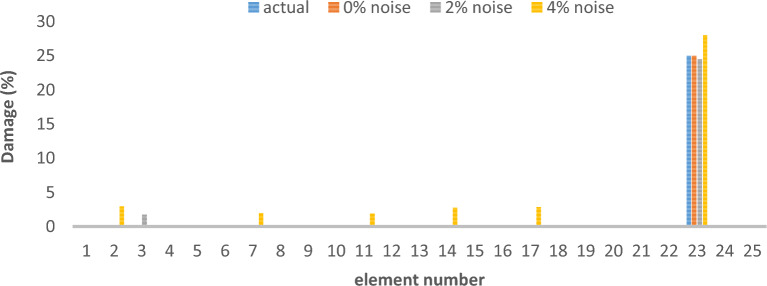
Damage identification results of 25-member flat truss in the first damage scenario using the information of the first 4 modes.

**Fig. 8:**
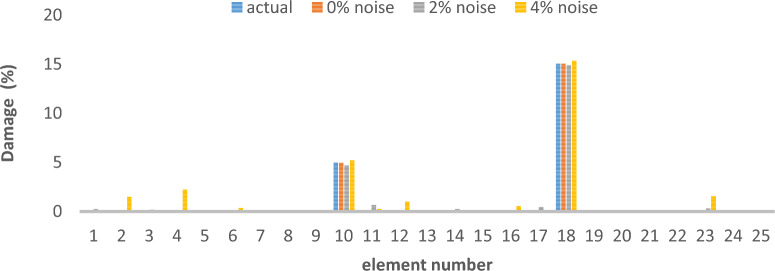
Damage identification results of 25-member flat truss in the second damage scenario using the information of the first 4 modes.

**Fig. 9:**
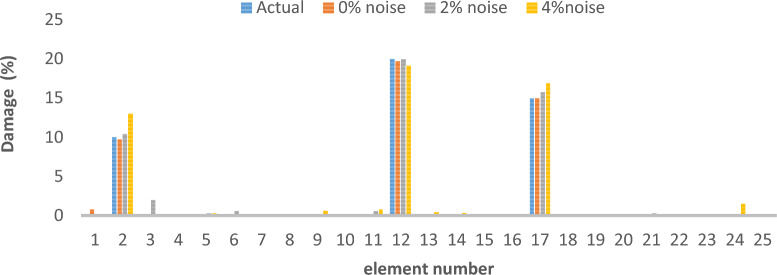
Damage identification results of 25-member flat truss in the third damage scenario using the information of the first 4 modes.

As can be seen in Figs. 4, 5, 6, 7, 8, 9, with only 1500 iteration, even considering the effect of noise, very high accuracy results can be achieved.

By comparing the results, we can come to the conclusion that in the absence of noise, even using the information of the first 2 modes, we can achieve results with the same accuracy as when we use the information of the 4 modes. For example, by comparing Figs. 6 and 9, it is quite evident that the results of identifying the 4 damages considered in the third scenario using a higher number of modes (the first 4 modes) have a similar accuracy compared to when information of the first 2 modes is used.

Another noteworthy point is that, in the presence of negative effects of noise, using higher mode number information increases the negative effects of noise and may lead to more error in damage detection. For instance, it is evident from comparing Figs. 4 and 7 that damage detection using the first two modes is more accurate than damage detection using the first four modes when there is 4% noise.

**Fig. 10:**
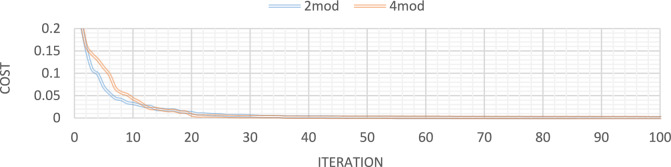
Convergence curve of the first damage scenario in the absence of noise for first 2 and 4 modes.

Figures 10 through 13 also display the convergence curve of the added goal function. These figures make it evident that more time is required to identify damage as the number of damaged structural elements increases, particularly in the presence of noise.

**Fig. 11:**
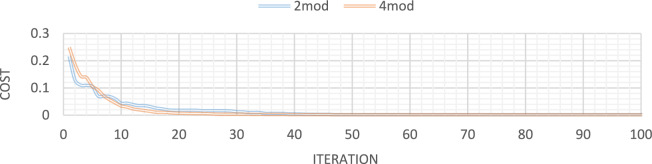
Convergence curve of the first damage scenario in the presence of 4% noise for first 2 and 4 modes.

**Fig. 12:**
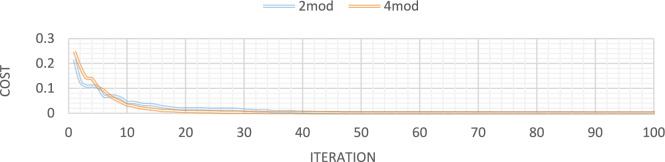
Convergence curve of the third damage scenario in the absence of noise for first 2 and 4 modes.

**Fig. 13:**
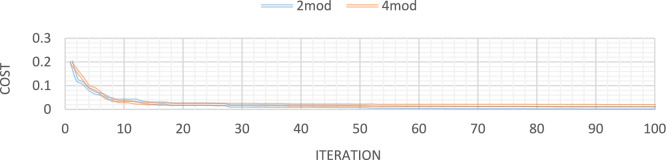
Convergence curve of the third damage scenario in the presence of 4% noise for first 2 and 4 modes.

To evaluate the robustness of the proposed method for the truss structure under noisy conditions, additional simulations were performed by introducing noise with intensities of 10%, 15%, and 20% into the strain measurements. The results show that the method maintains high identification accuracy for noise levels up to 10%, with only a slight increase in damage quantification errors. Even at 15% noise, although a moderate increase in error is observed, the method can still accurately detect and localize the damaged elements without significant false identifications. These findings confirm that the proposed approach exhibits strong noise resistance when applied to truss structures, maintaining reliable damage detection performance even under high levels of measurement uncertainty.

### Two-dimensional frame

**Fig. 14:**
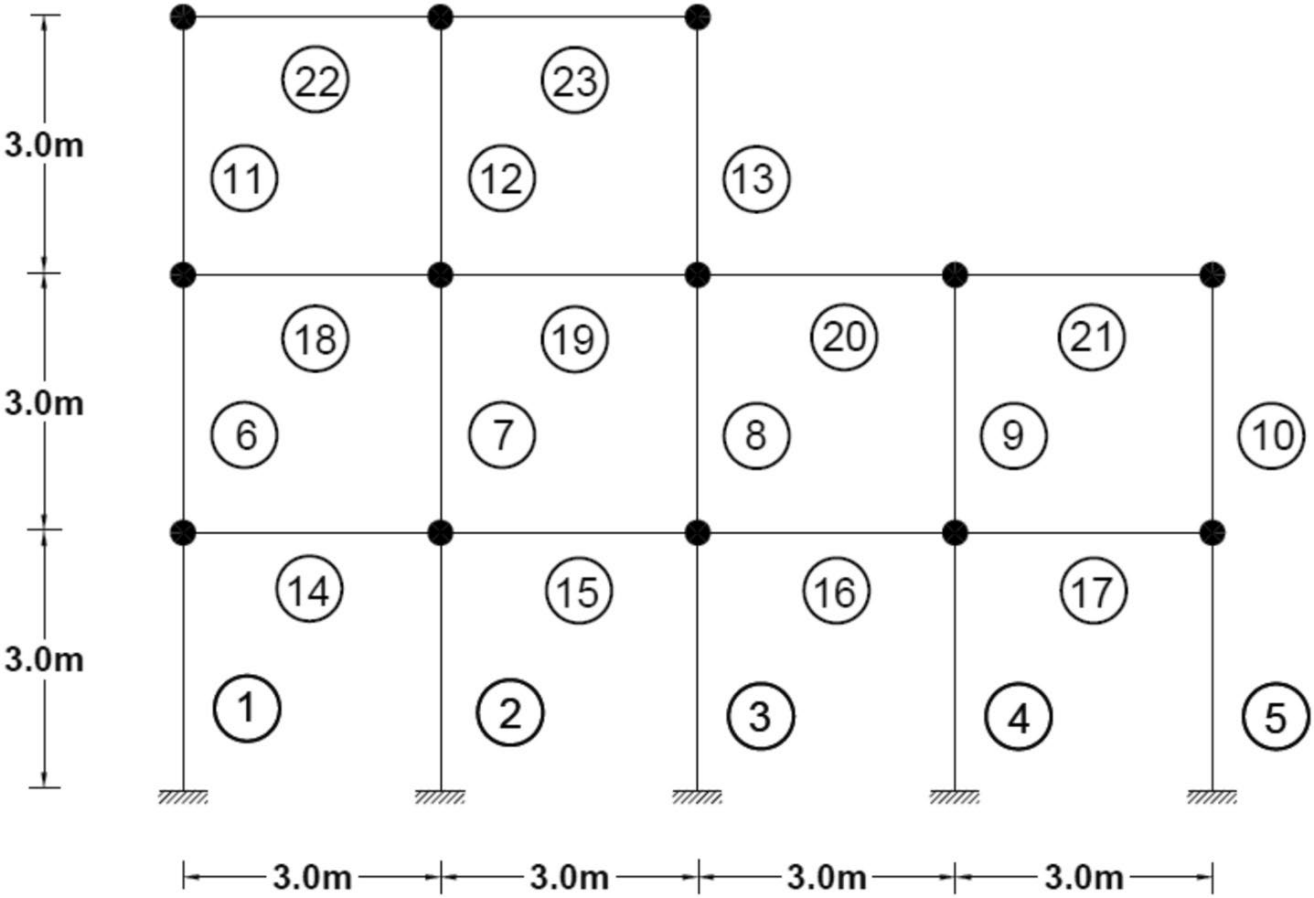
Two-Dimensional Frame.

The structural system in question is represented by a two-dimensional frame model, comprising a total of 23 elements and 18 nodes, each possessing three degrees of freedom, as depicted in Fig. 14. Table 3 presents the material properties, followed by various damage scenarios detailed in Table 4. The optimization algorithm’s parameters are as follows: The wolf population size is 120, with an upper bound of 1 and a lower bound of 0, and the maximum number of iterations permitted is 950.

**Table 3:** Cross-sectional area and density of frame.

Element	sectional area (m^2^)	Moment of inertia	Weight per unit length (kg/L)	Modulus of elasticity
column	0.016	0.00035	125.6	11e + 2
beam	0.0162	0.000385	1300	11e + 2

**Table 4:** Different damage scenarios for the frame.

The number of modes	Damage scenario	Damaged element	Damage rate
1	Scenario 1	4	5%
		18	10%
	Scenario 2	2	15%
		9	10%
		19	20%
	Scenario 3	1	15%
		8	20%
		16	25%
		22	10%
3	Scenario 1	4	5%
		18	10%
	Scenario 2	2	15%
		9	10%
		19	20%
	Scenario 3	1	15%
		8	20%
		16	25%
		22	10%

The results of damage detection under conditions of 0% noise and 5% noise are presented.

**Fig. 15:**
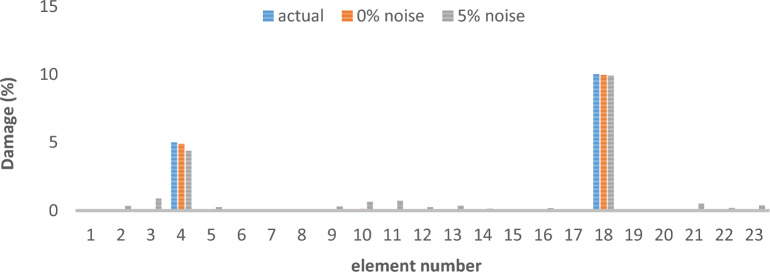
Damage identification results of 23-member frame in the first damage scenario using the information of the first mode.

**Fig. 16:**
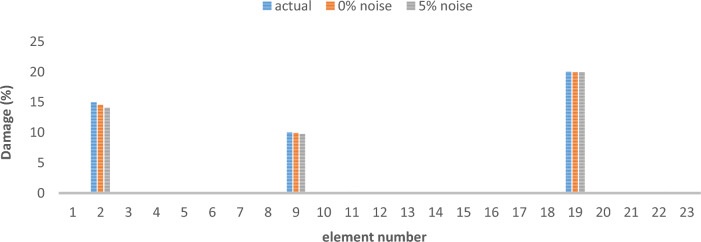
Damage identification results of 23-member frame in the second damage scenario using the information of the first mode.

**Fig. 17:**
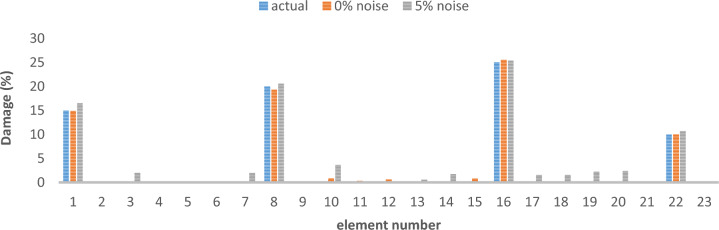
Damage identification results of 23-member frame in the third damage scenario using the information of the first mode.

**Fig. 18:**
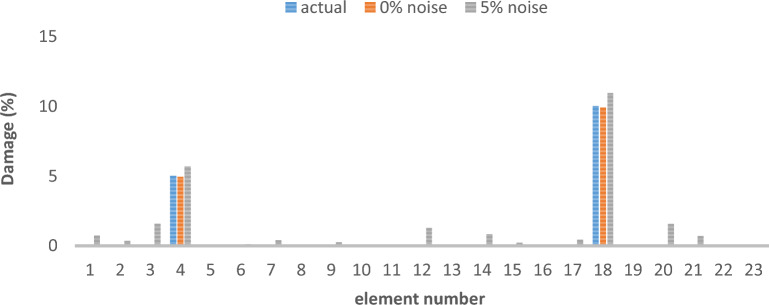
Damage identification results of 23-member frame in the first damage scenario using the information of the first 3 modes.

**Fig. 19:**
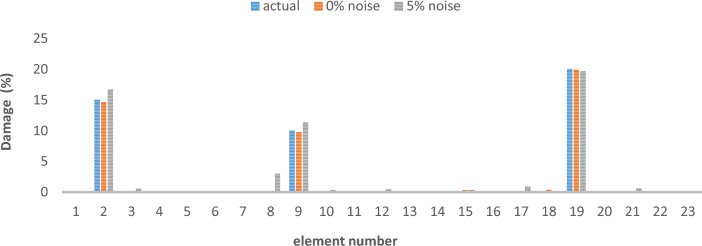
Damage identification results of 23-member frame in the second damage scenario using the information of the first 3 modes.

**Fig. 20:**
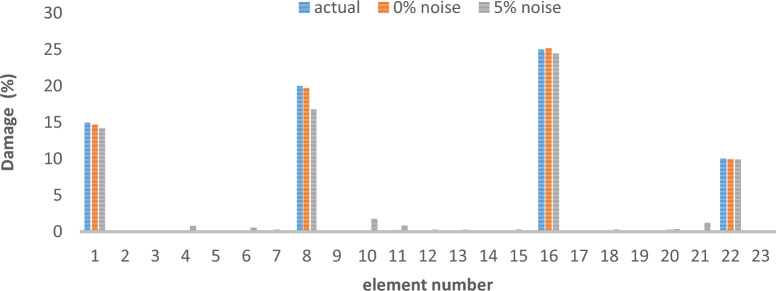
Damage identification results of 23-member frame in the third damage scenario using the information of the first 3 modes.

As can be seen in Figs. 15, 16, 17, 18, 19, 20, with only 1500 repetitions, even considering the effect of noise, very high accuracy results can be achieved. It should be noted that according to the obtained results, the proposed objective function has a better performance in the modeled frame structure than the 25-member flat truss.

According to the results obtained in case of absence of noise, by using the information of the first mode, it is possible to reach the results with the same accuracy as when we use the information of the first 3 modes. This is clearly evident by comparing Figs. 15 and 20.

The results of identifying the 4 damages considered in the third scenario, considering the number of modes (the first 3 modes), have a similar accuracy compared to when the information of the first mode is used.

Another noteworthy point is that, in the presence of negative effects of noise, using higher mode number information increases the negative effects of noise and may lead to more error in damage detection. For example, by comparing Figs. 16 and 19, it is quite clear that in the condition of 5% damage detection with the first mode is more accurate than the damage detection with the first 3 modes.

**Fig. 21:**
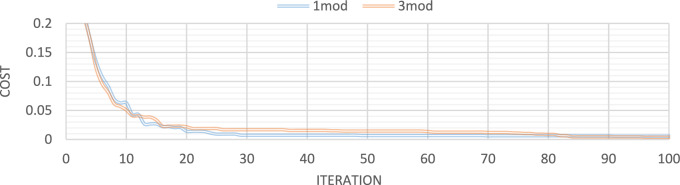
Convergence curve of the first damage scenario in the absence of noise for first 1 and 3 modes.

**Fig. 22:**
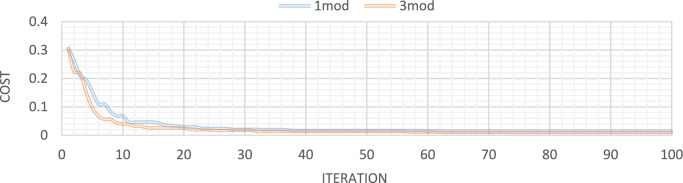
Convergence curve of the first damage scenario in the presence of 5% noise for first 1 and 3 modes.

**Fig. 23:**
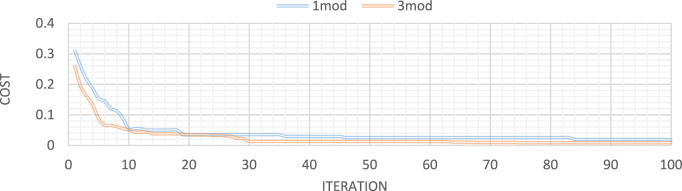
Convergence curve of the third damage scenario in the absence of noise for first 1 and 3 modes.

**Fig. 24:**
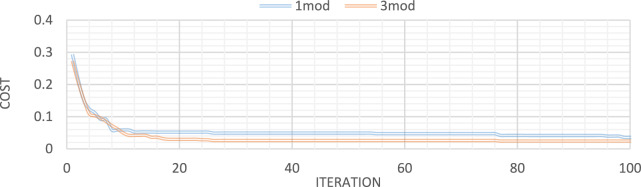
Convergence curve of the third damage scenario in the presence of 5% noise for first 1 and 3 modes.

Figures 21, 22, 23, 24 show display the convergence curve for the inserted goal function. These figures make it evident that as the number of damaged structural elements increases, it takes longer to find the damage, particularly when there is noise present.

To assess the noise robustness of the proposed method when applied to the frame structure, additional numerical studies were conducted by adding Gaussian white noise at 10%, 15%, and 20% intensity levels to the strain response data. The results indicate that for noise levels up to 15%, the proposed approach successfully detects and quantifies the damages with minimal degradation in accuracy. When the noise intensity increases to 20%, a moderate reduction in identification precision is observed; however, the method continues to accurately locate the damaged regions without generating false positives. These outcomes demonstrate that the proposed framework remains reliable and robust even under considerable measurement noise when applied to frame structures.

### Two-dimensional shear frame

**Fig. 25:**
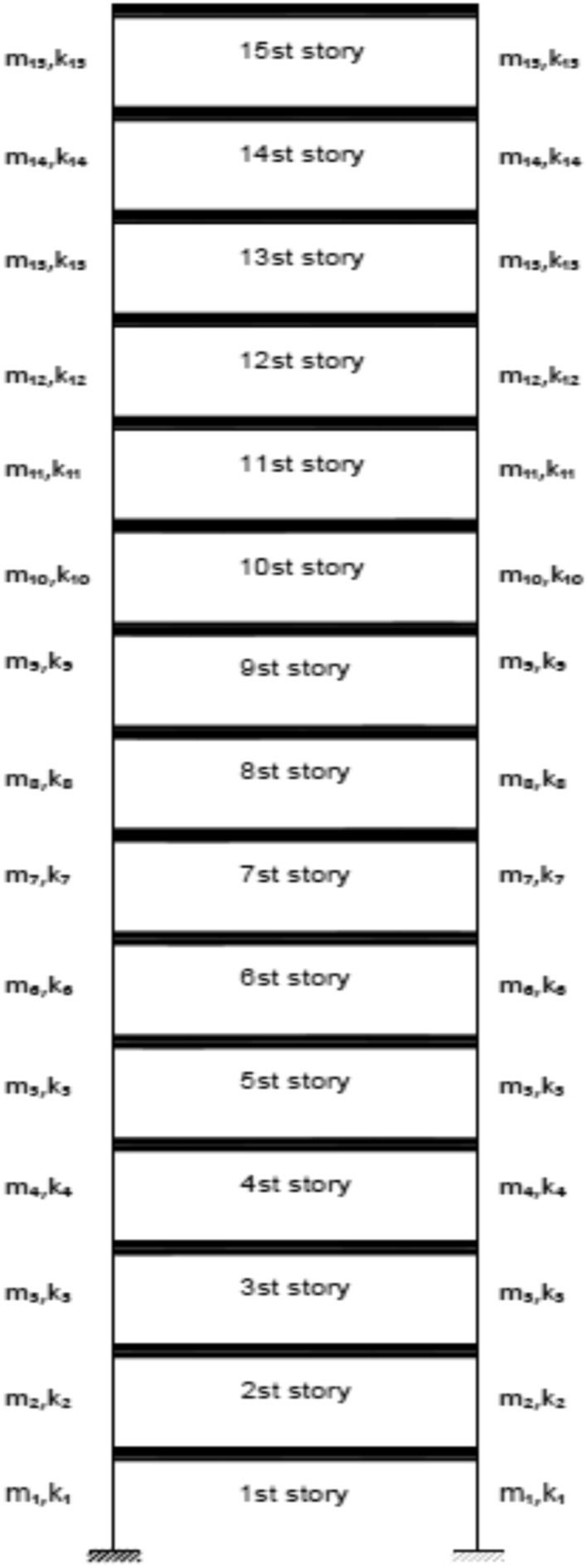
Two-Dimensional Shear Frame.

The structural system in question is represented by a two-dimensional shear frame model, comprising a total of 15 stories, as depicted in Fig. 25. Table 5 presents the material properties, followed by various damage scenarios detailed in Table 6. The parameters of the optimization algorithm are as delineated: 300 is the maximum number of iterations that can occur, the number of the wolf population is specified as 120, with the upper bound at 1 and the lower bound at 0. The results of damage detection under conditions of 0% noise, 3% noise and 8% noise are presented.

**Table 5:** Story detail.

Story number	Story stiffness (N/m)	Story mass (kg)
1	6500	350
2	5500	350
3	5500	550
4	3000	450
5	3000	550
6	4500	350
7	5000	500
8	3500	400
9	4000	550
10	4000	450
11	3500	300
12	4000	350
13	4000	400
14	3500	350
15	4000	400

**Table 6:** Different damage scenarios for the shear frame.

The number of modes	Damage scenario	Damaged element	Damage rate
1	Scenario 1	2	10%
	Scenario 2	4	5%
		8	15%
	Scenario 3	1	10%
		5	25%
		9	15%
		13	20%
3	Scenario 1	2	10%
	Scenario 2	4	5%
		8	15%
	Scenario 3	1	10%
		5	25%
		9	15%
		13	20%

**Fig. 26:**
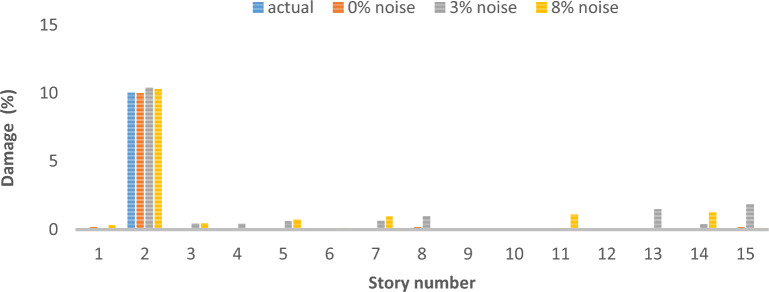
Damage identification results of shear frame in the first damage scenario using the information of the first mode.

**Fig. 27:**
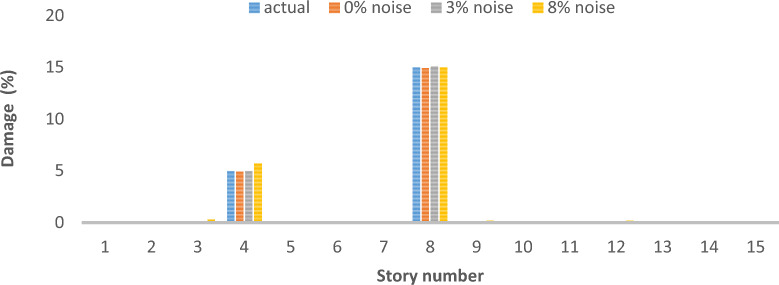
Damage identification results of shear frame in the second damage scenario using the information of the first mode.

**Fig. 28:**
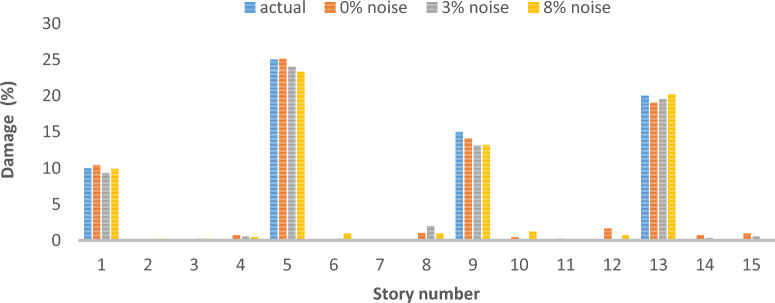
Damage identification results of shear frame in the third damage scenario using the information of the first mode.

**Fig. 29:**
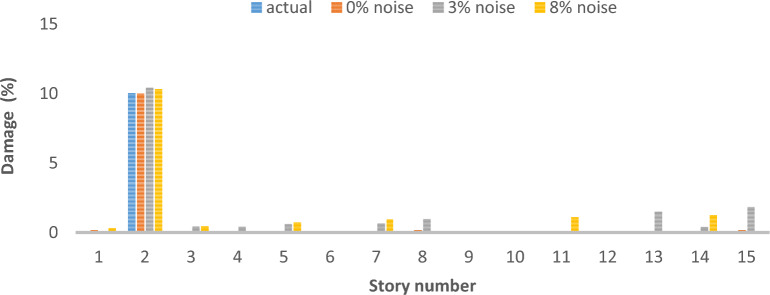
Damage identification results of shear frame in the first damage scenario using the information of the first 3 modes.

**Fig. 30:**
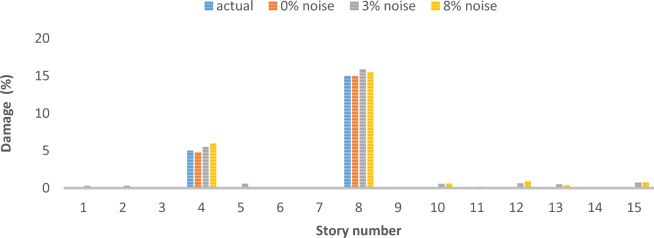
Damage identification results of shear frame in the second damage scenario using the information of the first 3 modes.

**Fig. 31:**
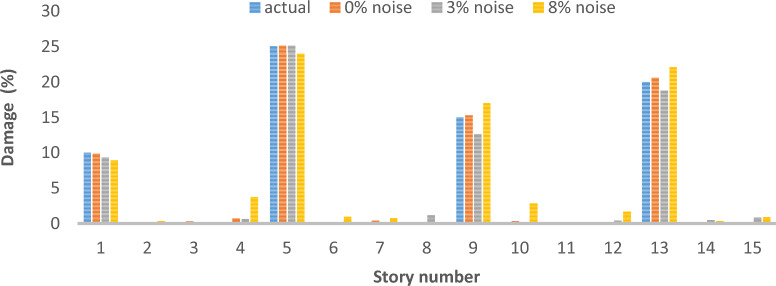
Damage identification results of shear frame in the third damage scenario using the information of the first 3 modes.

As can be seen in Figs. 26, 27, 28, 29, 30, 31, very high accuracy results can be achieved with only 300 repetitions, even considering the effect of noise.

By comparing the results, we can come to the conclusion that in the absence of noise, even using the information of the first mode, we can reach the results with the same accuracy as when we use the information of the first 3 modes. For example, by comparing Figs. 26 and 29, it is quite evident that the results of identifying the 4 damages considered in the third scenario, considering the number of modes (the first 3 modes), have a similar accuracy compared to when the information of the first mode is used.

Another noteworthy point is that, in the presence of negative effects of noise, using higher mode number information increases the negative effects of noise and may lead to more error in damage detection. For example, comparing Figs. 27 and 30, it is quite clear that in the presence of 3% and 8% noise, the damage detection with the first mode is more accurate than the damage detection with the first 3 modes.

**Fig. 32:**
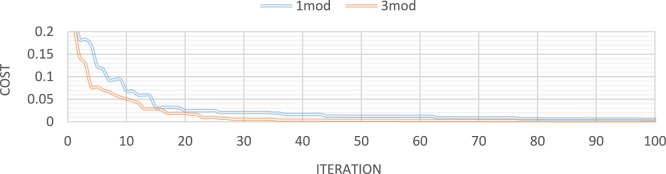
Convergence curve of the first damage scenario in the absence of noise for first 1 and 3 modes.

**Fig. 33:**
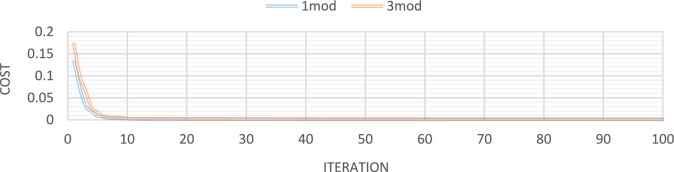
Convergence curve of the first damage scenario in the presence of 8% noise for first 1 and 3 modes.

**Fig. 34:**
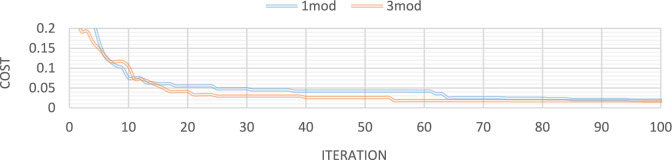
Convergence curve of the third damage scenario in the absence of noise for first 1 and 3 modes.

**Fig. 35:**
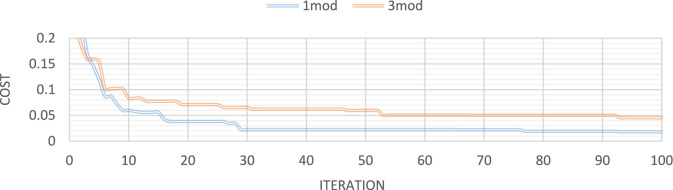
Convergence curve of the third damage scenario in the presence of 8% noise for first 1 and 3 modes.

Figures 32, 33, 34, 35 also display the convergence curve for the inserted goal function. These graphs make it evident that more time is needed to identify damage as the number of damaged structural elements increases, particularly when noise is present.

To evaluate the robustness of the proposed method for the shear frame structure, further simulations were carried out by introducing Gaussian white noise at 10%, 15%, and 20% intensity levels into the strain measurements. The findings show that the proposed approach maintains high damage detection and quantification accuracy for noise levels up to 15%, with only a slight increase in the identification errors. At a 20% noise level, although a moderate reduction in quantitative accuracy is observed, the method still successfully identifies the location of the damaged elements without significant false detections. These results demonstrate that the proposed damage identification framework is capable of delivering reliable performance for shear frame structures, even under relatively high levels of measurement noise.

**Fig. 36:**
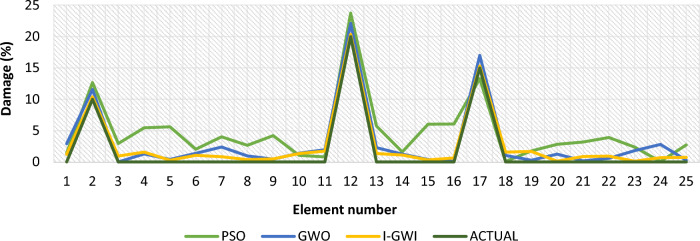
The average results damage detection of twenty independent runs for the PSO, GWO and I-GWO in the third damage scenario of Damage identification of 25-member flat truss, using information of the first 2 modes.

**Fig. 37:**
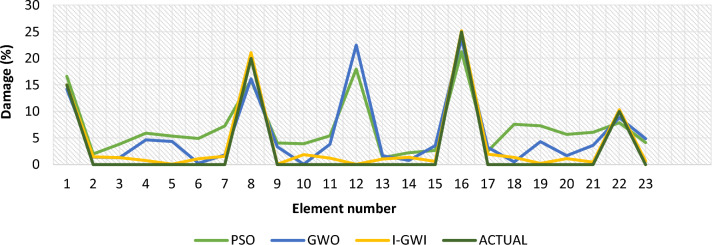
The average results damage detection of twenty independent runs for the PSO, GWO and I-GWO in the third damage scenario of Damage identification of 23-member frame, using information of the first mode.

In order to evaluate the reliability and efficiency of various optimization algorithms, the average results damage detection of twenty independent runs delineated in Fig. 36 for 25 elements truss and Fig. 37 for 23 elements frame, at noise levels of 4%, for the I-GWO, GWO and PSO algorithms.

## Discussion

In this study, a novel damage identification framework combining the generalized flexibility matrix with an Improved Grey Wolf Optimizer (I-GWO) was developed and extensively validated through numerical simulations and experimental verifications. The integration of flexibility-based sensitivity analysis with dynamic characteristics, together with an advanced metaheuristic optimization strategy, enables highly accurate detection and quantification of structural damages, even under significant measurement uncertainties.

A key innovation of this study lies in the formulation of the objective function, which simultaneously integrates generalized flexibility information and natural frequency shifts. The flexibility matrix captures global stiffness degradation, while the frequency shifts reflect localized dynamic changes. The combination of these two complementary indicators significantly enhances the sensitivity and robustness of the damage identification process, particularly in the presence of noise and incomplete data.

The Improved Grey Wolf Optimizer (I-GWO) plays a critical role in the effectiveness of the proposed framework. Unlike the standard GWO, which may suffer from slow convergence and premature trapping in local optima, the I-GWO introduces a dimension learning-based hunting mechanism. This enhancement enables each search agent (wolf) to dynamically adjust its position not only based on the best-known leaders (alpha, beta, delta) but also by learning valuable dimensional information from neighboring solutions. As a result, the optimizer achieves better exploration during the early search phase and stronger exploitation near promising regions in the later stages. Furthermore, the dimension learning strategy helps maintain population diversity, effectively preventing stagnation and promoting a balanced search across the solution space. These improvements contribute to the observed superior convergence speed and solution accuracy compared to standard GWO and other conventional algorithms.

Across various tested structural systems, including truss, frame, and shear frame structures subjected to different noise intensities, the proposed method consistently demonstrated reliable and accurate performance. Damage locations were successfully identified, and quantification errors remained minimal even under elevated noise levels. Such results validate the noise resilience and general applicability of the developed framework for practical structural health monitoring applications.

Nevertheless, a few limitations should be acknowledged. The framework assumes accurate extraction of modal parameters and primarily considers linear structural behavior. Extending the methodology to address nonlinearities such as material nonlinearity, boundary flexibility, and dynamic contact effects is an important direction for future research. Additionally, further investigation under extreme noise conditions would help clarify the boundaries of the method’s robustness.

In summary, the proposed damage identification framework, empowered by the enhanced I-GWO optimization strategy and the dual sensitivity objective function, offers a robust, noise-resistant, and computationally efficient solution for accurate structural damage assessment, demonstrating strong potential for real-world engineering applications.

## Conclusion

In this study, a methodology based on model updating is introduced, where two parameters undergoing updates, namely the flexibility matrix and the structural frequency, are combined with optimization techniques to accomplish structural damage evaluation. Despite the challenges encountered in assessing damages in structures with high degrees of freedom, as well as the introduction of multiple damages across various areas of the structure, incorporating noise, it is postulated that leveraging the advantages of the structural flexibility matrix can enhance the efficacy of the proposed objective function. This can address the limitations associated with the detection of minor and overall damages in frequency-based approaches, particularly when utilizing the initial modes of the structure, leading to a highly accurate assessment of damages.

Additionally, a comparison of multiple studies is conducted to evaluate the stability of the I-GWO approach, including average results from 20 runs, statistical results, and convergence with other evolutionary optimization methods such as PSO and GWO. I-GWO outperforms GWO and PSO algorithms in determining the extent and location of damage, as illustrated in Figs. 34 and 35.

The error observed between the simulated and estimated outcomes for each damage scenario was found to be below 3 percent, thus validating the efficacy of this approach in identifying structural damage in both frames and trusses. Further exploration of the physical model, incorporation of additional updated variables, and integration of novel heuristic and multi-objective algorithms are suggested for enhancing the methodology.

## Supplementary Information

Supplementary Information.

## Data Availability

All data generated or analysed during this study are included in this published article and its supplementary information files.

## References

[1] Law, S., Ni, P. & Li, J. Parallel decentralized damage detection of a structure with subsets of parameters. *AIAA J.* **52**(3), 650–656 (2014).

[2] Gudmundson, P. Eigenfrequency changes of structures due to cracks, notches or other geometrical changes. *J. Mech. Phys. Solids* **30**(5), 339–353 (1982).

[3] Sanayei, M. & Onipede, O. Damage assessment of structures using static test data. *AIAA J.* **29**(7), 1174–1179 (1991).

[4] Wang, X. et al. Structural damage identification using static test data and changes in frequencies. *Eng. Struct.* **23**(6), 610–621 (2001).

[5] Bakhtiari-Nejad, F., Rahai, A. & Esfandiari, A. A structural damage detection method using static noisy data. *Eng. Struct.* **27**(12), 1784–1793 (2005).

[6] Esfandiari, A. et al. Finite Element Model Updating Using Frequency Response Function of Incomplete Strain Data. *AIAA J.* **48**(7), 1420–1433 (2010).

[7] Abdo, M.A.-B. Parametric study of using only static response in structural damage detection. *Eng. Struct.* **34**, 124–131 (2012).

[8] Ni, P. H. & Law, S. S. Hybrid computational strategy for structural damage detection with short-term monitoring data. *Mech. Syst. Signal Process.* **70–71**, 650–663 (2016).

[9] Seyedpoor, S. M. & Yazdanpanah, O. An efficient indicator for structural damage localization using the change of strain energy based on static noisy data. *Appl. Math. Model.* **38**(9), 2661–2672 (2014).

[10] Sanayei, M. et al. Automated finite element model updating of a scale bridge model using measured static and modal test data. *Eng. Struct.* **102**, 66–79 (2015).

[11] Lin, C.-S. Location of modeling errors using modal test data. *AIAA J.* **28**, 1650–1654 (1989).

[12] Zhao, J. & DeWolf, J. T. Sensitivity Study for Vibrational Parameters Used in Damage Detection. *J. Struct. Eng.* **125**(4), 410–416 (1999).

[13] Pandey, A. K. & Biswas, M. Damage Detection in Structures Using Changes in Flexibility. *J. Sound Vib.* **169**(1), 3–17 (1994).

[14] Yan, G., Duan, Z. & Ou, J. Damage detection for truss or frame structures using an axial strain flexibility Smart Structures and Systems. *Inter. J.* **5**(3), 291–316 (2009).

[15] Kim, B. H., Joo, H. J. & Park, T. Damage evaluation of an axially loaded beam using modal flexibility. *KSCE J. Civ. Eng.* **11**, 101–110 (2007).

[16] Li, J. et al. A generalized flexibility matrix based approach for structural damage detection. *J. Sound Vib.* **329**(22), 4583–4587 (2010).

[17] Zhao, B. et al. Structural damage detection by using single natural frequency and the corresponding mode shape. *Shock. Vib.* **2016**(1), 8194549 (2016).

[18] Yan, W.-J. & Ren, W.-X. Closed-form modal flexibility sensitivity and its application to structural damage detection without modal truncation error. *J. Vib. Control.* **20**(12), 1816–1830 (2014).

[19] Xu, L., Wang, X., Wang, Z. & Cao, G. Hybrid quantum genetic algorithm for structural damage identification. *Comput. Methods Appl. Mech. Eng.* **1**(438), 117866 (2025).

[20] Ding, Z. et al. A novel Bayesian empowered piecewise multi-objective sparse evolution for structural condition assessment. *Int. J. Struct. Stab. Dyn.* **21**, 2550101 (2024).

[21] Wan, C., Zhang, G., Xie, L. & Xue, S. Structural damage identification with output-only measurements using modified Jaya algorithm and Tikhonov regularization method Smart Structures and Systems. *Inter. J.* **31**(3), 229–245 (2023).

[22] Zhang, G., Kang, J., Wan, C., Xie, L. & Xue, S. Output-only structural damage identification based on Q-learning hybrid evolutionary algorithm and response reconstruction technique. *Measurement* **1**(224), 113951 (2024).

[23] Alkayem, N. F., Shen, L., Al-hababi, T., Qian, X. & Cao, M. Inverse analysis of structural damage based on the modal kinetic and strain energies with the novel oppositional unified particle swarm gradient-based optimizer. *Appl. Sci.* **12**(22), 11689 (2022).

[24] Alkayem, N. F., Cao, M., Shen, L., Fu, R. & Šumarac, D. The combined social engineering particle swarm optimization for real-world engineering problems: A case study of model-based structural health monitoring. *Appl. Soft Comput.* **1**(123), 108919 (2022).

[25] Ding, Z., Fu, K., Deng, W., Li, J. & Zhongrong, L. A modified Artificial Bee Colony algorithm for structural damage identification under varying temperature based on a novel objective function. *Appl. Math. Model.* **1**(88), 122–141 (2020).

[26] Zhang, G. et al. Structural damage identification with output-only strain measurements and swarm intelligence algorithms: a comparative study. *Meas. Sci. Technol.* **35**(5), 056125 (2024).

[27] Mirjalili, S., Mirjalili, S. M. & Lewis, A. Grey wolf optimizer. *Adv. Eng. Softw.* **69**, 46–61 (2014).

[28] Nadimi-Shahraki, M. H., Taghian, S. & Mirjalili, S. An improved grey wolf optimizer for solving engineering problems. *Expert Syst. Appl.* **166**, 113917 (2021).

[29] MacNulty, D. R., Mech, L. D. & Smith, D. W. A proposed ethogram of large-carnivore predatory behavior, exemplified by the wolf. *J. Mammal.* **88**(3), 595–605 (2007).

[30] Preumont, A. *Vibration control of active structures: an introduction* (Springer, 2018).

[31] Yan, A. & Golinval, J.-C. Structural damage localization by combining flexibility and stiffness methods. *Eng. Struct.* **27**(12), 1752–1761 (2005).

