# Supplementary material for: Model updating method for detect and localize structural damage using generalized flexibility matrix and improved grey wolf optimizer algorithm (I-GWO)
Source: Sci Rep. 2025 Jul 7;15:24300. doi: 10.1038/s41598-025-09499-6 (PMC12234813; doi:10.1038/s41598-025-09499-6)
Supplement: Supplementary file 1 — Supplementary Information. [file 41598_2025_9499_MOESM1_ESM.pdf]

Supplementary

Model Updating Method For Detect and Localize Structural Damage Using Generalized Flexibility Matrix And Improved Grey Wolf Optimizer Algorithm (I-GWO)

Authors: Sina Sadraei[1], Majid Gholhaki* [1], Omid Rezaifar [1]

[1] *Faculty of Civil Engineering, Semnan University, Iran*

***Corresponding Author:** Majid Gholhaki (Mgholhaki@semnan.ac.ir)

E-mail addresses:

Sinasadr2010@gmail.com (S. Sadraei),

Mgholhaki@semnan.ac.ir (M. Gholhaki),

Orezayfar@semnan.ac.ir (O. Rezaifar)

Supplementary Tables:

Table 1	*page 4*
Table 2	*page 5*
Table 3	*page 6*
Table 4	*page 7*
Table 5	*page 8*
Table 6	*page 9*
Table 7	*page 10*
Table 8	*page 17*
Table 9	*page 24*
Table 10	*page 31*
Table 11	*page 38*
Table 12	*page 39*
Table 13	*page 40*
Table 14	*page 41*
Table 15	*page 42*
Table 16	*page 43*
Table 17	*page 44*
Table 18	*page 51*
Table 19	*page 58*
Table 20	*page 65*
Table 21	*page 72*
Table 22	*page 73*
Table 23	*page 74*
Table 24	*page 75*
Table 25	*page 76*
Table 26	*page 77*
Table 27	*page 78*
Table 28	*page 81*

Table 29	*page 84*
Table 30	*page 87*

Table 1. Damage identification results of 25-member flat truss in the first damage scenario using the information of the first 2 modes

Member	Actual Damage	2 Modes 0% Noise	2 Modes 2% Noise	2 Modes 4% Noise
**1**	0	0	0	0.2
**2**	0	0	0.1	0
**3**	0	0	0	0.9
**4**	0	0	0.1	0
**5**	0	0	0.2	0
**6**	0	0	0.3	0.7
**7**	0	0	0	0
**8**	0	0	0	0.1
**9**	0	0	0.1	0.1
**10**	0	0	1	0
**11**	0	0	0	3
**12**	0	0	0	0
**13**	0	0	0.1	0.3
**14**	0	0	0	2.7
**15**	0	0	0.4	0
**16**	0	0	1.4	1.1
**17**	0	0	1	0.8
**18**	0	0	0.8	0
**19**	0	0	0	0
**20**	0	0	0.8	0
**21**	0	0	0	1.1
**22**	0	0	0	0.7
**23**	25	25	24.14	24.59
**24**	0	0	0	0.3
**25**	0	0	0	0.3

Table 2. Damage identification results of 25-member flat truss in the second damage scenario using the information of the first 2 modes

Member	Actual Damage	2 Modes 0% Noise	2 Modes 2% Noise	2 Modes 4% Noise
**1**	0	0	0	0.4
**2**	0	0	0.1	0
**3**	0	0	0	0
**4**	0	0	0.2	1.9
**5**	0	0	0	0.4
**6**	0	0	0.1	0
**7**	0	0	0	0
**8**	0	0	0	0
**9**	0	0	0	0
**10**	5	5	5	4.8
**11**	0	0.1	0.1	0.5
**12**	0	0	0.6	0
**13**	0	0.1	0.3	0.7
**14**	0	0.2	0.1	0.7
**15**	0	0	0	0.1
**16**	0	0.4	0	0.1
**17**	0	0	0	0.5
**18**	15	15	14.97	15.3
**19**	0	0.3	0	1
**20**	0	0	0	0.2
**21**	0	0	0	0.5
**22**	0	0	0	0
**23**	0	0	0	0.2
**24**	0	0	0	0
**25**	0	0	0	0.6

Table 3. Damage identification results of 25-member flat truss in the third damage scenario using the information of the first 2 modes

Member	Actual Damage	2 Modes 0% Noise	2 Modes 2% Noise	2 Modes 4% Noise
**1**	0	0.1	0	0
**2**	10	9.94	8	8.5
**3**	0	0	0	0
**4**	0	0	0	3
**5**	0	0	0	0.1
**6**	0	0	0	0.44
**7**	0	0	0	0
**8**	0	0	0.1	0.2
**9**	0	0	0.3	0
**10**	0	0	0	0
**11**	0	0	0	0
**12**	20	20	22	23
**13**	0	0.1	0.8	0
**14**	0	0.1	0	0
**15**	0	0	2.7	3
**16**	0	0	0	0
**17**	15	15	16.1	15.18
**18**	0	0	0	0
**19**	0	0	0	0
**20**	0	0	0.1	0.16
**21**	0	0	0	0
**22**	0	0	0	0
**23**	0	0	0	0
**24**	0	0	0	0
**25**	0	0	0	0

Table 4. Damage identification results of 25-member flat truss in the first damage scenario using the information of the first 4 modes

Member	Actual Damage	4 Modes 0% Noise	4 Modes 2% Noise	4 Modes 4% Noise
**1**	0	0	0	0
**2**	0	0	0	3
**3**	0	0	1.8	0
**4**	0	0	0.1	0
**5**	0	0	0	0
**6**	0	0	0.2	0.21
**7**	0	0	0	2.09
**8**	0	0	0	0
**9**	0	0	0	0
**10**	0	0	0	0
**11**	0	0	0	1.98
**12**	0	0	0	0.2
**13**	0	0	0	0
**14**	0	0	0	2.8
**15**	0	0	0	0
**16**	0	0	0	0
**17**	0	0	0	2.9
**18**	0	0	0	0.13
**19**	0	0	0	0.13
**20**	0	0	0	0
**21**	0	0	0	0
**22**	0	0	0	0
**23**	25	24.99	24.5	28
**24**	0	0	0.1	0
**25**	0	0	0	0

Table 5. Damage identification results of 25-member flat truss in the second damage scenario using the information of the first 4 modes

Member	Actual Damage	4 Modes 0% Noise	4 Modes 2% Noise	4 Modes 4% Noise
**1**	0	0.1	0.3	0
**2**	0	0	0	1.51
**3**	0	0.1	0.2	0
**4**	0	0	0.1	2.25
**5**	0	0	0	0
**6**	0	0	0	0.37
**7**	0	0	0	0
**8**	0	0	0	0.1
**9**	0	0	0	0
**10**	5	4.98	4.7	5.21
**11**	0	0	0.7	0.3
**12**	0	0	0	0.99
**13**	0	0	0	0
**14**	0	0	0.27	0
**15**	0	0	0	0
**16**	0	0	0	0.59
**17**	0	0	0.49	0.1
**18**	15	14.99	14.85	15.3
**19**	0	0	0	0
**20**	0	0	0	0
**21**	0	0	0	0
**22**	0	0	0	0
**23**	0	0	0.34	1.56
**24**	0	0	0	0
**25**	0	0	0	0

Table 6. Damage identification results of 25-member flat truss in the third damage scenario using the information of the first 4 modes

Member	Actual Damage	4 Modes 0% Noise	4 Modes 2% Noise	4 Modes 4% Noise
**1**	0	0.8	0	0.13
**2**	10	9.74	10.4	13
**3**	0	0.1	2	0
**4**	0	0	0	0
**5**	0	0	0.3	0.31
**6**	0	0	0.6	0
**7**	0	0	0.2	0
**8**	0	0	0	0.19
**9**	0	0	0.01	0.62
**10**	0	0	0	0
**11**	0	0	0.6	0.81
**12**	20	19.71	19.97	19.11
**13**	0	0	0	0.45
**14**	0	0	0	0.33
**15**	0	0	0	0
**16**	0	0.2	0.2	0
**17**	15	15	15.77	16.94
**18**	0	0	0	0
**19**	0	0	0.1	0.23
**20**	0	0	0	0
**21**	0	0	0.3	0
**22**	0	0	0	0.18
**23**	0	0	0	0
**24**	0	0	0	1.54
**25**	0	0	0	0

Table 7. Convergence of the first damage scenario in the absence of noise for first 2 and 4 modes

Iterations	2 Modes	4 Modes	Iterations	2 Modes	4 Modes	Iterations	2 Modes	4 Modes
**1**	0.2213	0.22861	**309**	0.000154	9.73E-05	**617**	8.02E-05	5.80E-05
**2**	0.15578	0.1623	**310**	0.000154	9.73E-05	**618**	8.02E-05	5.80E-05
**3**	0.10962	0.14301	**311**	0.000154	9.73E-05	**619**	8.02E-05	5.80E-05
**4**	0.097916	0.12994	**312**	0.000154	9.73E-05	**620**	8.02E-05	5.80E-05
**5**	0.069792	0.11275	**313**	0.000154	9.73E-05	**621**	8.02E-05	5.80E-05
**6**	0.055236	0.096255	**314**	0.000154	9.73E-05	**622**	8.02E-05	5.80E-05
**7**	0.044092	0.066862	**315**	0.000154	9.73E-05	**623**	8.02E-05	5.80E-05
**8**	0.041324	0.056756	**316**	0.000154	9.73E-05	**624**	8.02E-05	5.80E-05
**9**	0.034444	0.052301	**317**	0.000154	9.73E-05	**625**	8.02E-05	5.80E-05
**10**	0.032506	0.043133	**318**	0.000154	9.73E-05	**626**	8.02E-05	5.80E-05
**11**	0.029135	0.036336	**319**	0.000154	9.73E-05	**627**	8.02E-05	5.80E-05
**12**	0.02625	0.027437	**320**	0.000154	9.73E-05	**628**	8.02E-05	5.80E-05
**13**	0.025683	0.021273	**321**	0.000154	9.73E-05	**629**	8.02E-05	5.80E-05
**14**	0.019253	0.021273	**322**	0.000154	9.73E-05	**630**	8.02E-05	5.80E-05
**15**	0.019253	0.018148	**323**	0.000154	9.73E-05	**631**	8.02E-05	5.80E-05
**16**	0.018025	0.016592	**324**	0.000134	9.73E-05	**632**	8.02E-05	5.80E-05
**17**	0.018025	0.016592	**325**	0.000134	9.73E-05	**633**	8.02E-05	5.80E-05
**18**	0.014437	0.013167	**326**	0.000134	9.73E-05	**634**	8.02E-05	5.80E-05
**19**	0.012907	0.013167	**327**	0.000134	9.73E-05	**635**	8.02E-05	5.80E-05
**20**	0.011989	0.006901	**328**	0.000134	9.73E-05	**636**	8.02E-05	5.80E-05
**21**	0.009146	0.004603	**329**	0.000134	9.73E-05	**637**	8.02E-05	5.80E-05
**22**	0.00901	0.004552	**330**	0.000134	9.73E-05	**638**	8.02E-05	5.80E-05
**23**	0.007809	0.004552	**331**	0.000134	9.73E-05	**639**	8.02E-05	5.80E-05
**24**	0.007439	0.004356	**332**	0.000134	9.73E-05	**640**	8.02E-05	5.80E-05
**25**	0.006948	0.004216	**333**	0.000134	9.73E-05	**641**	8.02E-05	5.80E-05
**26**	0.005287	0.003618	**334**	0.000134	9.73E-05	**642**	8.02E-05	5.80E-05
**27**	0.005287	0.00335	**335**	0.000134	9.73E-05	**643**	8.02E-05	5.80E-05
**28**	0.005287	0.002986	**336**	0.000134	9.73E-05	**644**	8.02E-05	5.80E-05
**29**	0.005058	0.002986	**337**	0.000134	9.73E-05	**645**	8.02E-05	5.80E-05
**30**	0.004682	0.002986	**338**	0.000134	9.73E-05	**646**	8.02E-05	5.80E-05
**31**	0.00408	0.002986	**339**	0.000134	9.73E-05	**647**	8.02E-05	5.80E-05
**32**	0.003208	0.002986	**340**	0.000134	9.73E-05	**648**	8.02E-05	5.80E-05
**33**	0.003208	0.002986	**341**	0.000134	9.73E-05	**649**	8.02E-05	5.80E-05
**34**	0.003208	0.002986	**342**	0.000134	9.73E-05	**650**	8.02E-05	5.80E-05
**35**	0.003208	0.002805	**343**	0.000134	9.73E-05	**651**	8.02E-05	5.80E-05
**36**	0.002635	0.002007	**344**	0.000134	9.73E-05	**652**	8.02E-05	5.80E-05
**37**	0.002635	0.001845	**345**	0.000134	9.73E-05	**653**	8.02E-05	5.80E-05
**38**	0.002635	0.001845	**346**	0.000134	9.73E-05	**654**	8.02E-05	5.80E-05
**39**	0.002635	0.001767	**347**	0.000134	9.73E-05	**655**	8.02E-05	5.80E-05
**40**	0.002635	0.001767	**348**	0.000134	9.73E-05	**656**	8.02E-05	5.80E-05
**41**	0.002635	0.001767	**349**	0.000134	9.73E-05	**657**	8.02E-05	5.80E-05
**42**	0.002635	0.001767	**350**	0.000134	9.73E-05	**658**	8.02E-05	5.80E-05
**43**	0.002635	0.001609	**351**	0.000134	9.73E-05	**659**	8.02E-05	5.80E-05
**44**	0.002346	0.001609	**352**	0.000134	9.73E-05	**660**	8.02E-05	5.80E-05

**45**	0.002346	0.001609	**353**	0.000134	9.73E-05	**661**	8.02E-05	5.80E-05
**46**	0.001869	0.001609	**354**	0.000134	9.73E-05	**662**	8.02E-05	5.80E-05
**47**	0.001869	0.001609	**355**	0.000134	9.73E-05	**663**	8.02E-05	5.80E-05
**48**	0.001869	0.001609	**356**	0.000134	9.73E-05	**664**	8.02E-05	5.43E-05
**49**	0.001869	0.001609	**357**	0.000134	9.73E-05	**665**	8.02E-05	5.43E-05
**50**	0.001869	0.001609	**358**	0.000134	9.73E-05	**666**	8.02E-05	5.43E-05
**51**	0.001869	0.001609	**359**	0.000134	9.73E-05	**667**	8.02E-05	5.43E-05
**52**	0.001869	0.001609	**360**	0.000134	9.73E-05	**668**	8.02E-05	5.43E-05
**53**	0.001869	0.001607	**361**	0.000134	9.73E-05	**669**	8.02E-05	5.43E-05
**54**	0.001769	0.001607	**362**	0.000134	9.73E-05	**670**	8.02E-05	5.43E-05
**55**	0.001365	0.001607	**363**	0.000134	9.73E-05	**671**	8.02E-05	5.43E-05
**56**	0.001365	0.001607	**364**	0.000134	9.73E-05	**672**	8.02E-05	5.43E-05
**57**	0.001365	0.001483	**365**	0.000134	9.73E-05	**673**	8.02E-05	5.43E-05
**58**	0.001365	0.001483	**366**	0.000134	9.73E-05	**674**	8.02E-05	5.43E-05
**59**	0.001365	0.001483	**367**	0.000134	9.73E-05	**675**	8.02E-05	5.43E-05
**60**	0.001365	0.001483	**368**	0.000134	9.73E-05	**676**	8.02E-05	5.43E-05
**61**	0.001365	0.001483	**369**	0.000134	9.73E-05	**677**	8.02E-05	5.43E-05
**62**	0.001365	0.001483	**370**	0.000134	9.73E-05	**678**	8.02E-05	5.43E-05
**63**	0.001313	0.001483	**371**	0.000134	9.73E-05	**679**	8.02E-05	5.43E-05
**64**	0.001313	0.001483	**372**	0.000134	9.73E-05	**680**	8.02E-05	5.43E-05
**65**	0.001233	0.001288	**373**	0.000134	9.73E-05	**681**	8.02E-05	5.43E-05
**66**	0.001233	0.001288	**374**	0.000134	9.73E-05	**682**	8.02E-05	5.43E-05
**67**	0.001233	0.001288	**375**	0.000134	9.73E-05	**683**	8.02E-05	5.43E-05
**68**	0.001233	0.001288	**376**	0.000134	9.73E-05	**684**	8.02E-05	5.43E-05
**69**	0.001233	0.001288	**377**	0.000134	9.73E-05	**685**	8.02E-05	5.09E-05
**70**	0.001233	0.001288	**378**	0.000134	9.73E-05	**686**	8.02E-05	5.09E-05
**71**	0.0011	0.001288	**379**	0.000134	9.73E-05	**687**	8.02E-05	5.09E-05
**72**	0.0011	0.001288	**380**	0.000134	9.73E-05	**688**	8.02E-05	5.09E-05
**73**	0.0011	0.001288	**381**	0.00012	9.73E-05	**689**	8.02E-05	5.09E-05
**74**	0.000963	0.001288	**382**	0.00012	9.73E-05	**690**	8.02E-05	5.09E-05
**75**	0.000963	0.001288	**383**	0.00012	9.73E-05	**691**	8.02E-05	5.09E-05
**76**	0.000963	0.001288	**384**	0.00012	9.73E-05	**692**	8.02E-05	5.09E-05
**77**	0.000806	0.001105	**385**	0.00012	9.73E-05	**693**	8.02E-05	5.09E-05
**78**	0.000806	0.00105	**386**	0.00012	9.73E-05	**694**	8.02E-05	5.09E-05
**79**	0.000806	0.00105	**387**	0.00012	9.73E-05	**695**	8.02E-05	5.09E-05
**80**	0.000806	0.00105	**388**	0.00012	9.73E-05	**696**	8.02E-05	5.09E-05
**81**	0.000806	0.00105	**389**	0.00012	9.73E-05	**697**	8.02E-05	5.09E-05
**82**	0.000783	0.00105	**390**	0.00012	9.73E-05	**698**	7.54E-05	5.09E-05
**83**	0.000783	0.00105	**391**	0.00012	9.73E-05	**699**	6.66E-05	5.09E-05
**84**	0.000783	0.00105	**392**	0.00012	9.73E-05	**700**	6.66E-05	5.09E-05
**85**	0.000678	0.00105	**393**	0.00012	9.73E-05	**701**	6.66E-05	5.09E-05
**86**	0.000678	0.000946	**394**	0.00012	9.73E-05	**702**	6.66E-05	5.09E-05
**87**	0.000678	0.000946	**395**	0.00012	9.73E-05	**703**	6.66E-05	5.09E-05
**88**	0.000678	0.000946	**396**	0.00012	9.73E-05	**704**	6.66E-05	5.09E-05
**89**	0.000678	0.000946	**397**	0.00012	9.73E-05	**705**	6.66E-05	5.09E-05
**90**	0.000678	0.000702	**398**	0.00012	9.73E-05	**706**	6.66E-05	5.09E-05

**91**	0.000678	0.000702	**399**	0.00012	9.73E-05	**707**	6.66E-05	5.09E-05
**92**	0.000678	0.000702	**400**	0.00012	9.73E-05	**708**	6.66E-05	5.09E-05
**93**	0.000678	0.000702	**401**	0.00012	9.73E-05	**709**	6.66E-05	5.09E-05
**94**	0.000678	0.000702	**402**	0.00012	9.73E-05	**710**	6.66E-05	5.09E-05
**95**	0.000678	0.000702	**403**	0.00012	9.73E-05	**711**	6.66E-05	5.09E-05
**96**	0.000678	0.000702	**404**	0.00012	9.73E-05	**712**	6.66E-05	5.09E-05
**97**	0.000678	0.000702	**405**	0.00012	9.73E-05	**713**	6.66E-05	5.09E-05
**98**	0.000678	0.000655	**406**	0.00012	9.73E-05	**714**	6.66E-05	5.09E-05
**99**	0.000678	0.000608	**407**	0.00012	9.73E-05	**715**	6.66E-05	5.09E-05
**100**	0.000678	0.000608	**408**	0.00012	9.73E-05	**716**	6.66E-05	5.09E-05
**101**	0.000678	0.000608	**409**	0.00012	9.73E-05	**717**	6.66E-05	5.09E-05
**102**	0.000678	0.000608	**410**	0.00012	9.73E-05	**718**	6.66E-05	5.09E-05
**103**	0.000678	0.000608	**411**	0.000117	9.73E-05	**719**	6.66E-05	5.09E-05
**104**	0.000678	0.000608	**412**	0.000117	9.73E-05	**720**	6.66E-05	5.09E-05
**105**	0.000678	0.000548	**413**	0.000117	9.73E-05	**721**	6.66E-05	5.09E-05
**106**	0.000522	0.000548	**414**	0.000117	9.73E-05	**722**	6.66E-05	5.09E-05
**107**	0.000522	0.000548	**415**	0.000117	9.73E-05	**723**	6.66E-05	5.09E-05
**108**	0.000522	0.000548	**416**	0.000117	9.73E-05	**724**	6.66E-05	5.09E-05
**109**	0.000522	0.000548	**417**	0.000117	9.73E-05	**725**	6.66E-05	5.09E-05
**110**	0.000522	0.000548	**418**	0.000117	9.73E-05	**726**	6.66E-05	5.09E-05
**111**	0.000522	0.000537	**419**	0.000117	9.73E-05	**727**	6.66E-05	5.09E-05
**112**	0.000522	0.000537	**420**	0.000117	9.73E-05	**728**	6.66E-05	5.09E-05
**113**	0.000522	0.000537	**421**	0.000117	9.73E-05	**729**	6.66E-05	5.09E-05
**114**	0.000522	0.000537	**422**	0.000117	9.73E-05	**730**	6.66E-05	5.09E-05
**115**	0.000522	0.000537	**423**	0.000117	9.60E-05	**731**	6.66E-05	5.09E-05
**116**	0.000522	0.000537	**424**	0.000117	9.60E-05	**732**	6.66E-05	5.09E-05
**117**	0.000522	0.000537	**425**	0.000117	9.60E-05	**733**	6.66E-05	5.09E-05
**118**	0.000485	0.000537	**426**	0.000117	9.60E-05	**734**	6.66E-05	5.09E-05
**119**	0.000485	0.000537	**427**	0.000117	9.60E-05	**735**	6.66E-05	5.09E-05
**120**	0.000485	0.000537	**428**	0.000117	9.60E-05	**736**	6.66E-05	5.09E-05
**121**	0.000485	0.000537	**429**	0.000117	9.60E-05	**737**	6.66E-05	5.09E-05
**122**	0.000485	0.000537	**430**	0.000117	9.60E-05	**738**	6.66E-05	5.09E-05
**123**	0.000485	0.000537	**431**	0.000117	9.60E-05	**739**	6.66E-05	5.09E-05
**124**	0.000485	0.000537	**432**	0.000117	9.60E-05	**740**	6.66E-05	5.09E-05
**125**	0.000485	0.000537	**433**	0.000117	9.60E-05	**741**	6.66E-05	5.09E-05
**126**	0.000485	0.000537	**434**	0.000117	9.60E-05	**742**	6.66E-05	5.09E-05
**127**	0.000485	0.000537	**435**	0.000117	9.60E-05	**743**	6.66E-05	5.09E-05
**128**	0.000485	0.000537	**436**	0.000117	9.60E-05	**744**	6.66E-05	5.09E-05
**129**	0.000485	0.000537	**437**	0.000117	9.60E-05	**745**	6.66E-05	5.09E-05
**130**	0.000485	0.000537	**438**	0.000115	9.60E-05	**746**	6.66E-05	5.09E-05
**131**	0.000485	0.000537	**439**	0.000115	9.60E-05	**747**	6.66E-05	5.09E-05
**132**	0.000485	0.000537	**440**	0.000115	9.60E-05	**748**	6.66E-05	5.09E-05
**133**	0.000485	0.000537	**441**	0.000115	9.60E-05	**749**	6.66E-05	5.09E-05
**134**	0.000485	0.000537	**442**	0.000115	9.60E-05	**750**	6.66E-05	5.09E-05
**135**	0.000485	0.000537	**443**	0.000115	9.60E-05	**751**	6.66E-05	5.09E-05
**136**	0.000485	0.000537	**444**	0.000115	9.60E-05	**752**	6.66E-05	5.09E-05

**137**	0.000485	0.000537	**445**	0.000115	9.60E-05	**753**	6.66E-05	5.09E-05
**138**	0.000485	0.000537	**446**	0.000115	9.60E-05	**754**	6.66E-05	5.09E-05
**139**	0.000485	0.000537	**447**	0.000115	9.60E-05	**755**	6.66E-05	5.09E-05
**140**	0.000485	0.000537	**448**	0.000115	9.60E-05	**756**	6.66E-05	5.09E-05
**141**	0.000485	0.000537	**449**	0.000115	9.60E-05	**757**	6.66E-05	5.09E-05
**142**	0.000485	0.000537	**450**	0.000115	9.60E-05	**758**	6.66E-05	5.09E-05
**143**	0.000485	0.000537	**451**	0.000115	9.60E-05	**759**	6.66E-05	5.09E-05
**144**	0.000485	0.000448	**452**	0.000115	9.60E-05	**760**	6.66E-05	5.09E-05
**145**	0.000485	0.000448	**453**	0.000115	9.60E-05	**761**	6.66E-05	5.09E-05
**146**	0.000485	0.000448	**454**	0.000115	9.60E-05	**762**	6.66E-05	5.09E-05
**147**	0.000403	0.000448	**455**	0.000115	9.60E-05	**763**	6.66E-05	5.09E-05
**148**	0.000403	0.000448	**456**	0.000115	9.60E-05	**764**	6.66E-05	5.09E-05
**149**	0.000403	0.000448	**457**	0.000115	9.60E-05	**765**	6.66E-05	5.09E-05
**150**	0.000403	0.000448	**458**	0.000115	8.68E-05	**766**	6.66E-05	5.09E-05
**151**	0.000403	0.000448	**459**	0.000115	8.68E-05	**767**	6.66E-05	5.09E-05
**152**	0.000403	0.000448	**460**	0.000115	8.68E-05	**768**	6.66E-05	4.87E-05
**153**	0.000403	0.000448	**461**	0.000115	8.68E-05	**769**	6.66E-05	4.87E-05
**154**	0.000403	0.000448	**462**	0.000115	8.68E-05	**770**	6.66E-05	4.87E-05
**155**	0.000403	0.000448	**463**	0.000115	8.68E-05	**771**	6.66E-05	4.87E-05
**156**	0.000403	0.000448	**464**	0.000115	8.68E-05	**772**	6.66E-05	4.87E-05
**157**	0.000403	0.000448	**465**	0.000115	8.68E-05	**773**	6.66E-05	4.87E-05
**158**	0.000403	0.000448	**466**	0.000115	8.68E-05	**774**	6.66E-05	4.87E-05
**159**	0.000403	0.000448	**467**	0.000115	8.68E-05	**775**	6.66E-05	4.87E-05
**160**	0.000403	0.000448	**468**	0.000115	8.68E-05	**776**	6.66E-05	4.87E-05
**161**	0.000403	0.000448	**469**	0.000115	8.68E-05	**777**	6.66E-05	4.87E-05
**162**	0.000403	0.000448	**470**	0.000115	8.68E-05	**778**	6.66E-05	4.87E-05
**163**	0.000403	0.000448	**471**	0.000115	8.68E-05	**779**	6.66E-05	4.87E-05
**164**	0.000403	0.000448	**472**	0.000115	8.68E-05	**780**	6.66E-05	4.87E-05
**165**	0.000403	0.000448	**473**	0.000115	8.23E-05	**781**	6.66E-05	4.87E-05
**166**	0.000403	0.000448	**474**	0.000115	8.23E-05	**782**	6.66E-05	4.87E-05
**167**	0.000403	0.000448	**475**	0.000115	8.23E-05	**783**	6.66E-05	4.87E-05
**168**	0.000403	0.000448	**476**	0.000115	8.23E-05	**784**	6.66E-05	4.75E-05
**169**	0.000403	0.000448	**477**	0.000115	8.23E-05	**785**	6.66E-05	4.75E-05
**170**	0.000403	0.000448	**478**	0.000115	8.23E-05	**786**	6.66E-05	4.75E-05
**171**	0.000403	0.000448	**479**	0.000115	8.23E-05	**787**	6.66E-05	4.75E-05
**172**	0.000403	0.000448	**480**	0.000115	8.23E-05	**788**	6.66E-05	4.75E-05
**173**	0.000403	0.000448	**481**	0.000115	8.23E-05	**789**	6.66E-05	4.75E-05
**174**	0.000403	0.000448	**482**	0.000115	8.23E-05	**790**	6.66E-05	4.75E-05
**175**	0.000403	0.000448	**483**	0.000115	8.23E-05	**791**	6.66E-05	4.75E-05
**176**	0.000403	0.000448	**484**	0.000115	8.23E-05	**792**	6.66E-05	4.75E-05
**177**	0.000403	0.000448	**485**	0.000115	8.23E-05	**793**	6.66E-05	4.75E-05
**178**	0.000403	0.000448	**486**	0.000115	8.23E-05	**794**	6.66E-05	4.75E-05
**179**	0.000304	0.000369	**487**	0.000115	8.23E-05	**795**	6.66E-05	4.75E-05
**180**	0.000304	0.000369	**488**	0.000115	8.23E-05	**796**	6.66E-05	4.75E-05
**181**	0.000304	0.000369	**489**	0.000115	8.23E-05	**797**	6.66E-05	4.75E-05
**182**	0.000304	0.000369	**490**	0.000115	8.23E-05	**798**	6.66E-05	4.75E-05

**183**	0.000304	0.00026	**491**	9.88E-05	8.23E-05	**799**	6.66E-05	4.75E-05
**184**	0.000304	0.00026	**492**	9.88E-05	8.23E-05	**800**	6.66E-05	4.75E-05
**185**	0.000304	0.00026	**493**	9.88E-05	8.23E-05	**801**	6.66E-05	4.75E-05
**186**	0.000304	0.00026	**494**	9.88E-05	8.23E-05	**802**	6.66E-05	4.75E-05
**187**	0.000304	0.00026	**495**	9.88E-05	8.23E-05	**803**	6.66E-05	4.75E-05
**188**	0.000304	0.00026	**496**	9.88E-05	8.23E-05	**804**	6.66E-05	4.75E-05
**189**	0.000304	0.00026	**497**	9.88E-05	8.23E-05	**805**	6.66E-05	4.75E-05
**190**	0.000304	0.00026	**498**	9.88E-05	8.23E-05	**806**	6.66E-05	4.75E-05
**191**	0.000304	0.00024	**499**	9.88E-05	8.23E-05	**807**	6.66E-05	4.75E-05
**192**	0.000304	0.00024	**500**	9.88E-05	8.23E-05	**808**	6.66E-05	4.75E-05
**193**	0.000304	0.000221	**501**	9.88E-05	8.23E-05	**809**	6.66E-05	4.75E-05
**194**	0.000304	0.000221	**502**	9.88E-05	8.23E-05	**810**	6.66E-05	4.75E-05
**195**	0.000304	0.000221	**503**	8.28E-05	8.23E-05	**811**	6.66E-05	4.75E-05
**196**	0.000304	0.000221	**504**	8.28E-05	8.23E-05	**812**	6.66E-05	4.75E-05
**197**	0.000304	0.000221	**505**	8.28E-05	8.23E-05	**813**	6.47E-05	4.75E-05
**198**	0.000304	0.000221	**506**	8.28E-05	6.18E-05	**814**	6.47E-05	4.75E-05
**199**	0.000304	0.000221	**507**	8.28E-05	6.18E-05	**815**	6.47E-05	4.75E-05
**200**	0.000304	0.000221	**508**	8.28E-05	6.18E-05	**816**	6.47E-05	4.75E-05
**201**	0.000304	0.000221	**509**	8.28E-05	6.18E-05	**817**	6.47E-05	4.75E-05
**202**	0.000304	0.000221	**510**	8.28E-05	6.18E-05	**818**	6.46E-05	4.75E-05
**203**	0.000304	0.000221	**511**	8.28E-05	6.18E-05	**819**	6.46E-05	4.75E-05
**204**	0.000304	0.000221	**512**	8.28E-05	6.18E-05	**820**	6.46E-05	4.75E-05
**205**	0.000304	0.000221	**513**	8.28E-05	6.18E-05	**821**	6.46E-05	4.75E-05
**206**	0.000304	0.000221	**514**	8.28E-05	6.18E-05	**822**	6.46E-05	4.75E-05
**207**	0.000304	0.000221	**515**	8.28E-05	6.18E-05	**823**	6.46E-05	4.75E-05
**208**	0.000304	0.000221	**516**	8.28E-05	6.18E-05	**824**	6.46E-05	4.75E-05
**209**	0.000304	0.000221	**517**	8.28E-05	6.18E-05	**825**	6.46E-05	4.75E-05
**210**	0.000304	0.000221	**518**	8.28E-05	6.18E-05	**826**	6.46E-05	4.75E-05
**211**	0.000304	0.000201	**519**	8.28E-05	6.18E-05	**827**	6.46E-05	4.75E-05
**212**	0.000288	0.000201	**520**	8.28E-05	6.18E-05	**828**	6.46E-05	4.75E-05
**213**	0.000288	0.000201	**521**	8.28E-05	6.18E-05	**829**	6.46E-05	4.75E-05
**214**	0.000288	0.000201	**522**	8.28E-05	6.18E-05	**830**	6.46E-05	4.75E-05
**215**	0.000288	0.000201	**523**	8.28E-05	6.18E-05	**831**	6.46E-05	4.75E-05
**216**	0.000288	0.000201	**524**	8.28E-05	6.18E-05	**832**	6.46E-05	4.75E-05
**217**	0.000288	0.000201	**525**	8.28E-05	6.18E-05	**833**	6.46E-05	4.75E-05
**218**	0.000288	0.000201	**526**	8.28E-05	6.18E-05	**834**	6.46E-05	4.75E-05
**219**	0.000288	0.000201	**527**	8.28E-05	6.18E-05	**835**	6.46E-05	4.75E-05
**220**	0.000288	0.000201	**528**	8.28E-05	6.18E-05	**836**	6.46E-05	4.75E-05
**221**	0.000288	0.000201	**529**	8.28E-05	6.18E-05	**837**	6.46E-05	4.75E-05
**222**	0.000288	0.000201	**530**	8.28E-05	6.18E-05	**838**	6.46E-05	4.75E-05
**223**	0.000288	0.000201	**531**	8.28E-05	6.18E-05	**839**	6.46E-05	4.75E-05
**224**	0.000288	0.000201	**532**	8.28E-05	6.18E-05	**840**	5.96E-05	4.75E-05
**225**	0.000288	0.000201	**533**	8.28E-05	6.18E-05	**841**	5.96E-05	4.75E-05
**226**	0.000288	0.000201	**534**	8.28E-05	6.18E-05	**842**	5.96E-05	4.75E-05
**227**	0.000288	0.000201	**535**	8.28E-05	6.18E-05	**843**	5.96E-05	4.75E-05
**228**	0.000288	0.000201	**536**	8.28E-05	6.18E-05	**844**	5.96E-05	4.75E-05

**229**	0.000288	0.000201	**537**	8.28E-05	6.18E-05	**845**	5.96E-05	4.75E-05
**230**	0.000288	0.000201	**538**	8.28E-05	6.18E-05	**846**	5.96E-05	4.75E-05
**231**	0.000288	0.000201	**539**	8.28E-05	6.18E-05	**847**	5.96E-05	4.75E-05
**232**	0.000288	0.000201	**540**	8.28E-05	6.18E-05	**848**	5.96E-05	4.75E-05
**233**	0.000288	0.000201	**541**	8.28E-05	6.18E-05	**849**	5.96E-05	4.75E-05
**234**	0.000288	0.000201	**542**	8.28E-05	6.18E-05	**850**	5.96E-05	4.75E-05
**235**	0.000288	0.000201	**543**	8.28E-05	6.18E-05	**851**	5.96E-05	4.75E-05
**236**	0.000288	0.000201	**544**	8.28E-05	6.18E-05	**852**	5.96E-05	4.75E-05
**237**	0.000288	0.000201	**545**	8.28E-05	6.18E-05	**853**	5.96E-05	4.75E-05
**238**	0.000288	0.000186	**546**	8.28E-05	6.18E-05	**854**	5.96E-05	4.75E-05
**239**	0.000288	0.000186	**547**	8.02E-05	6.18E-05	**855**	5.96E-05	4.75E-05
**240**	0.000288	0.000186	**548**	8.02E-05	6.18E-05	**856**	5.96E-05	4.75E-05
**241**	0.000288	0.000186	**549**	8.02E-05	6.18E-05	**857**	5.96E-05	4.75E-05
**242**	0.000288	0.00012	**550**	8.02E-05	6.18E-05	**858**	5.96E-05	4.75E-05
**243**	0.000288	0.00012	**551**	8.02E-05	6.18E-05	**859**	5.96E-05	4.75E-05
**244**	0.000278	0.00012	**552**	8.02E-05	6.18E-05	**860**	5.96E-05	4.75E-05
**245**	0.000278	0.00012	**553**	8.02E-05	6.18E-05	**861**	5.96E-05	4.75E-05
**246**	0.000278	0.00012	**554**	8.02E-05	6.18E-05	**862**	5.96E-05	4.75E-05
**247**	0.000278	0.00012	**555**	8.02E-05	6.18E-05	**863**	5.96E-05	4.75E-05
**248**	0.000278	0.00012	**556**	8.02E-05	6.18E-05	**864**	5.96E-05	4.57E-05
**249**	0.000278	0.00012	**557**	8.02E-05	6.18E-05	**865**	5.96E-05	4.57E-05
**250**	0.000278	0.00012	**558**	8.02E-05	6.18E-05	**866**	5.96E-05	4.57E-05
**251**	0.000278	0.00012	**559**	8.02E-05	6.18E-05	**867**	5.96E-05	4.57E-05
**252**	0.000278	0.00012	**560**	8.02E-05	6.18E-05	**868**	5.96E-05	4.57E-05
**253**	0.000278	0.00012	**561**	8.02E-05	6.18E-05	**869**	5.96E-05	4.57E-05
**254**	0.000278	0.00012	**562**	8.02E-05	6.18E-05	**870**	5.96E-05	4.57E-05
**255**	0.000278	0.00012	**563**	8.02E-05	6.18E-05	**871**	5.96E-05	4.57E-05
**256**	0.000278	0.00012	**564**	8.02E-05	6.18E-05	**872**	5.96E-05	4.57E-05
**257**	0.000278	0.00012	**565**	8.02E-05	6.18E-05	**873**	5.96E-05	4.57E-05
**258**	0.000278	0.00012	**566**	8.02E-05	6.18E-05	**874**	5.96E-05	4.57E-05
**259**	0.000203	0.00012	**567**	8.02E-05	6.18E-05	**875**	5.96E-05	4.57E-05
**260**	0.000203	0.00012	**568**	8.02E-05	6.18E-05	**876**	5.96E-05	4.57E-05
**261**	0.000203	0.00012	**569**	8.02E-05	6.18E-05	**877**	5.96E-05	4.57E-05
**262**	0.000203	0.00012	**570**	8.02E-05	6.18E-05	**878**	5.96E-05	4.57E-05
**263**	0.000203	0.00012	**571**	8.02E-05	6.18E-05	**879**	5.96E-05	4.57E-05
**264**	0.000203	0.00012	**572**	8.02E-05	6.18E-05	**880**	5.96E-05	4.57E-05
**265**	0.000203	0.00012	**573**	8.02E-05	6.18E-05	**881**	5.96E-05	4.57E-05
**266**	0.000203	0.00012	**574**	8.02E-05	6.18E-05	**882**	5.96E-05	4.57E-05
**267**	0.000203	0.00012	**575**	8.02E-05	6.18E-05	**883**	5.96E-05	4.57E-05
**268**	0.000203	0.00012	**576**	8.02E-05	6.18E-05	**884**	5.96E-05	4.57E-05
**269**	0.000203	0.00012	**577**	8.02E-05	6.18E-05	**885**	5.96E-05	4.57E-05
**270**	0.000203	0.00012	**578**	8.02E-05	6.18E-05	**886**	5.96E-05	4.57E-05
**271**	0.000203	0.000102	**579**	8.02E-05	6.18E-05	**887**	5.96E-05	4.57E-05
**272**	0.000203	0.000102	**580**	8.02E-05	6.18E-05	**888**	5.96E-05	4.57E-05
**273**	0.000203	0.000102	**581**	8.02E-05	6.18E-05	**889**	5.96E-05	4.57E-05
**274**	0.000203	0.000102	**582**	8.02E-05	6.18E-05	**890**	5.96E-05	4.57E-05

**275**	0.000203	0.000102	**583**	8.02E-05	6.18E-05	**891**	5.96E-05	4.57E-05
**276**	0.000203	0.000102	**584**	8.02E-05	6.18E-05	**892**	5.96E-05	4.57E-05
**277**	0.000203	9.73E-05	**585**	8.02E-05	6.18E-05	**893**	5.96E-05	4.57E-05
**278**	0.000203	9.73E-05	**586**	8.02E-05	6.18E-05	**894**	5.96E-05	4.57E-05
**279**	0.000203	9.73E-05	**587**	8.02E-05	6.18E-05	**895**	5.96E-05	4.57E-05
**280**	0.000203	9.73E-05	**588**	8.02E-05	6.18E-05	**896**	5.96E-05	4.57E-05
**281**	0.000203	9.73E-05	**589**	8.02E-05	6.18E-05	**897**	5.96E-05	4.57E-05
**282**	0.000203	9.73E-05	**590**	8.02E-05	6.18E-05	**898**	5.96E-05	4.57E-05
**283**	0.000203	9.73E-05	**591**	8.02E-05	5.80E-05	**899**	5.91E-05	4.57E-05
**284**	0.000203	9.73E-05	**592**	8.02E-05	5.80E-05	**900**	5.91E-05	4.57E-05
**285**	0.000203	9.73E-05	**593**	8.02E-05	5.80E-05			
**286**	0.000203	9.73E-05	**594**	8.02E-05	5.80E-05			
**287**	0.000203	9.73E-05	**595**	8.02E-05	5.80E-05			
**288**	0.000203	9.73E-05	**596**	8.02E-05	5.80E-05			
**289**	0.000203	9.73E-05	**597**	8.02E-05	5.80E-05			
**290**	0.000203	9.73E-05	**598**	8.02E-05	5.80E-05			
**291**	0.000203	9.73E-05	**599**	8.02E-05	5.80E-05			
**292**	0.000203	9.73E-05	**600**	8.02E-05	5.80E-05			
**293**	0.000203	9.73E-05	**601**	8.02E-05	5.80E-05			
**294**	0.000203	9.73E-05	**602**	8.02E-05	5.80E-05			
**295**	0.000154	9.73E-05	**603**	8.02E-05	5.80E-05			
**296**	0.000154	9.73E-05	**604**	8.02E-05	5.80E-05			
**297**	0.000154	9.73E-05	**605**	8.02E-05	5.80E-05			
**298**	0.000154	9.73E-05	**606**	8.02E-05	5.80E-05			
**299**	0.000154	9.73E-05	**607**	8.02E-05	5.80E-05			
**300**	0.000154	9.73E-05	**608**	8.02E-05	5.80E-05			
**301**	0.000154	9.73E-05	**609**	8.02E-05	5.80E-05			
**302**	0.000154	9.73E-05	**610**	8.02E-05	5.80E-05			
**303**	0.000154	9.73E-05	**611**	8.02E-05	5.80E-05			
**304**	0.000154	9.73E-05	**612**	8.02E-05	5.80E-05			
**305**	0.000154	9.73E-05	**613**	8.02E-05	5.80E-05			
**306**	0.000154	9.73E-05	**614**	8.02E-05	5.80E-05			
**307**	0.000154	9.73E-05	**615**	8.02E-05	5.80E-05			
**308**	0.000154	9.73E-05	**616**	8.02E-05	5.80E-05			

Table 8. Convergence of the first damage scenario in the presence of 4% noise for first 2 and 4 modes

Iterations	2 Modes	4 Modes	Iterations	2 Modes	4 Modes	Iterations	2 Modes	4 Modes
**1**	0.15307	0.22197	**309**	0.007365	0.035696	**617**	0.005715	0.035669
**2**	0.10515	0.17179	**310**	0.007365	0.035696	**618**	0.005715	0.035669
**3**	0.10515	0.16998	**311**	0.007365	0.035696	**619**	0.005715	0.035669
**4**	0.10042	0.1621	**312**	0.007365	0.035696	**620**	0.005715	0.035669
**5**	0.097776	0.10034	**313**	0.007365	0.035696	**621**	0.005715	0.035669
**6**	0.074549	0.084347	**314**	0.007365	0.035696	**622**	0.005715	0.035669
**7**	0.051818	0.069722	**315**	0.007365	0.035696	**623**	0.005715	0.035669
**8**	0.051818	0.061828	**316**	0.007365	0.035696	**624**	0.005715	0.035669
**9**	0.051818	0.059062	**317**	0.007365	0.035696	**625**	0.005715	0.035669
**10**	0.051818	0.050558	**318**	0.007365	0.035696	**626**	0.005715	0.035669
**11**	0.051818	0.050558	**319**	0.007365	0.035696	**627**	0.005715	0.035669
**12**	0.047019	0.04735	**320**	0.007365	0.035696	**628**	0.005715	0.035669
**13**	0.037845	0.044202	**321**	0.007365	0.035696	**629**	0.005715	0.035669
**14**	0.037845	0.043118	**322**	0.007118	0.035696	**630**	0.005715	0.035669
**15**	0.032269	0.042276	**323**	0.007118	0.035696	**631**	0.005715	0.035667
**16**	0.032269	0.040739	**324**	0.007118	0.035696	**632**	0.005715	0.035667
**17**	0.022806	0.040739	**325**	0.007118	0.035696	**633**	0.005641	0.035667
**18**	0.022806	0.038983	**326**	0.007118	0.035696	**634**	0.005641	0.035665
**19**	0.022806	0.038722	**327**	0.007118	0.035696	**635**	0.005641	0.035665
**20**	0.022806	0.03813	**328**	0.007118	0.035696	**636**	0.005641	0.035665
**21**	0.022806	0.038053	**329**	0.007118	0.035696	**637**	0.005641	0.035665
**22**	0.020092	0.038053	**330**	0.007118	0.035696	**638**	0.005641	0.035665
**23**	0.019571	0.038053	**331**	0.007118	0.035696	**639**	0.005641	0.035665
**24**	0.019571	0.038053	**332**	0.007118	0.035696	**640**	0.005641	0.035665
**25**	0.015236	0.038053	**333**	0.007118	0.035696	**641**	0.005641	0.035665
**26**	0.015042	0.038017	**334**	0.007118	0.035696	**642**	0.005641	0.035665
**27**	0.013074	0.038017	**335**	0.007118	0.035696	**643**	0.005641	0.035665
**28**	0.013074	0.038017	**336**	0.007118	0.035696	**644**	0.005641	0.035665
**29**	0.013074	0.037776	**337**	0.007118	0.035696	**645**	0.005641	0.035665
**30**	0.013074	0.037776	**338**	0.007118	0.035696	**646**	0.005641	0.035665
**31**	0.013074	0.037776	**339**	0.007118	0.035696	**647**	0.005641	0.035665
**32**	0.012596	0.037738	**340**	0.007118	0.035696	**648**	0.005641	0.035665
**33**	0.012596	0.037738	**341**	0.007118	0.035696	**649**	0.005641	0.035665
**34**	0.012596	0.037615	**342**	0.007118	0.035696	**650**	0.005641	0.035665
**35**	0.012596	0.037141	**343**	0.007118	0.035696	**651**	0.005641	0.035665
**36**	0.012596	0.037141	**344**	0.007118	0.035696	**652**	0.005641	0.035665
**37**	0.012596	0.037141	**345**	0.007118	0.035696	**653**	0.005641	0.035665
**38**	0.012596	0.037141	**346**	0.007118	0.035696	**654**	0.005641	0.035665
**39**	0.012596	0.037031	**347**	0.006544	0.035696	**655**	0.005641	0.035665
**40**	0.012596	0.037031	**348**	0.006544	0.035696	**656**	0.005641	0.035665
**41**	0.012596	0.037031	**349**	0.006544	0.035696	**657**	0.005641	0.035665
**42**	0.012596	0.036852	**350**	0.006544	0.035696	**658**	0.005641	0.035665
**43**	0.012596	0.036852	**351**	0.006544	0.035696	**659**	0.005641	0.035665
**44**	0.012596	0.03644	**352**	0.006544	0.035696	**660**	0.005641	0.035665

**45**	0.012596	0.03644	**353**	0.006544	0.035696	**661**	0.005641	0.035665
**46**	0.012596	0.036425	**354**	0.006544	0.035696	**662**	0.005641	0.035665
**47**	0.012596	0.03626	**355**	0.006544	0.035696	**663**	0.005641	0.035665
**48**	0.009245	0.03626	**356**	0.006544	0.035696	**664**	0.005641	0.035665
**49**	0.009245	0.03626	**357**	0.006544	0.035696	**665**	0.005641	0.035665
**50**	0.009245	0.036222	**358**	0.006544	0.035696	**666**	0.005641	0.035665
**51**	0.009245	0.036222	**359**	0.006544	0.035696	**667**	0.005641	0.035665
**52**	0.009245	0.036222	**360**	0.006544	0.035696	**668**	0.005641	0.035665
**53**	0.009245	0.036222	**361**	0.006544	0.035696	**669**	0.005641	0.035665
**54**	0.009245	0.036139	**362**	0.006544	0.035696	**670**	0.005641	0.035635
**55**	0.009245	0.036139	**363**	0.006544	0.035696	**671**	0.005641	0.035635
**56**	0.009245	0.036139	**364**	0.006544	0.035696	**672**	0.005641	0.035635
**57**	0.009245	0.036139	**365**	0.006387	0.035696	**673**	0.005641	0.035635
**58**	0.009245	0.036139	**366**	0.006387	0.035696	**674**	0.005641	0.035635
**59**	0.009245	0.036139	**367**	0.006387	0.035696	**675**	0.005641	0.035635
**60**	0.009245	0.036139	**368**	0.006387	0.035696	**676**	0.005641	0.035635
**61**	0.009245	0.036139	**369**	0.006387	0.035696	**677**	0.005641	0.035635
**62**	0.009245	0.036139	**370**	0.006387	0.035696	**678**	0.005641	0.035635
**63**	0.009245	0.036139	**371**	0.006387	0.035696	**679**	0.005641	0.035635
**64**	0.009245	0.036139	**372**	0.006387	0.035696	**680**	0.005641	0.035635
**65**	0.009245	0.036139	**373**	0.006387	0.035696	**681**	0.005641	0.035635
**66**	0.009245	0.036139	**374**	0.006387	0.035696	**682**	0.005641	0.035635
**67**	0.009245	0.036139	**375**	0.006387	0.035696	**683**	0.005641	0.035635
**68**	0.009245	0.036139	**376**	0.006387	0.035696	**684**	0.005641	0.035635
**69**	0.009245	0.036139	**377**	0.006387	0.035696	**685**	0.005641	0.035635
**70**	0.009245	0.036139	**378**	0.006387	0.035696	**686**	0.005641	0.035635
**71**	0.009245	0.036136	**379**	0.006387	0.035696	**687**	0.005641	0.035635
**72**	0.009245	0.036136	**380**	0.006387	0.035696	**688**	0.005641	0.035635
**73**	0.009245	0.036136	**381**	0.006387	0.035696	**689**	0.005641	0.035635
**74**	0.009245	0.036136	**382**	0.006387	0.035696	**690**	0.005641	0.035635
**75**	0.009245	0.036136	**383**	0.006387	0.035696	**691**	0.005641	0.035635
**76**	0.009245	0.036136	**384**	0.006387	0.035696	**692**	0.005641	0.035635
**77**	0.009245	0.036136	**385**	0.006387	0.035696	**693**	0.005641	0.035635
**78**	0.009245	0.036136	**386**	0.006387	0.035696	**694**	0.005641	0.035635
**79**	0.009245	0.036136	**387**	0.006387	0.035696	**695**	0.005641	0.035635
**80**	0.009245	0.036136	**388**	0.006387	0.035696	**696**	0.005641	0.035635
**81**	0.009245	0.036135	**389**	0.006387	0.035696	**697**	0.005641	0.035635
**82**	0.009245	0.035984	**390**	0.006387	0.035696	**698**	0.005641	0.035635
**83**	0.009245	0.035984	**391**	0.006387	0.035696	**699**	0.005641	0.035635
**84**	0.009245	0.035984	**392**	0.006387	0.035696	**700**	0.005641	0.035635
**85**	0.009245	0.035984	**393**	0.006387	0.035696	**701**	0.005641	0.035635
**86**	0.009245	0.035984	**394**	0.006387	0.035696	**702**	0.005641	0.035635
**87**	0.009245	0.035983	**395**	0.006387	0.035696	**703**	0.005641	0.035635
**88**	0.009245	0.035983	**396**	0.006387	0.035696	**704**	0.005641	0.035635
**89**	0.008319	0.035983	**397**	0.006387	0.035696	**705**	0.005641	0.035635
**90**	0.008319	0.035983	**398**	0.006387	0.035696	**706**	0.005641	0.035612

**91**	0.008319	0.035852	**399**	0.006387	0.035696	**707**	0.005641	0.035612
**92**	0.008319	0.035852	**400**	0.006387	0.035696	**708**	0.005641	0.035612
**93**	0.008319	0.035852	**401**	0.006387	0.035696	**709**	0.005641	0.035612
**94**	0.008319	0.035852	**402**	0.006358	0.035696	**710**	0.005641	0.035612
**95**	0.008319	0.035852	**403**	0.006358	0.035696	**711**	0.005641	0.035612
**96**	0.008319	0.035852	**404**	0.006358	0.035696	**712**	0.005641	0.035612
**97**	0.008319	0.035852	**405**	0.006358	0.035696	**713**	0.005641	0.035612
**98**	0.008319	0.035852	**406**	0.006358	0.035696	**714**	0.005641	0.035612
**99**	0.008319	0.035852	**407**	0.006358	0.035696	**715**	0.005641	0.035612
**100**	0.008319	0.035852	**408**	0.006358	0.035696	**716**	0.005641	0.035612
**101**	0.008319	0.035852	**409**	0.006358	0.035696	**717**	0.005641	0.035612
**102**	0.008319	0.035852	**410**	0.006358	0.035696	**718**	0.005641	0.035612
**103**	0.008319	0.035852	**411**	0.006358	0.035696	**719**	0.005641	0.035612
**104**	0.008319	0.035852	**412**	0.006358	0.035696	**720**	0.005641	0.035612
**105**	0.008319	0.035852	**413**	0.006358	0.035696	**721**	0.005641	0.035612
**106**	0.008319	0.035852	**414**	0.006358	0.035696	**722**	0.005641	0.035612
**107**	0.008319	0.035852	**415**	0.006358	0.035696	**723**	0.005641	0.035612
**108**	0.008319	0.035852	**416**	0.006358	0.035696	**724**	0.005641	0.035612
**109**	0.008319	0.035852	**417**	0.006358	0.035696	**725**	0.005641	0.035612
**110**	0.008319	0.035851	**418**	0.006358	0.035696	**726**	0.005641	0.035612
**111**	0.008319	0.035851	**419**	0.006358	0.035696	**727**	0.005641	0.035612
**112**	0.008319	0.035851	**420**	0.006358	0.035696	**728**	0.005641	0.035612
**113**	0.008319	0.035851	**421**	0.006358	0.035696	**729**	0.005641	0.035612
**114**	0.008319	0.035851	**422**	0.006358	0.035696	**730**	0.005641	0.035612
**115**	0.008319	0.035851	**423**	0.006358	0.035696	**731**	0.005641	0.035612
**116**	0.008319	0.035851	**424**	0.006358	0.035696	**732**	0.005641	0.035612
**117**	0.008319	0.035851	**425**	0.006358	0.035696	**733**	0.005641	0.035612
**118**	0.008319	0.035851	**426**	0.006358	0.035696	**734**	0.005641	0.035612
**119**	0.008319	0.035851	**427**	0.006358	0.035696	**735**	0.005641	0.035612
**120**	0.008319	0.035851	**428**	0.006358	0.035696	**736**	0.005641	0.035612
**121**	0.008319	0.035851	**429**	0.006358	0.035696	**737**	0.005641	0.035612
**122**	0.008319	0.035851	**430**	0.006358	0.035696	**738**	0.005641	0.035612
**123**	0.008319	0.035851	**431**	0.006358	0.035696	**739**	0.005641	0.035612
**124**	0.008319	0.035851	**432**	0.006358	0.035696	**740**	0.005641	0.035612
**125**	0.008319	0.035842	**433**	0.006358	0.035696	**741**	0.005641	0.035612
**126**	0.008319	0.035842	**434**	0.006358	0.035696	**742**	0.005641	0.035612
**127**	0.008319	0.035842	**435**	0.006282	0.035696	**743**	0.005641	0.035612
**128**	0.008319	0.035842	**436**	0.006282	0.035696	**744**	0.005641	0.035612
**129**	0.008319	0.035842	**437**	0.006282	0.035696	**745**	0.005641	0.035612
**130**	0.008319	0.035842	**438**	0.006282	0.035696	**746**	0.005641	0.035612
**131**	0.008319	0.035842	**439**	0.006282	0.035696	**747**	0.005641	0.035612
**132**	0.008319	0.035842	**440**	0.006282	0.035696	**748**	0.005641	0.035612
**133**	0.008319	0.035842	**441**	0.006282	0.035696	**749**	0.005641	0.035612
**134**	0.008319	0.035842	**442**	0.006282	0.035696	**750**	0.005641	0.035612
**135**	0.008319	0.035842	**443**	0.006282	0.035696	**751**	0.005641	0.035612
**136**	0.008319	0.035842	**444**	0.006282	0.035696	**752**	0.005641	0.035612

**137**	0.008319	0.035841	**445**	0.006282	0.035696	**753**	0.005641	0.035612
**138**	0.008319	0.035841	**446**	0.006282	0.035696	**754**	0.005641	0.035612
**139**	0.008319	0.035841	**447**	0.006282	0.035696	**755**	0.005641	0.035612
**140**	0.008319	0.035841	**448**	0.006282	0.035696	**756**	0.005641	0.035612
**141**	0.008319	0.035841	**449**	0.006282	0.035696	**757**	0.005641	0.035612
**142**	0.008319	0.035841	**450**	0.006282	0.035696	**758**	0.005641	0.035612
**143**	0.008319	0.035841	**451**	0.006282	0.035696	**759**	0.005641	0.035612
**144**	0.008319	0.035841	**452**	0.006282	0.035696	**760**	0.005641	0.035612
**145**	0.008319	0.035841	**453**	0.006282	0.035696	**761**	0.005641	0.035612
**146**	0.008319	0.035841	**454**	0.006282	0.035696	**762**	0.005641	0.035612
**147**	0.008319	0.035841	**455**	0.006282	0.035696	**763**	0.005641	0.035612
**148**	0.008319	0.035841	**456**	0.006282	0.035669	**764**	0.005641	0.035612
**149**	0.008319	0.035841	**457**	0.006282	0.035669	**765**	0.005641	0.035612
**150**	0.008319	0.035841	**458**	0.006282	0.035669	**766**	0.005641	0.035612
**151**	0.008319	0.035841	**459**	0.006282	0.035669	**767**	0.005641	0.035612
**152**	0.008319	0.035841	**460**	0.006282	0.035669	**768**	0.005641	0.035612
**153**	0.008319	0.035841	**461**	0.006282	0.035669	**769**	0.005641	0.035612
**154**	0.008319	0.035824	**462**	0.006282	0.035669	**770**	0.005641	0.035612
**155**	0.008319	0.035824	**463**	0.006282	0.035669	**771**	0.005641	0.035612
**156**	0.008319	0.035824	**464**	0.006282	0.035669	**772**	0.005641	0.035612
**157**	0.008319	0.035824	**465**	0.006282	0.035669	**773**	0.005641	0.035612
**158**	0.008319	0.035824	**466**	0.006282	0.035669	**774**	0.005641	0.035612
**159**	0.008319	0.03579	**467**	0.006282	0.035669	**775**	0.005641	0.035612
**160**	0.008319	0.03579	**468**	0.006282	0.035669	**776**	0.005641	0.035612
**161**	0.008319	0.03579	**469**	0.006282	0.035669	**777**	0.005641	0.035612
**162**	0.008319	0.03579	**470**	0.006282	0.035669	**778**	0.005641	0.035612
**163**	0.008319	0.03579	**471**	0.006282	0.035669	**779**	0.005641	0.035612
**164**	0.008319	0.03579	**472**	0.006047	0.035669	**780**	0.005641	0.035612
**165**	0.008319	0.03579	**473**	0.006047	0.035669	**781**	0.005641	0.035612
**166**	0.008319	0.03579	**474**	0.006047	0.035669	**782**	0.005641	0.035612
**167**	0.008319	0.03579	**475**	0.006047	0.035669	**783**	0.005641	0.035612
**168**	0.008319	0.03579	**476**	0.006047	0.035669	**784**	0.005482	0.035612
**169**	0.008319	0.03579	**477**	0.006047	0.035669	**785**	0.005482	0.035612
**170**	0.008319	0.03579	**478**	0.006047	0.035669	**786**	0.005482	0.035612
**171**	0.008319	0.03579	**479**	0.006047	0.035669	**787**	0.005482	0.035612
**172**	0.008319	0.03579	**480**	0.006047	0.035669	**788**	0.005482	0.035612
**173**	0.008319	0.03579	**481**	0.006047	0.035669	**789**	0.005482	0.035612
**174**	0.008319	0.03579	**482**	0.006047	0.035669	**790**	0.005482	0.035612
**175**	0.008319	0.03579	**483**	0.006047	0.035669	**791**	0.005482	0.035612
**176**	0.008319	0.03579	**484**	0.006047	0.035669	**792**	0.005482	0.035612
**177**	0.008319	0.03579	**485**	0.006047	0.035669	**793**	0.005482	0.035612
**178**	0.008319	0.03579	**486**	0.006047	0.035669	**794**	0.005482	0.035612
**179**	0.008319	0.03579	**487**	0.006047	0.035669	**795**	0.005482	0.035612
**180**	0.008319	0.03579	**488**	0.006047	0.035669	**796**	0.005482	0.035612
**181**	0.008319	0.03579	**489**	0.006047	0.035669	**797**	0.005482	0.035612
**182**	0.008319	0.03579	**490**	0.006047	0.035669	**798**	0.005482	0.035612

**183**	0.008319	0.03579	**491**	0.006047	0.035669	**799**	0.005482	0.035612
**184**	0.008319	0.03579	**492**	0.006047	0.035669	**800**	0.005482	0.035612
**185**	0.008319	0.03579	**493**	0.006047	0.035669	**801**	0.005482	0.035612
**186**	0.008047	0.03579	**494**	0.006047	0.035669	**802**	0.005482	0.035612
**187**	0.008047	0.03579	**495**	0.006047	0.035669	**803**	0.005482	0.035612
**188**	0.008047	0.03579	**496**	0.006047	0.035669	**804**	0.005482	0.035612
**189**	0.008047	0.03579	**497**	0.006047	0.035669	**805**	0.005482	0.035612
**190**	0.008047	0.03579	**498**	0.006047	0.035669	**806**	0.005482	0.035612
**191**	0.008047	0.03579	**499**	0.006047	0.035669	**807**	0.005482	0.035612
**192**	0.008047	0.03579	**500**	0.006047	0.035669	**808**	0.005482	0.035612
**193**	0.008047	0.03579	**501**	0.006047	0.035669	**809**	0.005482	0.035612
**194**	0.008047	0.03579	**502**	0.006047	0.035669	**810**	0.005482	0.035612
**195**	0.008047	0.03579	**503**	0.006047	0.035669	**811**	0.005482	0.035612
**196**	0.008047	0.03579	**504**	0.006047	0.035669	**812**	0.005482	0.035612
**197**	0.008047	0.03579	**505**	0.006047	0.035669	**813**	0.005482	0.035612
**198**	0.008047	0.03579	**506**	0.006047	0.035669	**814**	0.005482	0.035612
**199**	0.008047	0.03579	**507**	0.006047	0.035669	**815**	0.005482	0.035612
**200**	0.008047	0.03579	**508**	0.006047	0.035669	**816**	0.005482	0.035612
**201**	0.008047	0.03579	**509**	0.006047	0.035669	**817**	0.005482	0.035612
**202**	0.008047	0.03579	**510**	0.006047	0.035669	**818**	0.005482	0.035612
**203**	0.008047	0.03579	**511**	0.006047	0.035669	**819**	0.005482	0.035612
**204**	0.008047	0.03579	**512**	0.006047	0.035669	**820**	0.005482	0.035612
**205**	0.008047	0.03579	**513**	0.006047	0.035669	**821**	0.005482	0.035612
**206**	0.008047	0.03579	**514**	0.006047	0.035669	**822**	0.005482	0.035612
**207**	0.008047	0.03579	**515**	0.006047	0.035669	**823**	0.005482	0.035612
**208**	0.008047	0.03579	**516**	0.006047	0.035669	**824**	0.005482	0.035612
**209**	0.008047	0.03579	**517**	0.006047	0.035669	**825**	0.005482	0.035612
**210**	0.008047	0.03579	**518**	0.006047	0.035669	**826**	0.005482	0.035612
**211**	0.008047	0.03579	**519**	0.006047	0.035669	**827**	0.005482	0.035612
**212**	0.008047	0.03579	**520**	0.006047	0.035669	**828**	0.005482	0.035612
**213**	0.008047	0.03579	**521**	0.006047	0.035669	**829**	0.005482	0.035612
**214**	0.008047	0.03579	**522**	0.006047	0.035669	**830**	0.005482	0.035612
**215**	0.008033	0.03579	**523**	0.006047	0.035669	**831**	0.005482	0.035612
**216**	0.008033	0.03579	**524**	0.005994	0.035669	**832**	0.005482	0.035612
**217**	0.008033	0.03579	**525**	0.005994	0.035669	**833**	0.005482	0.035612
**218**	0.008033	0.03579	**526**	0.005994	0.035669	**834**	0.005482	0.035612
**219**	0.008033	0.03579	**527**	0.005994	0.035669	**835**	0.005482	0.035612
**220**	0.008033	0.03579	**528**	0.005994	0.035669	**836**	0.005482	0.035612
**221**	0.008033	0.03579	**529**	0.005994	0.035669	**837**	0.005482	0.035612
**222**	0.008033	0.03579	**530**	0.005994	0.035669	**838**	0.005482	0.035612
**223**	0.008033	0.03579	**531**	0.005994	0.035669	**839**	0.005482	0.035612
**224**	0.008033	0.03579	**532**	0.005994	0.035669	**840**	0.005482	0.035612
**225**	0.008033	0.03579	**533**	0.005994	0.035669	**841**	0.005482	0.035612
**226**	0.008033	0.035696	**534**	0.005994	0.035669	**842**	0.005482	0.035612
**227**	0.008033	0.035696	**535**	0.005994	0.035669	**843**	0.005482	0.035612
**228**	0.008033	0.035696	**536**	0.005994	0.035669	**844**	0.005482	0.035612

**229**	0.008033	0.035696	**537**	0.005994	0.035669	**845**	0.005482	0.035612
**230**	0.008033	0.035696	**538**	0.005994	0.035669	**846**	0.005482	0.035612
**231**	0.008033	0.035696	**539**	0.005994	0.035669	**847**	0.005482	0.035612
**232**	0.008033	0.035696	**540**	0.005994	0.035669	**848**	0.005482	0.035612
**233**	0.008033	0.035696	**541**	0.005994	0.035669	**849**	0.005482	0.035612
**234**	0.008033	0.035696	**542**	0.005994	0.035669	**850**	0.005482	0.035612
**235**	0.008033	0.035696	**543**	0.005994	0.035669	**851**	0.005482	0.035612
**236**	0.008033	0.035696	**544**	0.005799	0.035669	**852**	0.005482	0.035612
**237**	0.008033	0.035696	**545**	0.005799	0.035669	**853**	0.005482	0.035612
**238**	0.008033	0.035696	**546**	0.005799	0.035669	**854**	0.005482	0.035612
**239**	0.008033	0.035696	**547**	0.005799	0.035669	**855**	0.005482	0.035612
**240**	0.008033	0.035696	**548**	0.005799	0.035669	**856**	0.005482	0.035612
**241**	0.008033	0.035696	**549**	0.005799	0.035669	**857**	0.005482	0.035612
**242**	0.008033	0.035696	**550**	0.005799	0.035669	**858**	0.005482	0.035612
**243**	0.008033	0.035696	**551**	0.005799	0.035669	**859**	0.005482	0.035612
**244**	0.008033	0.035696	**552**	0.005799	0.035669	**860**	0.005482	0.035612
**245**	0.008033	0.035696	**553**	0.005799	0.035669	**861**	0.005482	0.035612
**246**	0.008033	0.035696	**554**	0.005799	0.035669	**862**	0.005482	0.035612
**247**	0.008033	0.035696	**555**	0.005799	0.035669	**863**	0.005482	0.035612
**248**	0.008033	0.035696	**556**	0.005799	0.035669	**864**	0.005482	0.035612
**249**	0.008033	0.035696	**557**	0.005799	0.035669	**865**	0.005482	0.035612
**250**	0.008033	0.035696	**558**	0.005799	0.035669	**866**	0.005482	0.035612
**251**	0.008033	0.035696	**559**	0.005799	0.035669	**867**	0.005482	0.035612
**252**	0.008033	0.035696	**560**	0.005799	0.035669	**868**	0.005482	0.035612
**253**	0.008033	0.035696	**561**	0.005799	0.035669	**869**	0.005482	0.035612
**254**	0.008033	0.035696	**562**	0.005799	0.035669	**870**	0.005482	0.035612
**255**	0.008033	0.035696	**563**	0.005799	0.035669	**871**	0.005482	0.035612
**256**	0.008033	0.035696	**564**	0.005799	0.035669	**872**	0.005482	0.035612
**257**	0.008033	0.035696	**565**	0.005799	0.035669	**873**	0.005482	0.035612
**258**	0.008033	0.035696	**566**	0.005799	0.035669	**874**	0.005482	0.035612
**259**	0.008033	0.035696	**567**	0.005799	0.035669	**875**	0.005482	0.035612
**260**	0.008033	0.035696	**568**	0.005799	0.035669	**876**	0.005482	0.035612
**261**	0.008033	0.035696	**569**	0.005799	0.035669	**877**	0.005482	0.035612
**262**	0.008033	0.035696	**570**	0.005799	0.035669	**878**	0.005482	0.035612
**263**	0.008033	0.035696	**571**	0.005799	0.035669	**879**	0.005482	0.035597
**264**	0.008033	0.035696	**572**	0.005799	0.035669	**880**	0.005482	0.035597
**265**	0.008033	0.035696	**573**	0.005799	0.035669	**881**	0.005482	0.035597
**266**	0.008033	0.035696	**574**	0.005799	0.035669	**882**	0.005482	0.035597
**267**	0.008033	0.035696	**575**	0.005799	0.035669	**883**	0.005482	0.035597
**268**	0.008033	0.035696	**576**	0.005799	0.035669	**884**	0.005482	0.035597
**269**	0.008033	0.035696	**577**	0.005799	0.035669	**885**	0.005482	0.035597
**270**	0.008033	0.035696	**578**	0.005799	0.035669	**886**	0.005482	0.035597
**271**	0.008033	0.035696	**579**	0.005799	0.035669	**887**	0.005482	0.035597
**272**	0.008033	0.035696	**580**	0.005799	0.035669	**888**	0.005482	0.035597
**273**	0.008033	0.035696	**581**	0.005799	0.035669	**889**	0.005482	0.035597
**274**	0.007821	0.035696	**582**	0.005799	0.035669	**890**	0.005482	0.035597

**275**	0.007821	0.035696	**583**	0.005799	0.035669	**891**	0.005482	0.035597
**276**	0.007821	0.035696	**584**	0.005799	0.035669	**892**	0.005482	0.035597
**277**	0.007821	0.035696	**585**	0.005799	0.035669	**893**	0.005482	0.035597
**278**	0.007821	0.035696	**586**	0.005799	0.035669	**894**	0.005482	0.035597
**279**	0.007821	0.035696	**587**	0.005799	0.035669	**895**	0.005482	0.035597
**280**	0.007821	0.035696	**588**	0.005799	0.035669	**896**	0.005482	0.035597
**281**	0.007821	0.035696	**589**	0.005799	0.035669	**897**	0.005482	0.035597
**282**	0.007821	0.035696	**590**	0.005799	0.035669	**898**	0.005482	0.035597
**283**	0.007821	0.035696	**591**	0.005799	0.035669	**899**	0.005482	0.035597
**284**	0.007821	0.035696	**592**	0.005799	0.035669	**900**	0.005482	0.035597
**285**	0.007821	0.035696	**593**	0.005799	0.035669			
**286**	0.007821	0.035696	**594**	0.00577	0.035669			
**287**	0.007821	0.035696	**595**	0.00577	0.035669			
**288**	0.007821	0.035696	**596**	0.00577	0.035669			
**289**	0.007821	0.035696	**597**	0.00577	0.035669			
**290**	0.007821	0.035696	**598**	0.005715	0.035669			
**291**	0.007821	0.035696	**599**	0.005715	0.035669			
**292**	0.007821	0.035696	**600**	0.005715	0.035669			
**293**	0.007646	0.035696	**601**	0.005715	0.035669			
**294**	0.007646	0.035696	**602**	0.005715	0.035669			
**295**	0.007646	0.035696	**603**	0.005715	0.035669			
**296**	0.007646	0.035696	**604**	0.005715	0.035669			
**297**	0.007646	0.035696	**605**	0.005715	0.035669			
**298**	0.007646	0.035696	**606**	0.005715	0.035669			
**299**	0.007646	0.035696	**607**	0.005715	0.035669			
**300**	0.007646	0.035696	**608**	0.005715	0.035669			
**301**	0.007646	0.035696	**609**	0.005715	0.035669			
**302**	0.007365	0.035696	**610**	0.005715	0.035669			
**303**	0.007365	0.035696	**611**	0.005715	0.035669			
**304**	0.007365	0.035696	**612**	0.005715	0.035669			
**305**	0.007365	0.035696	**613**	0.005715	0.035669			
**306**	0.007365	0.035696	**614**	0.005715	0.035669			
**307**	0.007365	0.035696	**615**	0.005715	0.035669			
**308**	0.007365	0.035696	**616**	0.005715	0.035669			

Table 9. Convergence of the third damage scenario in the absence of noise for first 2 and 4 modes

Iterations	2 Modes	4 Modes	Iterations	2 Modes	4 Modes	Iterations	2 Modes	4 Modes
**1**	0.21623	0.24914	**309**	0.000327	0.000368	**617**	0.000212	0.000278
**2**	0.12883	0.18718	**310**	0.000327	0.000368	**618**	0.000212	0.000278
**3**	0.11009	0.14369	**311**	0.000327	0.000368	**619**	0.000212	0.000278
**4**	0.10934	0.13804	**312**	0.000327	0.000368	**620**	0.000212	0.000278
**5**	0.1053	0.1065	**313**	0.000327	0.000368	**621**	0.000212	0.000278
**6**	0.070151	0.092527	**314**	0.000327	0.000368	**622**	0.000212	0.000278
**7**	0.070151	0.070993	**315**	0.000327	0.000368	**623**	0.000212	0.000278
**8**	0.068473	0.056357	**316**	0.000327	0.000368	**624**	0.000212	0.000278
**9**	0.05966	0.044666	**317**	0.000327	0.000368	**625**	0.000212	0.000278
**10**	0.044086	0.033705	**318**	0.000327	0.000368	**626**	0.000212	0.000278
**11**	0.044086	0.029807	**319**	0.000327	0.000368	**627**	0.000212	0.000278
**12**	0.039198	0.023222	**320**	0.000327	0.000368	**628**	0.000212	0.000278
**13**	0.035117	0.019988	**321**	0.000327	0.000368	**629**	0.000212	0.000278
**14**	0.035117	0.017009	**322**	0.000327	0.000368	**630**	0.000212	0.000278
**15**	0.03152	0.014344	**323**	0.000327	0.000368	**631**	0.000212	0.000278
**16**	0.026179	0.010879	**324**	0.000327	0.000368	**632**	0.000212	0.000278
**17**	0.023544	0.010879	**325**	0.000327	0.000368	**633**	0.000212	0.000278
**18**	0.019655	0.009714	**326**	0.000327	0.000368	**634**	0.000212	0.000278
**19**	0.018052	0.008152	**327**	0.000327	0.000368	**635**	0.000212	0.000278
**20**	0.018052	0.007302	**328**	0.000327	0.000368	**636**	0.000212	0.000278
**21**	0.018052	0.007016	**329**	0.000327	0.000368	**637**	0.000212	0.000278
**22**	0.018052	0.006208	**330**	0.000327	0.000368	**638**	0.000212	0.000278
**23**	0.018052	0.005823	**331**	0.000327	0.000368	**639**	0.000212	0.000278
**24**	0.016238	0.005823	**332**	0.000327	0.000368	**640**	0.000212	0.000278
**25**	0.016238	0.005371	**333**	0.000327	0.000368	**641**	0.000212	0.000278
**26**	0.016238	0.004318	**334**	0.000327	0.000368	**642**	0.000212	0.000278
**27**	0.016238	0.003677	**335**	0.000327	0.000368	**643**	0.000212	0.000278
**28**	0.016238	0.003677	**336**	0.000327	0.000368	**644**	0.000212	0.000278
**29**	0.015378	0.003677	**337**	0.000327	0.000368	**645**	0.000212	0.000278
**30**	0.012611	0.003677	**338**	0.000327	0.000368	**646**	0.000212	0.000278
**31**	0.01208	0.003677	**339**	0.000327	0.000368	**647**	0.000212	0.000278
**32**	0.009796	0.003403	**340**	0.000327	0.000368	**648**	0.000212	0.000278
**33**	0.009796	0.002723	**341**	0.000327	0.000368	**649**	0.000212	0.000278
**34**	0.009618	0.002723	**342**	0.000327	0.000368	**650**	0.000212	0.000278
**35**	0.006396	0.002645	**343**	0.000327	0.000368	**651**	0.000212	0.000278
**36**	0.006396	0.002645	**344**	0.000327	0.000368	**652**	0.000212	0.000278
**37**	0.006396	0.002645	**345**	0.000327	0.000368	**653**	0.000212	0.000278
**38**	0.006396	0.002645	**346**	0.000327	0.000368	**654**	0.000212	0.000278
**39**	0.004646	0.002246	**347**	0.000327	0.000368	**655**	0.000212	0.000278
**40**	0.004439	0.002246	**348**	0.000327	0.000368	**656**	0.000212	0.000278
**41**	0.004128	0.002061	**349**	0.000327	0.000368	**657**	0.000212	0.000278
**42**	0.003184	0.002061	**350**	0.000327	0.000368	**658**	0.000212	0.000278
**43**	0.003184	0.002047	**351**	0.000327	0.000368	**659**	0.000212	0.000278
**44**	0.002792	0.002047	**352**	0.000327	0.000368	**660**	0.000212	0.000278

**45**	0.002792	0.002025	**353**	0.000327	0.000368	**661**	0.000212	0.000278
**46**	0.002725	0.002025	**354**	0.000327	0.000368	**662**	0.000212	0.000278
**47**	0.002044	0.001417	**355**	0.000327	0.000368	**663**	0.000212	0.000278
**48**	0.002044	0.001417	**356**	0.000327	0.000368	**664**	0.000212	0.000278
**49**	0.001982	0.001417	**357**	0.000327	0.000368	**665**	0.000212	0.000278
**50**	0.001982	0.001417	**358**	0.000327	0.000368	**666**	0.000212	0.000278
**51**	0.001982	0.001417	**359**	0.000327	0.000368	**667**	0.000212	0.000278
**52**	0.001982	0.001417	**360**	0.000327	0.000368	**668**	0.000212	0.000278
**53**	0.001982	0.001417	**361**	0.000327	0.000368	**669**	0.000212	0.000278
**54**	0.001982	0.001417	**362**	0.000327	0.000368	**670**	0.000212	0.000278
**55**	0.001982	0.001417	**363**	0.000327	0.000368	**671**	0.000212	0.000278
**56**	0.001982	0.001417	**364**	0.000327	0.000368	**672**	0.000212	0.000278
**57**	0.001982	0.001417	**365**	0.000327	0.000368	**673**	0.000212	0.000278
**58**	0.001982	0.001417	**366**	0.000327	0.000368	**674**	0.000212	0.000278
**59**	0.001935	0.001417	**367**	0.000327	0.000368	**675**	0.000212	0.000278
**60**	0.001935	0.001417	**368**	0.000327	0.000368	**676**	0.000212	0.000278
**61**	0.001629	0.001417	**369**	0.000327	0.000368	**677**	0.000212	0.000278
**62**	0.001629	0.001417	**370**	0.000327	0.000368	**678**	0.000212	0.000278
**63**	0.001629	0.001417	**371**	0.000327	0.000368	**679**	0.000212	0.000278
**64**	0.001629	0.001297	**372**	0.000327	0.000368	**680**	0.000212	0.000278
**65**	0.001629	0.001297	**373**	0.000327	0.000368	**681**	0.000212	0.000278
**66**	0.001629	0.001297	**374**	0.000327	0.000368	**682**	0.000212	0.000278
**67**	0.001629	0.001297	**375**	0.000327	0.000368	**683**	0.000212	0.000261
**68**	0.001629	0.001297	**376**	0.000327	0.000368	**684**	0.000212	0.000261
**69**	0.001629	0.001297	**377**	0.000327	0.000368	**685**	0.000212	0.000261
**70**	0.001629	0.001225	**378**	0.000327	0.000368	**686**	0.000212	0.000261
**71**	0.001616	0.001225	**379**	0.000327	0.000368	**687**	0.000212	0.000261
**72**	0.001616	0.001225	**380**	0.000327	0.000368	**688**	0.000212	0.000261
**73**	0.001616	0.001128	**381**	0.000327	0.000368	**689**	0.000212	0.000261
**74**	0.001616	0.001128	**382**	0.000327	0.000368	**690**	0.000212	0.000261
**75**	0.001616	0.001128	**383**	0.000327	0.000368	**691**	0.000212	0.000261
**76**	0.001616	0.001128	**384**	0.000327	0.000368	**692**	0.000212	0.000261
**77**	0.001616	0.001128	**385**	0.000327	0.000368	**693**	0.000212	0.000261
**78**	0.001616	0.001128	**386**	0.000327	0.000368	**694**	0.000212	0.000261
**79**	0.001616	0.001128	**387**	0.000327	0.000368	**695**	0.000212	0.000261
**80**	0.001616	0.001128	**388**	0.000327	0.000368	**696**	0.000212	0.000261
**81**	0.001616	0.001128	**389**	0.000327	0.000368	**697**	0.000212	0.000261
**82**	0.001616	0.000827	**390**	0.000327	0.000368	**698**	0.000212	0.000261
**83**	0.001616	0.000827	**391**	0.000327	0.000368	**699**	0.000212	0.000261
**84**	0.001598	0.000827	**392**	0.000327	0.000368	**700**	0.000212	0.000261
**85**	0.001598	0.000827	**393**	0.000327	0.000331	**701**	0.000212	0.000261
**86**	0.001598	0.000827	**394**	0.000327	0.000331	**702**	0.000212	0.000261
**87**	0.001598	0.000827	**395**	0.000327	0.000331	**703**	0.000212	0.000261
**88**	0.001598	0.000827	**396**	0.000327	0.000331	**704**	0.000212	0.000261
**89**	0.001506	0.000827	**397**	0.000327	0.000331	**705**	0.000212	0.000261
**90**	0.001506	0.000802	**398**	0.000327	0.000331	**706**	0.000212	0.000261

**91**	0.001496	0.000802	**399**	0.000327	0.000331	**707**	0.000212	0.000261
**92**	0.001496	0.000802	**400**	0.000327	0.000331	**708**	0.000212	0.000261
**93**	0.001496	0.000802	**401**	0.000327	0.000331	**709**	0.000212	0.000261
**94**	0.001496	0.000802	**402**	0.000327	0.000331	**710**	0.000212	0.000261
**95**	0.001471	0.000802	**403**	0.000327	0.000331	**711**	0.000212	0.000257
**96**	0.001471	0.000802	**404**	0.000327	0.000331	**712**	0.000212	0.000257
**97**	0.001471	0.000802	**405**	0.000327	0.000331	**713**	0.000212	0.000257
**98**	0.001471	0.000802	**406**	0.000327	0.000331	**714**	0.000212	0.000257
**99**	0.001471	0.000802	**407**	0.000327	0.000331	**715**	0.000212	0.000257
**100**	0.001471	0.000802	**408**	0.000327	0.000331	**716**	0.000212	0.000257
**101**	0.001471	0.000802	**409**	0.000327	0.000331	**717**	0.000212	0.000257
**102**	0.001471	0.000802	**410**	0.000327	0.000331	**718**	0.000212	0.000257
**103**	0.001471	0.000802	**411**	0.000327	0.000331	**719**	0.000212	0.000257
**104**	0.001471	0.000802	**412**	0.000327	0.000331	**720**	0.000212	0.000257
**105**	0.001471	0.000802	**413**	0.000327	0.000331	**721**	0.000212	0.000257
**106**	0.001471	0.000802	**414**	0.000327	0.000331	**722**	0.000212	0.000257
**107**	0.001471	0.000802	**415**	0.000327	0.000331	**723**	0.000212	0.000257
**108**	0.001471	0.000802	**416**	0.000327	0.000331	**724**	0.000212	0.000257
**109**	0.001471	0.000802	**417**	0.000327	0.000331	**725**	0.000212	0.000257
**110**	0.001471	0.000802	**418**	0.000327	0.000331	**726**	0.000212	0.000257
**111**	0.001471	0.000802	**419**	0.000327	0.000331	**727**	0.000212	0.000257
**112**	0.001471	0.000802	**420**	0.000327	0.000331	**728**	0.000212	0.000257
**113**	0.001471	0.000802	**421**	0.000327	0.000331	**729**	0.000212	0.000257
**114**	0.001471	0.000802	**422**	0.000327	0.000331	**730**	0.000212	0.000257
**115**	0.001471	0.000802	**423**	0.000327	0.000331	**731**	0.000212	0.000257
**116**	0.001432	0.000802	**424**	0.000327	0.000331	**732**	0.000212	0.000257
**117**	0.001432	0.000802	**425**	0.000327	0.000331	**733**	0.000212	0.000257
**118**	0.001432	0.000802	**426**	0.000327	0.000331	**734**	0.000212	0.000257
**119**	0.001432	0.000802	**427**	0.000327	0.000331	**735**	0.000212	0.000257
**120**	0.001432	0.000802	**428**	0.000327	0.000331	**736**	0.000212	0.000257
**121**	0.001432	0.000802	**429**	0.000327	0.000331	**737**	0.000212	0.000257
**122**	0.001432	0.000802	**430**	0.000327	0.000331	**738**	0.000212	0.000257
**123**	0.001432	0.000802	**431**	0.000327	0.000331	**739**	0.000212	0.000257
**124**	0.001432	0.000802	**432**	0.000327	0.000331	**740**	0.000212	0.000257
**125**	0.001432	0.000802	**433**	0.000327	0.000331	**741**	0.000212	0.000257
**126**	0.001432	0.000802	**434**	0.000327	0.000331	**742**	0.000212	0.000257
**127**	0.001432	0.000802	**435**	0.000327	0.000331	**743**	0.000212	0.000257
**128**	0.001432	0.000802	**436**	0.000327	0.000331	**744**	0.000212	0.000257
**129**	0.001269	0.000802	**437**	0.000327	0.000331	**745**	0.000212	0.000257
**130**	0.001269	0.000802	**438**	0.000278	0.000331	**746**	0.000212	0.000257
**131**	0.001269	0.000802	**439**	0.000278	0.000331	**747**	0.000212	0.000257
**132**	0.001269	0.000802	**440**	0.000278	0.000331	**748**	0.000212	0.000257
**133**	0.001269	0.000802	**441**	0.000278	0.000331	**749**	0.000212	0.000257
**134**	0.001269	0.000802	**442**	0.000278	0.000331	**750**	0.000212	0.000257
**135**	0.001269	0.000802	**443**	0.000278	0.000331	**751**	0.000212	0.000257
**136**	0.001269	0.000802	**444**	0.000278	0.000331	**752**	0.000212	0.000257

**137**	0.001269	0.000802	**445**	0.000278	0.000331	**753**	0.000212	0.000257
**138**	0.001269	0.000802	**446**	0.000278	0.000331	**754**	0.000212	0.000257
**139**	0.001269	0.000802	**447**	0.000278	0.000331	**755**	0.000212	0.000257
**140**	0.001269	0.000802	**448**	0.000278	0.000331	**756**	0.000212	0.000257
**141**	0.001269	0.000802	**449**	0.000278	0.000331	**757**	0.000212	0.000257
**142**	0.001146	0.000802	**450**	0.000278	0.000331	**758**	0.000212	0.000257
**143**	0.001146	0.000802	**451**	0.000278	0.000331	**759**	0.000212	0.000257
**144**	0.001146	0.000802	**452**	0.000278	0.000331	**760**	0.000212	0.000257
**145**	0.001146	0.000691	**453**	0.000278	0.000331	**761**	0.000212	0.000257
**146**	0.001146	0.000691	**454**	0.000278	0.000331	**762**	0.000212	0.000257
**147**	0.001146	0.000691	**455**	0.000278	0.000331	**763**	0.000212	0.000257
**148**	0.001146	0.000691	**456**	0.000278	0.000331	**764**	0.000212	0.000257
**149**	0.001146	0.000691	**457**	0.000278	0.000278	**765**	0.000212	0.000257
**150**	0.001146	0.000691	**458**	0.000278	0.000278	**766**	0.000212	0.000257
**151**	0.001146	0.000691	**459**	0.000278	0.000278	**767**	0.000212	0.000257
**152**	0.001146	0.000691	**460**	0.000277	0.000278	**768**	0.000212	0.000257
**153**	0.001146	0.000691	**461**	0.000277	0.000278	**769**	0.000212	0.000257
**154**	0.001146	0.000691	**462**	0.000277	0.000278	**770**	0.000212	0.000257
**155**	0.001146	0.000691	**463**	0.000277	0.000278	**771**	0.000212	0.000257
**156**	0.001146	0.000691	**464**	0.000277	0.000278	**772**	0.000212	0.000257
**157**	0.001146	0.000691	**465**	0.000277	0.000278	**773**	0.000212	0.000257
**158**	0.001146	0.000691	**466**	0.000277	0.000278	**774**	0.000212	0.000257
**159**	0.001146	0.000691	**467**	0.000277	0.000278	**775**	0.000212	0.000257
**160**	0.000903	0.000691	**468**	0.000277	0.000278	**776**	0.000212	0.000257
**161**	0.000903	0.000691	**469**	0.000277	0.000278	**777**	0.000212	0.000222
**162**	0.000903	0.000691	**470**	0.000277	0.000278	**778**	0.000212	0.000222
**163**	0.000903	0.000691	**471**	0.000277	0.000278	**779**	0.000212	0.000222
**164**	0.000899	0.000691	**472**	0.000277	0.000278	**780**	0.000212	0.000222
**165**	0.000899	0.000691	**473**	0.000277	0.000278	**781**	0.000212	0.000222
**166**	0.000899	0.000691	**474**	0.000277	0.000278	**782**	0.000212	0.000222
**167**	0.000899	0.000543	**475**	0.000277	0.000278	**783**	0.000212	0.000222
**168**	0.000899	0.000543	**476**	0.000277	0.000278	**784**	0.000212	0.000222
**169**	0.00064	0.000543	**477**	0.000277	0.000278	**785**	0.000212	0.000222
**170**	0.00064	0.000543	**478**	0.000277	0.000278	**786**	0.000212	0.000222
**171**	0.00064	0.000543	**479**	0.000277	0.000278	**787**	0.000212	0.000222
**172**	0.00064	0.000543	**480**	0.000277	0.000278	**788**	0.000212	0.000222
**173**	0.00064	0.000543	**481**	0.000277	0.000278	**789**	0.000212	0.000222
**174**	0.00064	0.000543	**482**	0.000277	0.000278	**790**	0.000212	0.000222
**175**	0.00064	0.000543	**483**	0.000277	0.000278	**791**	0.000212	0.000222
**176**	0.00064	0.000543	**484**	0.000277	0.000278	**792**	0.000212	0.000222
**177**	0.00064	0.000543	**485**	0.000277	0.000278	**793**	0.000212	0.000222
**178**	0.00064	0.000543	**486**	0.000277	0.000278	**794**	0.000212	0.000222
**179**	0.00064	0.000543	**487**	0.000277	0.000278	**795**	0.000212	0.000222
**180**	0.00064	0.000543	**488**	0.000277	0.000278	**796**	0.000212	0.000222
**181**	0.00064	0.000543	**489**	0.000277	0.000278	**797**	0.000212	0.000222
**182**	0.00064	0.000543	**490**	0.000277	0.000278	**798**	0.000212	0.000222

**183**	0.00064	0.000543	**491**	0.000277	0.000278	**799**	0.000212	0.000222
**184**	0.00064	0.000543	**492**	0.000277	0.000278	**800**	0.000212	0.000222
**185**	0.00064	0.000543	**493**	0.000277	0.000278	**801**	0.000212	0.000222
**186**	0.000603	0.000543	**494**	0.000277	0.000278	**802**	0.000212	0.000222
**187**	0.000603	0.000543	**495**	0.000277	0.000278	**803**	0.000212	0.000222
**188**	0.000445	0.000543	**496**	0.000277	0.000278	**804**	0.000202	0.000222
**189**	0.000445	0.000543	**497**	0.000277	0.000278	**805**	0.000202	0.000222
**190**	0.000445	0.000543	**498**	0.000277	0.000278	**806**	0.000202	0.000222
**191**	0.000445	0.000543	**499**	0.000277	0.000278	**807**	0.000202	0.000222
**192**	0.000445	0.000543	**500**	0.000277	0.000278	**808**	0.000202	0.000222
**193**	0.000445	0.000543	**501**	0.000277	0.000278	**809**	0.000202	0.000222
**194**	0.000445	0.000543	**502**	0.000277	0.000278	**810**	0.000202	0.000222
**195**	0.000445	0.000543	**503**	0.000277	0.000278	**811**	0.000202	0.000222
**196**	0.000445	0.000543	**504**	0.000277	0.000278	**812**	0.000202	0.000222
**197**	0.000445	0.000543	**505**	0.000277	0.000278	**813**	0.000202	0.000222
**198**	0.000445	0.000543	**506**	0.000277	0.000278	**814**	0.000202	0.000222
**199**	0.000445	0.000543	**507**	0.000277	0.000278	**815**	0.000202	0.000222
**200**	0.000445	0.000543	**508**	0.000277	0.000278	**816**	0.000202	0.000222
**201**	0.000445	0.000543	**509**	0.000277	0.000278	**817**	0.000202	0.000222
**202**	0.000445	0.000543	**510**	0.000277	0.000278	**818**	0.000202	0.000222
**203**	0.000445	0.000543	**511**	0.000277	0.000278	**819**	0.000202	0.000222
**204**	0.000445	0.000543	**512**	0.000277	0.000278	**820**	0.000202	0.000222
**205**	0.000445	0.000543	**513**	0.000277	0.000278	**821**	0.000202	0.000222
**206**	0.000445	0.000368	**514**	0.000277	0.000278	**822**	0.000202	0.000222
**207**	0.000445	0.000368	**515**	0.000277	0.000278	**823**	0.000202	0.000222
**208**	0.000445	0.000368	**516**	0.000277	0.000278	**824**	0.000202	0.000222
**209**	0.000445	0.000368	**517**	0.000277	0.000278	**825**	0.000202	0.000222
**210**	0.000445	0.000368	**518**	0.000277	0.000278	**826**	0.000202	0.000222
**211**	0.000445	0.000368	**519**	0.000277	0.000278	**827**	0.000202	0.000222
**212**	0.000445	0.000368	**520**	0.000277	0.000278	**828**	0.000202	0.000222
**213**	0.000445	0.000368	**521**	0.000277	0.000278	**829**	0.000202	0.000222
**214**	0.000445	0.000368	**522**	0.000277	0.000278	**830**	0.000202	0.000222
**215**	0.000445	0.000368	**523**	0.000277	0.000278	**831**	0.000202	0.000222
**216**	0.000445	0.000368	**524**	0.000277	0.000278	**832**	0.000202	0.000222
**217**	0.000445	0.000368	**525**	0.000277	0.000278	**833**	0.000202	0.000222
**218**	0.000445	0.000368	**526**	0.000277	0.000278	**834**	0.000202	0.000222
**219**	0.000445	0.000368	**527**	0.000277	0.000278	**835**	0.000202	0.000222
**220**	0.000445	0.000368	**528**	0.000277	0.000278	**836**	0.000202	0.000222
**221**	0.000445	0.000368	**529**	0.000277	0.000278	**837**	0.000202	0.000222
**222**	0.000445	0.000368	**530**	0.000277	0.000278	**838**	0.000202	0.000222
**223**	0.000445	0.000368	**531**	0.000277	0.000278	**839**	0.000202	0.000222
**224**	0.000445	0.000368	**532**	0.000277	0.000278	**840**	0.000202	0.000222
**225**	0.000445	0.000368	**533**	0.000277	0.000278	**841**	0.000202	0.000222
**226**	0.000445	0.000368	**534**	0.000277	0.000278	**842**	0.000202	0.000222
**227**	0.000445	0.000368	**535**	0.000277	0.000278	**843**	0.000202	0.000222
**228**	0.000445	0.000368	**536**	0.000277	0.000278	**844**	0.000202	0.000222

**229**	0.000445	0.000368	**537**	0.000277	0.000278	**845**	0.000202	0.000222
**230**	0.000445	0.000368	**538**	0.000277	0.000278	**846**	0.000202	0.000222
**231**	0.000445	0.000368	**539**	0.000277	0.000278	**847**	0.000202	0.000222
**232**	0.000445	0.000368	**540**	0.000277	0.000278	**848**	0.000202	0.000222
**233**	0.000445	0.000368	**541**	0.000277	0.000278	**849**	0.000202	0.000222
**234**	0.000445	0.000368	**542**	0.000277	0.000278	**850**	0.000202	0.000222
**235**	0.000445	0.000368	**543**	0.000277	0.000278	**851**	0.000202	0.000222
**236**	0.000445	0.000368	**544**	0.000277	0.000278	**852**	0.000202	0.000222
**237**	0.000445	0.000368	**545**	0.000277	0.000278	**853**	0.000202	0.000222
**238**	0.000445	0.000368	**546**	0.000277	0.000278	**854**	0.000202	0.000222
**239**	0.000445	0.000368	**547**	0.000277	0.000278	**855**	0.000202	0.000222
**240**	0.000445	0.000368	**548**	0.000277	0.000278	**856**	0.000202	0.000222
**241**	0.000445	0.000368	**549**	0.000277	0.000278	**857**	0.000202	0.000222
**242**	0.000445	0.000368	**550**	0.000277	0.000278	**858**	0.000202	0.000222
**243**	0.000445	0.000368	**551**	0.000277	0.000278	**859**	0.000202	0.000222
**244**	0.000445	0.000368	**552**	0.000277	0.000278	**860**	0.000202	0.000222
**245**	0.000445	0.000368	**553**	0.000277	0.000278	**861**	0.000202	0.000222
**246**	0.000445	0.000368	**554**	0.000277	0.000278	**862**	0.000202	0.000219
**247**	0.000445	0.000368	**555**	0.000277	0.000278	**863**	0.000202	0.000219
**248**	0.000364	0.000368	**556**	0.000277	0.000278	**864**	0.000202	0.000219
**249**	0.000364	0.000368	**557**	0.000277	0.000278	**865**	0.000202	0.000219
**250**	0.000364	0.000368	**558**	0.000277	0.000278	**866**	0.000202	0.000219
**251**	0.000364	0.000368	**559**	0.000277	0.000278	**867**	0.000202	0.000219
**252**	0.000364	0.000368	**560**	0.000277	0.000278	**868**	0.000202	0.000219
**253**	0.000364	0.000368	**561**	0.000277	0.000278	**869**	0.000202	0.000219
**254**	0.000364	0.000368	**562**	0.000277	0.000278	**870**	0.000202	0.000219
**255**	0.000364	0.000368	**563**	0.000277	0.000278	**871**	0.000202	0.000219
**256**	0.000364	0.000368	**564**	0.000277	0.000278	**872**	0.000202	0.000219
**257**	0.000364	0.000368	**565**	0.000277	0.000278	**873**	0.000202	0.000219
**258**	0.000364	0.000368	**566**	0.000277	0.000278	**874**	0.000202	0.000219
**259**	0.000364	0.000368	**567**	0.000277	0.000278	**875**	0.000202	0.000219
**260**	0.000364	0.000368	**568**	0.000277	0.000278	**876**	0.000202	0.000219
**261**	0.000364	0.000368	**569**	0.000277	0.000278	**877**	0.000202	0.000219
**262**	0.000364	0.000368	**570**	0.000277	0.000278	**878**	0.000202	0.000219
**263**	0.000364	0.000368	**571**	0.000277	0.000278	**879**	0.000202	0.000219
**264**	0.000364	0.000368	**572**	0.000277	0.000278	**880**	0.000202	0.000219
**265**	0.000364	0.000368	**573**	0.000277	0.000278	**881**	0.000202	0.000219
**266**	0.000364	0.000368	**574**	0.000277	0.000278	**882**	0.000202	0.000219
**267**	0.000364	0.000368	**575**	0.000277	0.000278	**883**	0.000202	0.000219
**268**	0.000364	0.000368	**576**	0.000277	0.000278	**884**	0.000202	0.000219
**269**	0.000364	0.000368	**577**	0.000277	0.000278	**885**	0.000202	0.000219
**270**	0.000364	0.000368	**578**	0.000277	0.000278	**886**	0.000202	0.000219
**271**	0.000364	0.000368	**579**	0.000277	0.000278	**887**	0.000202	0.000219
**272**	0.000364	0.000368	**580**	0.000277	0.000278	**888**	0.000202	0.000219
**273**	0.000364	0.000368	**581**	0.000277	0.000278	**889**	0.000202	0.000219
**274**	0.000364	0.000368	**582**	0.000277	0.000278	**890**	0.000202	0.000219

**275**	0.000364	0.000368	**583**	0.000277	0.000278	**891**	0.000202	0.000219
**276**	0.000364	0.000368	**584**	0.000277	0.000278	**892**	0.000202	0.000219
**277**	0.000364	0.000368	**585**	0.000277	0.000278	**893**	0.000202	0.000219
**278**	0.000364	0.000368	**586**	0.000277	0.000278	**894**	0.000202	0.000219
**279**	0.000364	0.000368	**587**	0.000277	0.000278	**895**	0.000202	0.000219
**280**	0.000364	0.000368	**588**	0.000277	0.000278	**896**	0.000202	0.000219
**281**	0.000364	0.000368	**589**	0.000277	0.000278	**897**	0.000202	0.000219
**282**	0.000364	0.000368	**590**	0.000277	0.000278	**898**	0.000202	0.000219
**283**	0.000364	0.000368	**591**	0.000277	0.000278	**899**	0.000202	0.000219
**284**	0.000364	0.000368	**592**	0.000277	0.000278	**900**	0.000202	0.000219
**285**	0.000364	0.000368	**593**	0.000277	0.000278			
**286**	0.000364	0.000368	**594**	0.000277	0.000278			
**287**	0.000348	0.000368	**595**	0.000277	0.000278			
**288**	0.000348	0.000368	**596**	0.000277	0.000278			
**289**	0.000348	0.000368	**597**	0.000277	0.000278			
**290**	0.000348	0.000368	**598**	0.000277	0.000278			
**291**	0.000348	0.000368	**599**	0.000277	0.000278			
**292**	0.000348	0.000368	**600**	0.000277	0.000278			
**293**	0.000348	0.000368	**601**	0.000277	0.000278			
**294**	0.000348	0.000368	**602**	0.000277	0.000278			
**295**	0.000348	0.000368	**603**	0.000277	0.000278			
**296**	0.000348	0.000368	**604**	0.000277	0.000278			
**297**	0.000348	0.000368	**605**	0.000212	0.000278			
**298**	0.000348	0.000368	**606**	0.000212	0.000278			
**299**	0.000348	0.000368	**607**	0.000212	0.000278			
**300**	0.000348	0.000368	**608**	0.000212	0.000278			
**301**	0.000348	0.000368	**609**	0.000212	0.000278			
**302**	0.000348	0.000368	**610**	0.000212	0.000278			
**303**	0.000348	0.000368	**611**	0.000212	0.000278			
**304**	0.000348	0.000368	**612**	0.000212	0.000278			
**305**	0.000327	0.000368	**613**	0.000212	0.000278			
**306**	0.000327	0.000368	**614**	0.000212	0.000278			
**307**	0.000327	0.000368	**615**	0.000212	0.000278			
**308**	0.000327	0.000368	**616**	0.000212	0.000278			

Table 10. Convergence of the third damage scenario in the presence of 4% noise for first 2 and 4 modes

Iterations	2 Modes	4 Modes	Iterations	2 Modes	4 Modes	Iterations	2 Modes	4 Modes
**1**	0.20516	0.1976	**309**	0.006148	0.014742	**617**	0.005869	0.013916
**2**	0.12694	0.16302	**310**	0.006148	0.014742	**618**	0.005869	0.013916
**3**	0.11141	0.13212	**311**	0.006148	0.014742	**619**	0.005869	0.013916
**4**	0.086685	0.096999	**312**	0.006148	0.014742	**620**	0.005869	0.013916
**5**	0.070296	0.087446	**313**	0.006148	0.014742	**621**	0.005869	0.013916
**6**	0.064455	0.068492	**314**	0.006148	0.014742	**622**	0.005869	0.013916
**7**	0.054157	0.047915	**315**	0.006148	0.014742	**623**	0.005869	0.013916
**8**	0.038744	0.044209	**316**	0.006148	0.014742	**624**	0.005869	0.013916
**9**	0.038744	0.033751	**317**	0.006148	0.014742	**625**	0.005869	0.013916
**10**	0.038744	0.033312	**318**	0.006148	0.014742	**626**	0.005869	0.013916
**11**	0.038425	0.031353	**319**	0.006148	0.014742	**627**	0.005869	0.013916
**12**	0.038425	0.027079	**320**	0.006148	0.014742	**628**	0.005869	0.013916
**13**	0.031762	0.02653	**321**	0.006148	0.014742	**629**	0.005869	0.013916
**14**	0.027622	0.02433	**322**	0.006148	0.014742	**630**	0.005869	0.013916
**15**	0.026324	0.02433	**323**	0.006148	0.014742	**631**	0.005869	0.013916
**16**	0.026324	0.022286	**324**	0.006148	0.014742	**632**	0.005869	0.013916
**17**	0.026324	0.022286	**325**	0.006148	0.014742	**633**	0.005869	0.013916
**18**	0.021657	0.02221	**326**	0.006148	0.014742	**634**	0.005869	0.013916
**19**	0.021657	0.022045	**327**	0.006148	0.014742	**635**	0.005869	0.013916
**20**	0.021657	0.022045	**328**	0.006148	0.014742	**636**	0.005869	0.013916
**21**	0.021657	0.022045	**329**	0.006148	0.014742	**637**	0.005869	0.013916
**22**	0.021657	0.022045	**330**	0.006148	0.014742	**638**	0.005869	0.013916
**23**	0.021657	0.022045	**331**	0.006148	0.014742	**639**	0.005869	0.013916
**24**	0.021657	0.022045	**332**	0.006148	0.014742	**640**	0.005869	0.013916
**25**	0.021537	0.020945	**333**	0.006148	0.014742	**641**	0.005869	0.013916
**26**	0.020519	0.020945	**334**	0.006148	0.014742	**642**	0.005836	0.013916
**27**	0.020519	0.020945	**335**	0.006148	0.014742	**643**	0.005836	0.013916
**28**	0.014393	0.020945	**336**	0.006148	0.014742	**644**	0.005836	0.013916
**29**	0.014393	0.020945	**337**	0.006148	0.014742	**645**	0.005836	0.013916
**30**	0.014393	0.020945	**338**	0.006148	0.014742	**646**	0.005836	0.013916
**31**	0.014163	0.020945	**339**	0.006148	0.014742	**647**	0.005836	0.013916
**32**	0.014163	0.020945	**340**	0.006148	0.014689	**648**	0.005836	0.013916
**33**	0.014163	0.020271	**341**	0.006148	0.014689	**649**	0.005836	0.013916
**34**	0.014163	0.020271	**342**	0.006148	0.014689	**650**	0.005836	0.013916
**35**	0.013428	0.020271	**343**	0.006148	0.014689	**651**	0.005836	0.013916
**36**	0.013428	0.020271	**344**	0.006148	0.014689	**652**	0.005836	0.013916
**37**	0.013428	0.020271	**345**	0.006148	0.014689	**653**	0.005836	0.013916
**38**	0.013428	0.018552	**346**	0.006148	0.014689	**654**	0.005836	0.013916
**39**	0.013428	0.018552	**347**	0.005869	0.014689	**655**	0.005836	0.013916
**40**	0.013428	0.018552	**348**	0.005869	0.014689	**656**	0.005836	0.013916
**41**	0.013428	0.018552	**349**	0.005869	0.014689	**657**	0.005836	0.013916
**42**	0.013428	0.01807	**350**	0.005869	0.014689	**658**	0.005836	0.013916

**43**	0.013196	0.01807	**351**	0.005869	0.014689	**659**	0.005836	0.013916
**44**	0.013065	0.01807	**352**	0.005869	0.014689	**660**	0.005836	0.013916
**45**	0.013065	0.01807	**353**	0.005869	0.014689	**661**	0.005836	0.013916
**46**	0.012664	0.01807	**354**	0.005869	0.014689	**662**	0.005836	0.013916
**47**	0.012207	0.01807	**355**	0.005869	0.014689	**663**	0.005836	0.013916
**48**	0.012207	0.01807	**356**	0.005869	0.014689	**664**	0.005836	0.013916
**49**	0.012207	0.017832	**357**	0.005869	0.014689	**665**	0.005836	0.013916
**50**	0.011967	0.017832	**358**	0.005869	0.014689	**666**	0.005836	0.013916
**51**	0.011602	0.017832	**359**	0.005869	0.014689	**667**	0.005836	0.013916
**52**	0.009301	0.017832	**360**	0.005869	0.014577	**668**	0.005836	0.013916
**53**	0.009301	0.017018	**361**	0.005869	0.014577	**669**	0.005836	0.013916
**54**	0.009301	0.016804	**362**	0.005869	0.014577	**670**	0.005836	0.013845
**55**	0.009301	0.016804	**363**	0.005869	0.014577	**671**	0.005836	0.013845
**56**	0.009011	0.016804	**364**	0.005869	0.014577	**672**	0.005836	0.013845
**57**	0.009011	0.016804	**365**	0.005869	0.014577	**673**	0.005836	0.013845
**58**	0.009011	0.016804	**366**	0.005869	0.014577	**674**	0.005836	0.013845
**59**	0.009011	0.016804	**367**	0.005869	0.014577	**675**	0.005836	0.013845
**60**	0.008916	0.016804	**368**	0.005869	0.014577	**676**	0.005836	0.013845
**61**	0.00848	0.016804	**369**	0.005869	0.014577	**677**	0.005836	0.013845
**62**	0.008448	0.016804	**370**	0.005869	0.014577	**678**	0.005836	0.013845
**63**	0.008389	0.016804	**371**	0.005869	0.014577	**679**	0.005836	0.013845
**64**	0.008389	0.016804	**372**	0.005869	0.014577	**680**	0.005836	0.013845
**65**	0.007806	0.016804	**373**	0.005869	0.014577	**681**	0.005836	0.013845
**66**	0.007383	0.016804	**374**	0.005869	0.014577	**682**	0.005836	0.013845
**67**	0.007383	0.016804	**375**	0.005869	0.014577	**683**	0.005836	0.013845
**68**	0.007383	0.016804	**376**	0.005869	0.014577	**684**	0.005836	0.013845
**69**	0.007051	0.016804	**377**	0.005869	0.014577	**685**	0.005836	0.013845
**70**	0.007051	0.016804	**378**	0.005869	0.014577	**686**	0.005836	0.013845
**71**	0.007051	0.016804	**379**	0.005869	0.014577	**687**	0.005836	0.013845
**72**	0.007051	0.016804	**380**	0.005869	0.014577	**688**	0.005836	0.013845
**73**	0.007051	0.016804	**381**	0.005869	0.014577	**689**	0.005836	0.013845
**74**	0.007051	0.016804	**382**	0.005869	0.014577	**690**	0.005836	0.013845
**75**	0.007051	0.016804	**383**	0.005869	0.014577	**691**	0.005836	0.013845
**76**	0.007051	0.016804	**384**	0.005869	0.014577	**692**	0.005836	0.013845
**77**	0.007051	0.016804	**385**	0.005869	0.014577	**693**	0.005836	0.013845
**78**	0.007051	0.016804	**386**	0.005869	0.014577	**694**	0.005836	0.013845
**79**	0.006979	0.016804	**387**	0.005869	0.014577	**695**	0.005836	0.013845
**80**	0.006979	0.01642	**388**	0.005869	0.014577	**696**	0.005836	0.013845
**81**	0.006762	0.01642	**389**	0.005869	0.014577	**697**	0.005836	0.013845
**82**	0.006762	0.01642	**390**	0.005869	0.014577	**698**	0.005836	0.013845
**83**	0.006762	0.016285	**391**	0.005869	0.014577	**699**	0.005836	0.013845
**84**	0.006762	0.016285	**392**	0.005869	0.014577	**700**	0.005836	0.013845
**85**	0.006762	0.016206	**393**	0.005869	0.014577	**701**	0.005836	0.013845
**86**	0.006762	0.016206	**394**	0.005869	0.014577	**702**	0.005836	0.013845
**87**	0.00651	0.016206	**395**	0.005869	0.014577	**703**	0.005836	0.013845
**88**	0.00651	0.016206	**396**	0.005869	0.014577	**704**	0.005836	0.013845

**89**	0.00651	0.016206	**397**	0.005869	0.014577	**705**	0.005836	0.013845
**90**	0.00651	0.016206	**398**	0.005869	0.014577	**706**	0.005836	0.013845
**91**	0.00651	0.015737	**399**	0.005869	0.014577	**707**	0.005836	0.013845
**92**	0.00651	0.015737	**400**	0.005869	0.014577	**708**	0.005836	0.013845
**93**	0.00651	0.015666	**401**	0.005869	0.014577	**709**	0.005836	0.013845
**94**	0.00651	0.015666	**402**	0.005869	0.014577	**710**	0.005836	0.013845
**95**	0.00651	0.015666	**403**	0.005869	0.014577	**711**	0.005836	0.013845
**96**	0.00651	0.015666	**404**	0.005869	0.014577	**712**	0.005836	0.013845
**97**	0.00651	0.015666	**405**	0.005869	0.014577	**713**	0.005836	0.013845
**98**	0.00651	0.015666	**406**	0.005869	0.014577	**714**	0.005836	0.013845
**99**	0.00651	0.015666	**407**	0.005869	0.014577	**715**	0.005836	0.013845
**100**	0.00651	0.015666	**408**	0.005869	0.014577	**716**	0.005836	0.013845
**101**	0.00651	0.015666	**409**	0.005869	0.014577	**717**	0.005836	0.013845
**102**	0.00651	0.015666	**410**	0.005869	0.014577	**718**	0.005836	0.013845
**103**	0.00651	0.015666	**411**	0.005869	0.014577	**719**	0.005836	0.013845
**104**	0.00651	0.015666	**412**	0.005869	0.014577	**720**	0.005836	0.013845
**105**	0.00651	0.015666	**413**	0.005869	0.014577	**721**	0.005836	0.013845
**106**	0.00651	0.015666	**414**	0.005869	0.014577	**722**	0.005836	0.013845
**107**	0.00651	0.015666	**415**	0.005869	0.014577	**723**	0.005836	0.013845
**108**	0.00651	0.015666	**416**	0.005869	0.014577	**724**	0.005836	0.013845
**109**	0.00651	0.015666	**417**	0.005869	0.014577	**725**	0.005836	0.013682
**110**	0.00651	0.015666	**418**	0.005869	0.014577	**726**	0.005836	0.013682
**111**	0.00651	0.015666	**419**	0.005869	0.014577	**727**	0.005836	0.013682
**112**	0.00651	0.015666	**420**	0.005869	0.014577	**728**	0.005836	0.013682
**113**	0.00651	0.015666	**421**	0.005869	0.014577	**729**	0.005836	0.013682
**114**	0.00651	0.015666	**422**	0.005869	0.014577	**730**	0.005836	0.013682
**115**	0.006499	0.015486	**423**	0.005869	0.014577	**731**	0.005836	0.013682
**116**	0.006499	0.015486	**424**	0.005869	0.014577	**732**	0.005836	0.013682
**117**	0.006499	0.015486	**425**	0.005869	0.014577	**733**	0.005836	0.013682
**118**	0.006499	0.015486	**426**	0.005869	0.014577	**734**	0.005836	0.013682
**119**	0.006499	0.015486	**427**	0.005869	0.014577	**735**	0.005836	0.013682
**120**	0.006499	0.015486	**428**	0.005869	0.014577	**736**	0.005836	0.013682
**121**	0.006499	0.015486	**429**	0.005869	0.014577	**737**	0.005836	0.013682
**122**	0.006374	0.015486	**430**	0.005869	0.014577	**738**	0.005836	0.013682
**123**	0.006374	0.015486	**431**	0.005869	0.014577	**739**	0.005836	0.013682
**124**	0.006374	0.015486	**432**	0.005869	0.014577	**740**	0.005836	0.013682
**125**	0.006374	0.015486	**433**	0.005869	0.014577	**741**	0.005836	0.013682
**126**	0.006374	0.015486	**434**	0.005869	0.014577	**742**	0.005836	0.013682
**127**	0.006374	0.015486	**435**	0.005869	0.014577	**743**	0.005836	0.013682
**128**	0.006374	0.015486	**436**	0.005869	0.014577	**744**	0.005836	0.013682
**129**	0.006374	0.015486	**437**	0.005869	0.014577	**745**	0.005836	0.013682
**130**	0.006374	0.015486	**438**	0.005869	0.014577	**746**	0.005836	0.013682
**131**	0.006374	0.015486	**439**	0.005869	0.014449	**747**	0.005836	0.013682
**132**	0.006374	0.015486	**440**	0.005869	0.014449	**748**	0.005836	0.013682
**133**	0.006374	0.015486	**441**	0.005869	0.014449	**749**	0.005836	0.013682
**134**	0.006374	0.015486	**442**	0.005869	0.014449	**750**	0.005836	0.013682

**135**	0.006374	0.015486	**443**	0.005869	0.014449	**751**	0.005836	0.013682
**136**	0.006374	0.015486	**444**	0.005869	0.014449	**752**	0.005836	0.013682
**137**	0.006374	0.015486	**445**	0.005869	0.014449	**753**	0.005836	0.013682
**138**	0.006374	0.015486	**446**	0.005869	0.014449	**754**	0.005836	0.013682
**139**	0.006374	0.015486	**447**	0.005869	0.014449	**755**	0.005836	0.013682
**140**	0.006374	0.015486	**448**	0.005869	0.014449	**756**	0.005836	0.013682
**141**	0.006268	0.015486	**449**	0.005869	0.014449	**757**	0.005836	0.013682
**142**	0.006268	0.015486	**450**	0.005869	0.014449	**758**	0.005836	0.013682
**143**	0.006268	0.015486	**451**	0.005869	0.014449	**759**	0.005836	0.013682
**144**	0.006268	0.015486	**452**	0.005869	0.014449	**760**	0.005836	0.013682
**145**	0.006268	0.015486	**453**	0.005869	0.014449	**761**	0.005836	0.013682
**146**	0.006268	0.015486	**454**	0.005869	0.014133	**762**	0.005836	0.013682
**147**	0.006268	0.015486	**455**	0.005869	0.014133	**763**	0.005836	0.013682
**148**	0.006268	0.015486	**456**	0.005869	0.014133	**764**	0.005836	0.013682
**149**	0.006268	0.015486	**457**	0.005869	0.014133	**765**	0.005836	0.013682
**150**	0.006268	0.015486	**458**	0.005869	0.014133	**766**	0.005836	0.013682
**151**	0.006268	0.015486	**459**	0.005869	0.014133	**767**	0.005836	0.013682
**152**	0.006268	0.015486	**460**	0.005869	0.014133	**768**	0.005836	0.013682
**153**	0.006268	0.015486	**461**	0.005869	0.014133	**769**	0.005836	0.013682
**154**	0.006268	0.015486	**462**	0.005869	0.014133	**770**	0.005836	0.013682
**155**	0.006268	0.015486	**463**	0.005869	0.014133	**771**	0.005836	0.013682
**156**	0.006268	0.015486	**464**	0.005869	0.014133	**772**	0.005836	0.013682
**157**	0.006268	0.015486	**465**	0.005869	0.014133	**773**	0.005836	0.013682
**158**	0.006268	0.015486	**466**	0.005869	0.014133	**774**	0.005836	0.013682
**159**	0.006268	0.015486	**467**	0.005869	0.014133	**775**	0.005836	0.013682
**160**	0.006268	0.015486	**468**	0.005869	0.014133	**776**	0.005836	0.013682
**161**	0.006268	0.015486	**469**	0.005869	0.014133	**777**	0.005836	0.013682
**162**	0.006268	0.014742	**470**	0.005869	0.014133	**778**	0.005836	0.013682
**163**	0.006268	0.014742	**471**	0.005869	0.014133	**779**	0.005836	0.013682
**164**	0.006268	0.014742	**472**	0.005869	0.014133	**780**	0.005836	0.013682
**165**	0.006268	0.014742	**473**	0.005869	0.014133	**781**	0.005836	0.013682
**166**	0.006268	0.014742	**474**	0.005869	0.014133	**782**	0.005836	0.013682
**167**	0.006268	0.014742	**475**	0.005869	0.014133	**783**	0.005836	0.013682
**168**	0.006268	0.014742	**476**	0.005869	0.014133	**784**	0.005836	0.013682
**169**	0.006268	0.014742	**477**	0.005869	0.014133	**785**	0.005836	0.013682
**170**	0.006268	0.014742	**478**	0.005869	0.014133	**786**	0.005836	0.013682
**171**	0.006268	0.014742	**479**	0.005869	0.014133	**787**	0.005836	0.013682
**172**	0.006268	0.014742	**480**	0.005869	0.014133	**788**	0.005836	0.013682
**173**	0.006268	0.014742	**481**	0.005869	0.014133	**789**	0.005836	0.013682
**174**	0.006268	0.014742	**482**	0.005869	0.014133	**790**	0.005836	0.013682
**175**	0.006268	0.014742	**483**	0.005869	0.014133	**791**	0.005836	0.013682
**176**	0.006268	0.014742	**484**	0.005869	0.014133	**792**	0.005836	0.013682
**177**	0.006268	0.014742	**485**	0.005869	0.014133	**793**	0.005836	0.013682
**178**	0.006268	0.014742	**486**	0.005869	0.014133	**794**	0.005836	0.013682
**179**	0.006268	0.014742	**487**	0.005869	0.014133	**795**	0.005836	0.013682
**180**	0.006268	0.014742	**488**	0.005869	0.014133	**796**	0.005836	0.013682

**181**	0.006268	0.014742	**489**	0.005869	0.014133	**797**	0.005836	0.013682
**182**	0.006268	0.014742	**490**	0.005869	0.014133	**798**	0.005836	0.013682
**183**	0.006268	0.014742	**491**	0.005869	0.014133	**799**	0.005836	0.013682
**184**	0.006268	0.014742	**492**	0.005869	0.014133	**800**	0.005836	0.013682
**185**	0.006268	0.014742	**493**	0.005869	0.014133	**801**	0.005836	0.013682
**186**	0.006268	0.014742	**494**	0.005869	0.014133	**802**	0.005836	0.013682
**187**	0.006268	0.014742	**495**	0.005869	0.014133	**803**	0.005836	0.013682
**188**	0.006268	0.014742	**496**	0.005869	0.014133	**804**	0.005836	0.013682
**189**	0.006268	0.014742	**497**	0.005869	0.014133	**805**	0.005836	0.013682
**190**	0.006268	0.014742	**498**	0.005869	0.014133	**806**	0.005836	0.013682
**191**	0.006268	0.014742	**499**	0.005869	0.014133	**807**	0.005836	0.013682
**192**	0.006268	0.014742	**500**	0.005869	0.014133	**808**	0.005836	0.013682
**193**	0.006268	0.014742	**501**	0.005869	0.014133	**809**	0.005836	0.013682
**194**	0.006268	0.014742	**502**	0.005869	0.014133	**810**	0.005836	0.013682
**195**	0.006268	0.014742	**503**	0.005869	0.014133	**811**	0.005836	0.013682
**196**	0.006268	0.014742	**504**	0.005869	0.014133	**812**	0.005836	0.013682
**197**	0.006268	0.014742	**505**	0.005869	0.014133	**813**	0.005836	0.013682
**198**	0.006268	0.014742	**506**	0.005869	0.014133	**814**	0.005836	0.013682
**199**	0.006268	0.014742	**507**	0.005869	0.014133	**815**	0.005836	0.013682
**200**	0.006268	0.014742	**508**	0.005869	0.014133	**816**	0.005836	0.013682
**201**	0.006268	0.014742	**509**	0.005869	0.014133	**817**	0.005836	0.013682
**202**	0.006268	0.014742	**510**	0.005869	0.014133	**818**	0.005836	0.013682
**203**	0.006268	0.014742	**511**	0.005869	0.014133	**819**	0.005836	0.013682
**204**	0.006268	0.014742	**512**	0.005869	0.014133	**820**	0.005836	0.013682
**205**	0.006268	0.014742	**513**	0.005869	0.014133	**821**	0.005836	0.013682
**206**	0.006268	0.014742	**514**	0.005869	0.014133	**822**	0.005836	0.013682
**207**	0.006268	0.014742	**515**	0.005869	0.014133	**823**	0.005836	0.013682
**208**	0.006268	0.014742	**516**	0.005869	0.014133	**824**	0.005836	0.013682
**209**	0.006268	0.014742	**517**	0.005869	0.014133	**825**	0.005836	0.013682
**210**	0.006268	0.014742	**518**	0.005869	0.014133	**826**	0.005836	0.013682
**211**	0.006268	0.014742	**519**	0.005869	0.014133	**827**	0.005836	0.013682
**212**	0.006203	0.014742	**520**	0.005869	0.014133	**828**	0.005836	0.013682
**213**	0.006203	0.014742	**521**	0.005869	0.014133	**829**	0.005836	0.013682
**214**	0.006203	0.014742	**522**	0.005869	0.014133	**830**	0.005836	0.013682
**215**	0.006156	0.014742	**523**	0.005869	0.014133	**831**	0.005836	0.013682
**216**	0.006156	0.014742	**524**	0.005869	0.014133	**832**	0.005836	0.013682
**217**	0.006156	0.014742	**525**	0.005869	0.013967	**833**	0.005836	0.013682
**218**	0.006156	0.014742	**526**	0.005869	0.013967	**834**	0.005836	0.013682
**219**	0.006148	0.014742	**527**	0.005869	0.013967	**835**	0.005836	0.013682
**220**	0.006148	0.014742	**528**	0.005869	0.013967	**836**	0.005836	0.013682
**221**	0.006148	0.014742	**529**	0.005869	0.013967	**837**	0.005836	0.013682
**222**	0.006148	0.014742	**530**	0.005869	0.013967	**838**	0.005836	0.013682
**223**	0.006148	0.014742	**531**	0.005869	0.013967	**839**	0.005836	0.013682
**224**	0.006148	0.014742	**532**	0.005869	0.013967	**840**	0.005836	0.013682
**225**	0.006148	0.014742	**533**	0.005869	0.013967	**841**	0.005836	0.013682
**226**	0.006148	0.014742	**534**	0.005869	0.013967	**842**	0.005836	0.013682

**227**	0.006148	0.014742	**535**	0.005869	0.013967	**843**	0.005836	0.013682
**228**	0.006148	0.014742	**536**	0.005869	0.013967	**844**	0.005836	0.013682
**229**	0.006148	0.014742	**537**	0.005869	0.013967	**845**	0.005836	0.013682
**230**	0.006148	0.014742	**538**	0.005869	0.013967	**846**	0.005836	0.013682
**231**	0.006148	0.014742	**539**	0.005869	0.013967	**847**	0.005836	0.013682
**232**	0.006148	0.014742	**540**	0.005869	0.013967	**848**	0.005836	0.013682
**233**	0.006148	0.014742	**541**	0.005869	0.013967	**849**	0.005836	0.013682
**234**	0.006148	0.014742	**542**	0.005869	0.013954	**850**	0.005836	0.013682
**235**	0.006148	0.014742	**543**	0.005869	0.013954	**851**	0.005836	0.013682
**236**	0.006148	0.014742	**544**	0.005869	0.013954	**852**	0.005836	0.013682
**237**	0.006148	0.014742	**545**	0.005869	0.013954	**853**	0.005836	0.013682
**238**	0.006148	0.014742	**546**	0.005869	0.013954	**854**	0.005836	0.013682
**239**	0.006148	0.014742	**547**	0.005869	0.013954	**855**	0.005836	0.013682
**240**	0.006148	0.014742	**548**	0.005869	0.013954	**856**	0.005836	0.013682
**241**	0.006148	0.014742	**549**	0.005869	0.013954	**857**	0.005836	0.013682
**242**	0.006148	0.014742	**550**	0.005869	0.013954	**858**	0.005836	0.013682
**243**	0.006148	0.014742	**551**	0.005869	0.013954	**859**	0.005836	0.013682
**244**	0.006148	0.014742	**552**	0.005869	0.013954	**860**	0.005836	0.013682
**245**	0.006148	0.014742	**553**	0.005869	0.013954	**861**	0.005836	0.013682
**246**	0.006148	0.014742	**554**	0.005869	0.013916	**862**	0.005836	0.013682
**247**	0.006148	0.014742	**555**	0.005869	0.013916	**863**	0.005836	0.013682
**248**	0.006148	0.014742	**556**	0.005869	0.013916	**864**	0.005836	0.013682
**249**	0.006148	0.014742	**557**	0.005869	0.013916	**865**	0.005836	0.013682
**250**	0.006148	0.014742	**558**	0.005869	0.013916	**866**	0.005836	0.013682
**251**	0.006148	0.014742	**559**	0.005869	0.013916	**867**	0.005836	0.013682
**252**	0.006148	0.014742	**560**	0.005869	0.013916	**868**	0.005836	0.013682
**253**	0.006148	0.014742	**561**	0.005869	0.013916	**869**	0.005836	0.013682
**254**	0.006148	0.014742	**562**	0.005869	0.013916	**870**	0.005836	0.013682
**255**	0.006148	0.014742	**563**	0.005869	0.013916	**871**	0.005836	0.013682
**256**	0.006148	0.014742	**564**	0.005869	0.013916	**872**	0.005836	0.013682
**257**	0.006148	0.014742	**565**	0.005869	0.013916	**873**	0.005836	0.013682
**258**	0.006148	0.014742	**566**	0.005869	0.013916	**874**	0.005836	0.013682
**259**	0.006148	0.014742	**567**	0.005869	0.013916	**875**	0.005836	0.013682
**260**	0.006148	0.014742	**568**	0.005869	0.013916	**876**	0.005836	0.013682
**261**	0.006148	0.014742	**569**	0.005869	0.013916	**877**	0.005836	0.013682
**262**	0.006148	0.014742	**570**	0.005869	0.013916	**878**	0.005836	0.013682
**263**	0.006148	0.014742	**571**	0.005869	0.013916	**879**	0.005805	0.013682
**264**	0.006148	0.014742	**572**	0.005869	0.013916	**880**	0.005805	0.013682
**265**	0.006148	0.014742	**573**	0.005869	0.013916	**881**	0.005805	0.013682
**266**	0.006148	0.014742	**574**	0.005869	0.013916	**882**	0.005805	0.013682
**267**	0.006148	0.014742	**575**	0.005869	0.013916	**883**	0.005805	0.013682
**268**	0.006148	0.014742	**576**	0.005869	0.013916	**884**	0.005805	0.013682
**269**	0.006148	0.014742	**577**	0.005869	0.013916	**885**	0.005805	0.013682
**270**	0.006148	0.014742	**578**	0.005869	0.013916	**886**	0.005805	0.013682
**271**	0.006148	0.014742	**579**	0.005869	0.013916	**887**	0.005805	0.013682
**272**	0.006148	0.014742	**580**	0.005869	0.013916	**888**	0.005805	0.013682

**273**	0.006148	0.014742	**581**	0.005869	0.013916	**889**	0.005805	0.013682
**274**	0.006148	0.014742	**582**	0.005869	0.013916	**890**	0.005805	0.013682
**275**	0.006148	0.014742	**583**	0.005869	0.013916	**891**	0.005805	0.013682
**276**	0.006148	0.014742	**584**	0.005869	0.013916	**892**	0.005805	0.013682
**277**	0.006148	0.014742	**585**	0.005869	0.013916	**893**	0.005805	0.013682
**278**	0.006148	0.014742	**586**	0.005869	0.013916	**894**	0.005805	0.013672
**279**	0.006148	0.014742	**587**	0.005869	0.013916	**895**	0.005805	0.013672
**280**	0.006148	0.014742	**588**	0.005869	0.013916	**896**	0.005805	0.013672
**281**	0.006148	0.014742	**589**	0.005869	0.013916	**897**	0.005805	0.013672
**282**	0.006148	0.014742	**590**	0.005869	0.013916	**898**	0.005805	0.013672
**283**	0.006148	0.014742	**591**	0.005869	0.013916	**899**	0.005805	0.013672
**284**	0.006148	0.014742	**592**	0.005869	0.013916	**900**	0.005805	0.013672
**285**	0.006148	0.014742	**593**	0.005869	0.013916			
**286**	0.006148	0.014742	**594**	0.005869	0.013916			
**287**	0.006148	0.014742	**595**	0.005869	0.013916			
**288**	0.006148	0.014742	**596**	0.005869	0.013916			
**289**	0.006148	0.014742	**597**	0.005869	0.013916			
**290**	0.006148	0.014742	**598**	0.005869	0.013916			
**291**	0.006148	0.014742	**599**	0.005869	0.013916			
**292**	0.006148	0.014742	**600**	0.005869	0.013916			
**293**	0.006148	0.014742	**601**	0.005869	0.013916			
**294**	0.006148	0.014742	**602**	0.005869	0.013916			
**295**	0.006148	0.014742	**603**	0.005869	0.013916			
**296**	0.006148	0.014742	**604**	0.005869	0.013916			
**297**	0.006148	0.014742	**605**	0.005869	0.013916			
**298**	0.006148	0.014742	**606**	0.005869	0.013916			
**299**	0.006148	0.014742	**607**	0.005869	0.013916			
**300**	0.006148	0.014742	**608**	0.005869	0.013916			
**301**	0.006148	0.014742	**609**	0.005869	0.013916			
**302**	0.006148	0.014742	**610**	0.005869	0.013916			
**303**	0.006148	0.014742	**611**	0.005869	0.013916			
**304**	0.006148	0.014742	**612**	0.005869	0.013916			
**305**	0.006148	0.014742	**613**	0.005869	0.013916			
**306**	0.006148	0.014742	**614**	0.005869	0.013916			
**307**	0.006148	0.014742	**615**	0.005869	0.013916			
**308**	0.006148	0.014742	**616**	0.005869	0.013916			

Table 11. Damage identification results of 23-member frame in the first damage scenario using the information of the first mode

Member	Actual Damage	1 Mode 0% Noise	1 Mode 5% Noise
**1**	0	0	0
**2**	0	0	0.38
**3**	0	0	0.92
**4**	5	4.89	4.4
**5**	0	0	0.29
**6**	0	0	0.12
**7**	0	0	0
**8**	0	0	0
**9**	0	0	0.34
**10**	0	0.15	0.67
**11**	0	0	0.74
**12**	0	0	0.28
**13**	0	0.11	0.4
**14**	0	0	0.18
**15**	0	0	0
**16**	0	0	0.21
**17**	0	0	0
**18**	10	9.92	9.89
**19**	0	0	0
**20**	0	0.13	0
**21**	0	0.11	0.52
**22**	0	0	0.23
**23**	0	0	0.42

Table 12. Damage identification results of 23-member frame in the second damage scenario using the information of the first mode

Member	Actual Damage	1 Mode 0% Noise	1 Mode 5% Noise
**1**	0	0.1	0
**2**	15	14.58	14.1
**3**	0	0	0
**4**	0	0	0
**5**	0	0	0
**6**	0	0	0
**7**	0	0	0
**8**	0	0	0
**9**	10	9.93	9.77
**10**	0	0	0
**10**	0	0.23	0.18
**12**	0	0.16	0
**13**	0	0	0
**14**	0	0	0.12
**15**	0	0	0
**16**	0	0	0
**17**	0	0	0
**18**	0	0.11	0
**19**	20	19.97	19.97
**20**	0	0	0
**21**	0	0	0.12
**22**	0	0	0.24
**23**	0	0	0.19

Table 13. Damage identification results of 23-member frame in the third damage scenario using the information of the first mode

Member	Actual Damage	1 Mode 0% Noise	1 Mode 5% Noise
**1**	0	0.8	0
**2**	10	9.74	10.4
**3**	0	0.1	2
**4**	0	0	0
**5**	0	0	0.3
**6**	0	0	0.6
**7**	0	0	0.2
**8**	0	0	0
**9**	0	0	0.01
**10**	0	0	0
**11**	0	0	0.6
**12**	20	19.71	19.97
**13**	0	0	0
**14**	0	0	0
**15**	0	0	0
**16**	0	0.2	0.2
**17**	15	15	15.77
**18**	0	0	0
**19**	0	0	0.1
**20**	0	0	0
**21**	0	0	0.3
**22**	0	0	0
**23**	0	0	0

Table 14. Damage identification results of 23-member frame in the first damage scenario using the information of the first 3 modes

Member	Actual Damage	3 Modes 0% Noise	3 Modes 5% Noise
**1**	0	0	0.76
**2**	0	0	0.37
**3**	0	0	1.57
**4**	5	4.95	5.7
**5**	0	0	0
**6**	0	0	0.13
**7**	0	0	0.41
**8**	0	0	0
**9**	0	0	0.29
**10**	0	0	0
**11**	0	0	0
**12**	0	0	1.3
**13**	0	0.1	0
**14**	0	0	0.86
**15**	0	0	0.25
**16**	0	0	0
**17**	0	0	0.46
**18**	10	9.91	10.93
**19**	0	0	0
**20**	0	0	1.56
**21**	0	0	0.72
**22**	0	0	0
**23**	0	0.1	0

Table 15. Damage identification results of 23-member frame in the second damage scenario using the information of the first 3 modes

Member	Actual Damage	3 Modes 0% Noise	3 Modes 5% Noise
**1**	0	0	0
**2**	15	14.64	16.69
**3**	0	0.12	0.6
**4**	0	0.13	0.2
**5**	0	0.18	0
**6**	0	0	0
**7**	0	0	0
**8**	0	0	3
**9**	10	9.78	11.35
**10**	0	0	0.36
**10**	0	0	0.13
**12**	0	0.1	0.49
**13**	0	0	0
**14**	0	0	0.11
**15**	0	0.33	0.38
**16**	0	0.21	0
**17**	0	0	0.91
**18**	0	0.39	0
**19**	20	19.93	19.67
**20**	0	0	0
**21**	0	0	0.65
**22**	0	0	0.13
**23**	0	0	0

Table 16. Damage identification results of 23-member frame in the third damage scenario using the information of the first 3 modes

Member	Actual Damage	3 Modes 0% Noise	3 Modes 5% Noise
**1**	15	14.69	14.16
**2**	0	0	0
**3**	0	0	0
**4**	0	0	0.81
**5**	0	0	0
**6**	0	0	0.58
**7**	0	0.24	0.11
**8**	20	19.7	16.78
**9**	0	0	0
**10**	0	0	1.76
**11**	0	0.21	0.84
**12**	0	0	0.24
**13**	0	0.16	0.25
**14**	0	0.11	0
**15**	0	0.27	0
**16**	25	25.18	24.43
**17**	0	0.15	0
**18**	0	0	0.27
**19**	0	0	0
**20**	0	0.26	0.33
**21**	0	0	1.23
**22**	10	9.94	9.91
**23**	0	0	0

Table 17. Convergence of the first damage scenario in the absence of noise for first 1 and 3 modes

Iterations	1 Mode	3 Modes	Iterations	1 Modes	3 Modes	Iterations	1 Modes	3 Modes
**1**	0.30977	0.32255	**319**	0.002315	0.000823	**637**	0.001391	0.000812
**2**	0.27761	0.24899	**320**	0.002315	0.000823	**638**	0.001391	0.000812
**3**	0.21998	0.21101	**321**	0.002315	0.000823	**639**	0.001391	0.000812
**4**	0.17215	0.17163	**322**	0.002315	0.000823	**640**	0.001391	0.000812
**5**	0.13388	0.12258	**323**	0.002315	0.000823	**641**	0.001391	0.000812
**6**	0.10791	0.095719	**324**	0.002315	0.000823	**642**	0.001391	0.000812
**7**	0.091478	0.080966	**325**	0.002315	0.000823	**643**	0.001391	0.000812
**8**	0.069395	0.061528	**326**	0.002315	0.000823	**644**	0.001391	0.000812
**9**	0.062995	0.056798	**327**	0.002315	0.000823	**645**	0.001391	0.000812
**10**	0.062995	0.050717	**328**	0.002315	0.000823	**646**	0.001391	0.000812
**11**	0.043124	0.0408	**329**	0.002315	0.000823	**647**	0.001391	0.000812
**12**	0.043124	0.0408	**330**	0.002315	0.000823	**648**	0.001391	0.000812
**13**	0.026682	0.038514	**331**	0.002315	0.000823	**649**	0.001391	0.000812
**14**	0.026682	0.038514	**332**	0.002315	0.000823	**650**	0.001391	0.000812
**15**	0.026682	0.033374	**333**	0.002315	0.000823	**651**	0.001391	0.000812
**16**	0.022147	0.022911	**334**	0.002315	0.000823	**652**	0.001391	0.000812
**17**	0.022147	0.022911	**335**	0.002315	0.000823	**653**	0.001391	0.000812
**18**	0.020445	0.022911	**336**	0.002315	0.000823	**654**	0.001391	0.000812
**19**	0.020445	0.022911	**337**	0.002315	0.000823	**655**	0.001391	0.000812
**20**	0.014083	0.021734	**338**	0.001994	0.000823	**656**	0.001391	0.000812
**21**	0.014083	0.01875	**339**	0.001994	0.000823	**657**	0.001391	0.000812
**22**	0.014083	0.01875	**340**	0.001994	0.000823	**658**	0.001391	0.000812
**23**	0.013633	0.01875	**341**	0.001994	0.000823	**659**	0.001391	0.000812
**24**	0.010942	0.01875	**342**	0.001994	0.000823	**660**	0.001391	0.000812
**25**	0.009263	0.01875	**343**	0.001994	0.000823	**661**	0.001391	0.000812
**26**	0.009263	0.016768	**344**	0.001931	0.000823	**662**	0.001391	0.000812
**27**	0.009263	0.016768	**345**	0.001931	0.000823	**663**	0.001391	0.000812
**28**	0.009263	0.016768	**346**	0.001931	0.000823	**664**	0.001391	0.000812
**29**	0.007289	0.016768	**347**	0.001931	0.000823	**665**	0.001391	0.000812
**30**	0.007289	0.016768	**348**	0.001931	0.000823	**666**	0.001391	0.000812
**31**	0.007289	0.016768	**349**	0.001931	0.000823	**667**	0.001391	0.000812
**32**	0.007289	0.016768	**350**	0.001931	0.000823	**668**	0.001391	0.000812
**33**	0.007289	0.016768	**351**	0.001931	0.000823	**669**	0.001391	0.000812
**34**	0.007289	0.016768	**352**	0.001931	0.000823	**670**	0.001391	0.000812
**35**	0.007289	0.016768	**353**	0.001931	0.000823	**671**	0.001391	0.000812
**36**	0.007289	0.016768	**354**	0.001931	0.000823	**672**	0.001391	0.000812
**37**	0.007289	0.015571	**355**	0.001931	0.000823	**673**	0.001391	0.000812
**38**	0.007289	0.015571	**356**	0.001931	0.000823	**674**	0.001391	0.000812
**39**	0.007289	0.015571	**357**	0.001931	0.000823	**675**	0.001391	0.000812
**40**	0.007289	0.015571	**358**	0.001931	0.000823	**676**	0.001391	0.000812
**41**	0.007289	0.015571	**359**	0.001931	0.000823	**677**	0.001391	0.000812
**42**	0.007289	0.015571	**360**	0.001931	0.000823	**678**	0.001391	0.000812
**43**	0.007289	0.015571	**361**	0.001931	0.000823	**679**	0.001391	0.000812
**44**	0.007289	0.014977	**362**	0.001931	0.000823	**680**	0.001391	0.000812

**45**	0.007289	0.014977	**363**	0.001931	0.000823	**681**	0.001391	0.000812
**46**	0.007289	0.014404	**364**	0.001931	0.000823	**682**	0.001391	0.000812
**47**	0.007289	0.014404	**365**	0.001931	0.000823	**683**	0.001391	0.000812
**48**	0.007289	0.014404	**366**	0.001931	0.000823	**684**	0.001391	0.000812
**49**	0.006746	0.014404	**367**	0.001931	0.000823	**685**	0.001391	0.000812
**50**	0.006746	0.014404	**368**	0.001931	0.000823	**686**	0.001391	0.000812
**51**	0.006746	0.014404	**369**	0.001931	0.000823	**687**	0.001391	0.000812
**52**	0.006746	0.014404	**370**	0.001931	0.000823	**688**	0.001391	0.000812
**53**	0.006746	0.014404	**371**	0.001931	0.000823	**689**	0.001391	0.000812
**54**	0.006746	0.014404	**372**	0.001931	0.000823	**690**	0.001391	0.000812
**55**	0.006746	0.014404	**373**	0.001931	0.000823	**691**	0.001391	0.000812
**56**	0.006746	0.014404	**374**	0.001931	0.000823	**692**	0.001391	0.000812
**57**	0.006746	0.014404	**375**	0.001931	0.000823	**693**	0.001391	0.000812
**58**	0.006746	0.014404	**376**	0.001931	0.000823	**694**	0.001391	0.000812
**59**	0.006746	0.014404	**377**	0.001931	0.000823	**695**	0.001391	0.000812
**60**	0.006746	0.013594	**378**	0.001931	0.000823	**696**	0.001391	0.000812
**61**	0.006746	0.012622	**379**	0.001931	0.000823	**697**	0.001391	0.000812
**62**	0.006746	0.012622	**380**	0.001931	0.000823	**698**	0.001391	0.000653
**63**	0.006746	0.012622	**381**	0.001931	0.000823	**699**	0.001391	0.000653
**64**	0.006746	0.012622	**382**	0.001931	0.000823	**700**	0.001391	0.000653
**65**	0.006746	0.012622	**383**	0.001931	0.000823	**701**	0.001391	0.000653
**66**	0.006746	0.012622	**384**	0.001931	0.000823	**702**	0.001391	0.000653
**67**	0.006746	0.012622	**385**	0.001931	0.000823	**703**	0.001391	0.000653
**68**	0.006746	0.012622	**386**	0.001931	0.000823	**704**	0.001391	0.000653
**69**	0.006746	0.012622	**387**	0.001931	0.000823	**705**	0.001391	0.000653
**70**	0.006318	0.012006	**388**	0.001931	0.000823	**706**	0.001391	0.000653
**71**	0.006318	0.012006	**389**	0.001931	0.000823	**707**	0.001391	0.000653
**72**	0.006318	0.012006	**390**	0.001931	0.000823	**708**	0.001249	0.000653
**73**	0.006318	0.012006	**391**	0.001931	0.000823	**709**	0.001249	0.000653
**74**	0.006318	0.011249	**392**	0.001931	0.000823	**710**	0.001249	0.000653
**75**	0.006318	0.011045	**393**	0.001931	0.000823	**711**	0.001249	0.000653
**76**	0.006318	0.010305	**394**	0.001931	0.000823	**712**	0.001249	0.000653
**77**	0.006318	0.010305	**395**	0.001931	0.000823	**713**	0.001249	0.000653
**78**	0.006318	0.009178	**396**	0.001931	0.000823	**714**	0.001249	0.000653
**79**	0.006318	0.009178	**397**	0.001931	0.000823	**715**	0.001249	0.000653
**80**	0.006318	0.008628	**398**	0.001931	0.000823	**716**	0.001249	0.000653
**81**	0.006318	0.008628	**399**	0.001931	0.000823	**717**	0.001249	0.000653
**82**	0.006318	0.006482	**400**	0.001931	0.000823	**718**	0.001249	0.000653
**83**	0.006318	0.006195	**401**	0.001931	0.000823	**719**	0.001249	0.000653
**84**	0.006318	0.004389	**402**	0.001931	0.000823	**720**	0.001249	0.000653
**85**	0.006219	0.004389	**403**	0.001931	0.000823	**721**	0.001249	0.000653
**86**	0.006219	0.004389	**404**	0.001931	0.000823	**722**	0.001249	0.000653
**87**	0.006219	0.004389	**405**	0.001931	0.000823	**723**	0.001249	0.000653
**88**	0.006219	0.004389	**406**	0.001931	0.000823	**724**	0.001249	0.000653
**89**	0.006219	0.004389	**407**	0.001931	0.000823	**725**	0.001249	0.000653
**90**	0.006219	0.004389	**408**	0.001931	0.000823	**726**	0.001249	0.000653

**91**	0.005659	0.004267	**409**	0.001931	0.000823	**727**	0.001249	0.000653
**92**	0.005659	0.004267	**410**	0.001931	0.000823	**728**	0.001249	0.000653
**93**	0.005659	0.004267	**411**	0.001931	0.000823	**729**	0.001249	0.000653
**94**	0.005659	0.004267	**412**	0.001931	0.000823	**730**	0.001249	0.000653
**95**	0.005659	0.004267	**413**	0.001931	0.000823	**731**	0.001249	0.000653
**96**	0.005659	0.003687	**414**	0.001931	0.000823	**732**	0.001249	0.000653
**97**	0.005659	0.003687	**415**	0.001931	0.000823	**733**	0.001249	0.000653
**98**	0.005659	0.003687	**416**	0.001931	0.000823	**734**	0.001249	0.000653
**99**	0.005659	0.003687	**417**	0.001931	0.000823	**735**	0.001249	0.000653
**100**	0.005659	0.003687	**418**	0.001931	0.000823	**736**	0.001249	0.000653
**101**	0.005659	0.003687	**419**	0.001931	0.000823	**737**	0.001249	0.000653
**102**	0.005138	0.003687	**420**	0.001931	0.000823	**738**	0.001249	0.000653
**103**	0.005138	0.003687	**421**	0.001931	0.000823	**739**	0.001249	0.000653
**104**	0.005138	0.003687	**422**	0.001931	0.000823	**740**	0.001249	0.000653
**105**	0.005138	0.003687	**423**	0.001931	0.000823	**741**	0.001249	0.000653
**106**	0.005138	0.003331	**424**	0.001931	0.000823	**742**	0.001249	0.000653
**107**	0.005138	0.003031	**425**	0.001931	0.000823	**743**	0.001249	0.000653
**108**	0.005138	0.003031	**426**	0.001931	0.000823	**744**	0.001249	0.000653
**109**	0.005138	0.003031	**427**	0.001931	0.000823	**745**	0.001249	0.000653
**110**	0.005138	0.003031	**428**	0.001931	0.000823	**746**	0.001249	0.000653
**111**	0.005138	0.003031	**429**	0.001931	0.000823	**747**	0.001249	0.000653
**112**	0.005138	0.003031	**430**	0.001931	0.000823	**748**	0.001249	0.000653
**113**	0.005138	0.002975	**431**	0.001931	0.000823	**749**	0.001249	0.000653
**114**	0.005138	0.002975	**432**	0.001931	0.000823	**750**	0.001249	0.000653
**115**	0.004946	0.00265	**433**	0.001931	0.000823	**751**	0.001249	0.000653
**116**	0.004946	0.00265	**434**	0.001931	0.000823	**752**	0.001249	0.000653
**117**	0.004946	0.00265	**435**	0.001931	0.000823	**753**	0.001249	0.000653
**118**	0.004946	0.00265	**436**	0.001931	0.000823	**754**	0.001249	0.000653
**119**	0.004946	0.00265	**437**	0.001931	0.000823	**755**	0.001249	0.000653
**120**	0.004946	0.00265	**438**	0.001931	0.000823	**756**	0.001249	0.000653
**121**	0.004946	0.00265	**439**	0.001931	0.000823	**757**	0.001249	0.000653
**122**	0.004946	0.002012	**440**	0.001931	0.000823	**758**	0.001249	0.000653
**123**	0.004946	0.002012	**441**	0.001931	0.000823	**759**	0.001249	0.000653
**124**	0.004946	0.002012	**442**	0.001931	0.000823	**760**	0.001249	0.000653
**125**	0.004946	0.002012	**443**	0.001931	0.000823	**761**	0.001249	0.000653
**126**	0.004946	0.000823	**444**	0.001931	0.000823	**762**	0.001249	0.000653
**127**	0.004946	0.000823	**445**	0.001931	0.000823	**763**	0.001249	0.000653
**128**	0.004946	0.000823	**446**	0.001931	0.000823	**764**	0.001249	0.000653
**129**	0.004946	0.000823	**447**	0.001931	0.000823	**765**	0.001249	0.000653
**130**	0.004946	0.000823	**448**	0.001931	0.000823	**766**	0.001249	0.000653
**131**	0.004946	0.000823	**449**	0.001931	0.000823	**767**	0.001249	0.000653
**132**	0.004946	0.000823	**450**	0.001931	0.000823	**768**	0.001249	0.000653
**133**	0.004946	0.000823	**451**	0.001931	0.000823	**769**	0.001249	0.000653
**134**	0.004946	0.000823	**452**	0.001931	0.000823	**770**	0.001249	0.000653
**135**	0.004946	0.000823	**453**	0.001931	0.000823	**771**	0.001249	0.000653
**136**	0.004946	0.000823	**454**	0.001931	0.000823	**772**	0.001249	0.000653

**137**	0.004946	0.000823	**455**	0.001931	0.000823	**773**	0.001249	0.000653
**138**	0.004946	0.000823	**456**	0.001931	0.000823	**774**	0.001249	0.000653
**139**	0.004946	0.000823	**457**	0.001931	0.000823	**775**	0.001249	0.000653
**140**	0.004946	0.000823	**458**	0.001931	0.000823	**776**	0.001249	0.000653
**141**	0.004946	0.000823	**459**	0.001931	0.000823	**777**	0.001249	0.000653
**142**	0.004946	0.000823	**460**	0.001931	0.000823	**778**	0.001249	0.000653
**143**	0.004946	0.000823	**461**	0.001931	0.000823	**779**	0.001249	0.000653
**144**	0.003052	0.000823	**462**	0.001931	0.000823	**780**	0.001249	0.000653
**145**	0.003052	0.000823	**463**	0.001931	0.000823	**781**	0.001249	0.000653
**146**	0.003052	0.000823	**464**	0.001931	0.000823	**782**	0.001249	0.000653
**147**	0.003052	0.000823	**465**	0.001931	0.000823	**783**	0.001249	0.000653
**148**	0.003052	0.000823	**466**	0.001931	0.000823	**784**	0.001249	0.000653
**149**	0.003052	0.000823	**467**	0.001931	0.000823	**785**	0.001249	0.000653
**150**	0.003052	0.000823	**468**	0.001931	0.000823	**786**	0.001249	0.000653
**151**	0.003052	0.000823	**469**	0.001931	0.000823	**787**	0.001249	0.000653
**152**	0.003052	0.000823	**470**	0.001931	0.000823	**788**	0.001249	0.000653
**153**	0.003052	0.000823	**471**	0.001931	0.000823	**789**	0.001249	0.000653
**154**	0.003052	0.000823	**472**	0.001931	0.000823	**790**	0.001249	0.000653
**155**	0.003052	0.000823	**473**	0.001931	0.000823	**791**	0.001249	0.000653
**156**	0.003052	0.000823	**474**	0.001931	0.000823	**792**	0.001249	0.000653
**157**	0.003052	0.000823	**475**	0.001931	0.000823	**793**	0.001249	0.000653
**158**	0.003052	0.000823	**476**	0.001931	0.000823	**794**	0.001249	0.000653
**159**	0.003052	0.000823	**477**	0.001931	0.000823	**795**	0.001249	0.000653
**160**	0.003052	0.000823	**478**	0.001931	0.000823	**796**	0.001249	0.000653
**161**	0.003052	0.000823	**479**	0.001931	0.000823	**797**	0.001249	0.000653
**162**	0.003052	0.000823	**480**	0.001931	0.000823	**798**	0.001249	0.000653
**163**	0.003052	0.000823	**481**	0.001931	0.000823	**799**	0.001249	0.000653
**164**	0.003052	0.000823	**482**	0.001931	0.000823	**800**	0.001249	0.000653
**165**	0.003052	0.000823	**483**	0.001931	0.000823	**801**	0.001249	0.000653
**166**	0.003052	0.000823	**484**	0.001931	0.000823	**802**	0.001249	0.000653
**167**	0.003052	0.000823	**485**	0.001931	0.000823	**803**	0.001249	0.000653
**168**	0.003052	0.000823	**486**	0.001931	0.000823	**804**	0.001249	0.000653
**169**	0.003052	0.000823	**487**	0.001931	0.000823	**805**	0.001249	0.000653
**170**	0.003052	0.000823	**488**	0.001931	0.000823	**806**	0.001249	0.000653
**171**	0.003052	0.000823	**489**	0.001931	0.000823	**807**	0.001249	0.000653
**172**	0.003052	0.000823	**490**	0.001931	0.000823	**808**	0.001249	0.000653
**173**	0.003052	0.000823	**491**	0.001931	0.000823	**809**	0.001249	0.000653
**174**	0.003052	0.000823	**492**	0.001931	0.000823	**810**	0.001249	0.000653
**175**	0.003052	0.000823	**493**	0.001777	0.000823	**811**	0.001249	0.000653
**176**	0.003052	0.000823	**494**	0.001777	0.000823	**812**	0.001249	0.000653
**177**	0.003052	0.000823	**495**	0.001777	0.000823	**813**	0.001249	0.000653
**178**	0.003052	0.000823	**496**	0.001777	0.000823	**814**	0.001249	0.000653
**179**	0.003052	0.000823	**497**	0.001777	0.000823	**815**	0.001249	0.000653
**180**	0.003052	0.000823	**498**	0.001777	0.000823	**816**	0.001249	0.000653
**181**	0.003052	0.000823	**499**	0.001777	0.000823	**817**	0.001249	0.000653
**182**	0.003052	0.000823	**500**	0.001777	0.000823	**818**	0.001249	0.000653

**183**	0.003052	0.000823	**501**	0.001777	0.000823	**819**	0.001249	0.000653
**184**	0.003052	0.000823	**502**	0.001777	0.000823	**820**	0.001249	0.000653
**185**	0.003052	0.000823	**503**	0.001777	0.000823	**821**	0.001249	0.000653
**186**	0.003052	0.000823	**504**	0.001777	0.000823	**822**	0.001249	0.000653
**187**	0.003052	0.000823	**505**	0.001777	0.000823	**823**	0.001249	0.000653
**188**	0.003052	0.000823	**506**	0.001777	0.000823	**824**	0.001249	0.000653
**189**	0.003052	0.000823	**507**	0.001777	0.000823	**825**	0.001249	0.000653
**190**	0.003052	0.000823	**508**	0.001777	0.000823	**826**	0.001249	0.000653
**191**	0.003052	0.000823	**509**	0.001777	0.000823	**827**	0.001249	0.000653
**192**	0.003052	0.000823	**510**	0.001777	0.000823	**828**	0.001249	0.000653
**193**	0.003052	0.000823	**511**	0.001777	0.000823	**829**	0.001249	0.000653
**194**	0.003052	0.000823	**512**	0.001777	0.000823	**830**	0.001249	0.000653
**195**	0.003052	0.000823	**513**	0.001777	0.000823	**831**	0.001249	0.000653
**196**	0.002532	0.000823	**514**	0.001777	0.000823	**832**	0.001249	0.000653
**197**	0.002532	0.000823	**515**	0.001777	0.000823	**833**	0.001249	0.000653
**198**	0.002532	0.000823	**516**	0.001777	0.000823	**834**	0.001249	0.000653
**199**	0.002532	0.000823	**517**	0.001777	0.000823	**835**	0.001249	0.000653
**200**	0.002532	0.000823	**518**	0.001777	0.000823	**836**	0.001249	0.000653
**201**	0.002532	0.000823	**519**	0.001777	0.000823	**837**	0.001249	0.000653
**202**	0.002532	0.000823	**520**	0.001777	0.000823	**838**	0.001249	0.000653
**203**	0.002532	0.000823	**521**	0.001777	0.000823	**839**	0.001249	0.000653
**204**	0.002532	0.000823	**522**	0.001777	0.000823	**840**	0.001249	0.000653
**205**	0.002532	0.000823	**523**	0.001777	0.000823	**841**	0.001249	0.000653
**206**	0.002532	0.000823	**524**	0.001777	0.000812	**842**	0.001249	0.000653
**207**	0.002532	0.000823	**525**	0.001777	0.000812	**843**	0.001249	0.000653
**208**	0.002532	0.000823	**526**	0.001777	0.000812	**844**	0.001249	0.000653
**209**	0.002532	0.000823	**527**	0.001777	0.000812	**845**	0.001249	0.000653
**210**	0.002532	0.000823	**528**	0.001777	0.000812	**846**	0.001249	0.000653
**211**	0.002532	0.000823	**529**	0.001777	0.000812	**847**	0.001249	0.000653
**212**	0.002532	0.000823	**530**	0.001777	0.000812	**848**	0.001249	0.000653
**213**	0.002532	0.000823	**531**	0.001777	0.000812	**849**	0.001249	0.000653
**214**	0.002532	0.000823	**532**	0.001777	0.000812	**850**	0.001249	0.000653
**215**	0.002532	0.000823	**533**	0.001777	0.000812	**851**	0.001249	0.000653
**216**	0.002532	0.000823	**534**	0.001777	0.000812	**852**	0.001249	0.000653
**217**	0.002532	0.000823	**535**	0.001777	0.000812	**853**	0.001249	0.000653
**218**	0.002532	0.000823	**536**	0.001777	0.000812	**854**	0.001249	0.000653
**219**	0.002532	0.000823	**537**	0.001777	0.000812	**855**	0.001249	0.000633
**220**	0.002532	0.000823	**538**	0.001777	0.000812	**856**	0.001249	0.000633
**221**	0.002532	0.000823	**539**	0.001777	0.000812	**857**	0.001249	0.000633
**222**	0.002532	0.000823	**540**	0.001777	0.000812	**858**	0.001249	0.000633
**223**	0.002532	0.000823	**541**	0.001777	0.000812	**859**	0.001249	0.000633
**224**	0.002532	0.000823	**542**	0.001777	0.000812	**860**	0.001249	0.000633
**225**	0.002532	0.000823	**543**	0.001777	0.000812	**861**	0.001249	0.000633
**226**	0.002532	0.000823	**544**	0.001777	0.000812	**862**	0.001249	0.000633
**227**	0.002532	0.000823	**545**	0.001777	0.000812	**863**	0.001249	0.000633
**228**	0.002532	0.000823	**546**	0.001777	0.000812	**864**	0.001249	0.000633

**229**	0.002532	0.000823	**547**	0.001777	0.000812	**865**	0.001249	0.000633
**230**	0.002532	0.000823	**548**	0.001672	0.000812	**866**	0.001249	0.000633
**231**	0.002532	0.000823	**549**	0.001672	0.000812	**867**	0.001249	0.000633
**232**	0.002532	0.000823	**550**	0.001672	0.000812	**868**	0.001249	0.000633
**233**	0.002532	0.000823	**551**	0.001672	0.000812	**869**	0.001249	0.000633
**234**	0.002532	0.000823	**552**	0.001672	0.000812	**870**	0.001249	0.000633
**235**	0.002532	0.000823	**553**	0.001672	0.000812	**871**	0.001249	0.000633
**236**	0.002532	0.000823	**554**	0.001672	0.000812	**872**	0.001249	0.000633
**237**	0.002532	0.000823	**555**	0.001672	0.000812	**873**	0.001249	0.000633
**238**	0.002532	0.000823	**556**	0.001672	0.000812	**874**	0.001249	0.000633
**239**	0.002532	0.000823	**557**	0.001672	0.000812	**875**	0.001249	0.000633
**240**	0.002532	0.000823	**558**	0.001672	0.000812	**876**	0.001249	0.000633
**241**	0.002532	0.000823	**559**	0.001672	0.000812	**877**	0.001249	0.000633
**242**	0.002532	0.000823	**560**	0.001672	0.000812	**878**	0.001249	0.000633
**243**	0.002532	0.000823	**561**	0.001672	0.000812	**879**	0.001249	0.000633
**244**	0.002532	0.000823	**562**	0.001672	0.000812	**880**	0.001249	0.000633
**245**	0.002532	0.000823	**563**	0.001672	0.000812	**881**	0.001249	0.000633
**246**	0.002532	0.000823	**564**	0.001672	0.000812	**882**	0.001249	0.000633
**247**	0.002532	0.000823	**565**	0.001672	0.000812	**883**	0.001249	0.000633
**248**	0.002532	0.000823	**566**	0.001672	0.000812	**884**	0.001249	0.000633
**249**	0.002532	0.000823	**567**	0.001672	0.000812	**885**	0.001249	0.000633
**250**	0.002532	0.000823	**568**	0.001672	0.000812	**886**	0.001249	0.000633
**251**	0.002532	0.000823	**569**	0.001672	0.000812	**887**	0.001249	0.000633
**252**	0.002532	0.000823	**570**	0.001672	0.000812	**888**	0.001249	0.000633
**253**	0.002532	0.000823	**571**	0.001672	0.000812	**889**	0.001249	0.000633
**254**	0.002532	0.000823	**572**	0.001672	0.000812	**890**	0.001249	0.000633
**255**	0.002532	0.000823	**573**	0.001672	0.000812	**891**	0.001249	0.000633
**256**	0.002532	0.000823	**574**	0.001615	0.000812	**892**	0.001249	0.000633
**257**	0.002532	0.000823	**575**	0.001615	0.000812	**893**	0.001249	0.000633
**258**	0.002532	0.000823	**576**	0.001391	0.000812	**894**	0.001249	0.000633
**259**	0.002532	0.000823	**577**	0.001391	0.000812	**895**	0.001249	0.000633
**260**	0.002532	0.000823	**578**	0.001391	0.000812	**896**	0.001249	0.000633
**261**	0.002532	0.000823	**579**	0.001391	0.000812	**897**	0.001249	0.000633
**262**	0.002532	0.000823	**580**	0.001391	0.000812	**898**	0.001249	0.000633
**263**	0.002532	0.000823	**581**	0.001391	0.000812	**899**	0.001249	0.000633
**264**	0.002532	0.000823	**582**	0.001391	0.000812	**900**	0.001249	0.000633
**265**	0.002532	0.000823	**583**	0.001391	0.000812	**901**	0.001249	0.000633
**266**	0.002532	0.000823	**584**	0.001391	0.000812	**902**	0.001249	0.000633
**267**	0.002532	0.000823	**585**	0.001391	0.000812	**903**	0.001249	0.000633
**268**	0.002532	0.000823	**586**	0.001391	0.000812	**904**	0.001249	0.000633
**269**	0.002532	0.000823	**587**	0.001391	0.000812	**905**	0.001249	0.000633
**270**	0.002532	0.000823	**588**	0.001391	0.000812	**906**	0.001249	0.000633
**271**	0.002532	0.000823	**589**	0.001391	0.000812	**907**	0.001249	0.000633
**272**	0.002532	0.000823	**590**	0.001391	0.000812	**908**	0.001249	0.000633
**273**	0.002532	0.000823	**591**	0.001391	0.000812	**909**	0.001249	0.000633
**274**	0.002532	0.000823	**592**	0.001391	0.000812	**910**	0.001249	0.000633

**275**	0.002532	0.000823	**593**	0.001391	0.000812	**911**	0.001249	0.000633
**276**	0.002532	0.000823	**594**	0.001391	0.000812	**912**	0.001249	0.000633
**277**	0.002532	0.000823	**595**	0.001391	0.000812	**913**	0.001249	0.000633
**278**	0.002532	0.000823	**596**	0.001391	0.000812	**914**	0.001249	0.000633
**279**	0.002532	0.000823	**597**	0.001391	0.000812	**915**	0.001249	0.000633
**280**	0.002532	0.000823	**598**	0.001391	0.000812	**916**	0.001249	0.000633
**281**	0.002532	0.000823	**599**	0.001391	0.000812	**917**	0.001249	0.000633
**282**	0.002532	0.000823	**600**	0.001391	0.000812	**918**	0.001249	0.000633
**283**	0.002532	0.000823	**601**	0.001391	0.000812	**919**	0.001249	0.000633
**284**	0.002532	0.000823	**602**	0.001391	0.000812	**920**	0.001249	0.000633
**285**	0.002532	0.000823	**603**	0.001391	0.000812	**921**	0.001249	0.000633
**286**	0.002532	0.000823	**604**	0.001391	0.000812	**922**	0.001249	0.000633
**287**	0.002532	0.000823	**605**	0.001391	0.000812	**923**	0.001249	0.000633
**288**	0.002532	0.000823	**606**	0.001391	0.000812	**924**	0.001249	0.000633
**289**	0.002532	0.000823	**607**	0.001391	0.000812	**925**	0.001249	0.000633
**290**	0.002532	0.000823	**608**	0.001391	0.000812	**926**	0.001249	0.000633
**291**	0.002532	0.000823	**609**	0.001391	0.000812	**927**	0.001249	0.000633
**292**	0.002532	0.000823	**610**	0.001391	0.000812	**928**	0.001249	0.000633
**293**	0.002532	0.000823	**611**	0.001391	0.000812	**929**	0.001249	0.000633
**294**	0.002532	0.000823	**612**	0.001391	0.000812	**930**	0.001249	0.000633
**295**	0.002532	0.000823	**613**	0.001391	0.000812	**931**	0.001249	0.000633
**296**	0.002532	0.000823	**614**	0.001391	0.000812	**932**	0.001249	0.000633
**297**	0.002532	0.000823	**615**	0.001391	0.000812	**933**	0.001249	0.000633
**298**	0.002532	0.000823	**616**	0.001391	0.000812	**934**	0.001249	0.000633
**299**	0.002532	0.000823	**617**	0.001391	0.000812	**935**	0.001249	0.000633
**300**	0.002532	0.000823	**618**	0.001391	0.000812	**936**	0.001249	0.000633
**301**	0.002532	0.000823	**619**	0.001391	0.000812	**937**	0.001249	0.000633
**302**	0.002532	0.000823	**620**	0.001391	0.000812	**938**	0.001249	0.000633
**303**	0.002532	0.000823	**621**	0.001391	0.000812	**939**	0.001249	0.000633
**304**	0.002532	0.000823	**622**	0.001391	0.000812	**940**	0.001249	0.000633
**305**	0.002458	0.000823	**623**	0.001391	0.000812	**941**	0.001249	0.000633
**306**	0.002458	0.000823	**624**	0.001391	0.000812	**942**	0.001249	0.000633
**307**	0.002458	0.000823	**625**	0.001391	0.000812	**943**	0.001249	0.000633
**308**	0.002458	0.000823	**626**	0.001391	0.000812	**944**	0.001249	0.000633
**309**	0.002458	0.000823	**627**	0.001391	0.000812	**945**	0.001249	0.000633
**310**	0.002458	0.000823	**628**	0.001391	0.000812	**946**	0.001249	0.000633
**311**	0.002458	0.000823	**629**	0.001391	0.000812	**947**	0.001249	0.000633
**312**	0.002458	0.000823	**630**	0.001391	0.000812	**948**	0.001249	0.000633
**313**	0.002315	0.000823	**631**	0.001391	0.000812	**949**	0.001249	0.000633
**314**	0.002315	0.000823	**632**	0.001391	0.000812	**950**	0.001249	0.000633
**315**	0.002315	0.000823	**633**	0.001391	0.000812			
**316**	0.002315	0.000823	**634**	0.001391	0.000812			
**317**	0.002315	0.000823	**635**	0.001391	0.000812			
**318**	0.002315	0.000823	**636**	0.001391	0.000812			

Table 18. Convergence of the first damage scenario in the presence of 5% noise for first 1 and 3 modes

Iterations	1 Mode	3 Modes	Iterations	1 Modes	3 Modes	Iterations	1 Modes	3 Modes
**1**	0.31095	0.31083	**319**	0.011431	0.008088	**637**	0.00962	0.007857
**2**	0.26327	0.2242	**320**	0.011431	0.008088	**638**	0.00962	0.007857
**3**	0.21064	0.21883	**321**	0.011431	0.008088	**639**	0.00962	0.007857
**4**	0.19439	0.15105	**322**	0.011431	0.008088	**640**	0.00962	0.007857
**5**	0.15328	0.096669	**323**	0.011431	0.008088	**641**	0.00962	0.007857
**6**	0.10942	0.067789	**324**	0.011431	0.008088	**642**	0.00962	0.007857
**7**	0.10942	0.056151	**325**	0.011431	0.008088	**643**	0.00962	0.007857
**8**	0.083234	0.056151	**326**	0.011431	0.008088	**644**	0.00962	0.007857
**9**	0.067864	0.043609	**327**	0.011431	0.008088	**645**	0.00962	0.007857
**10**	0.067864	0.039935	**328**	0.011431	0.008088	**646**	0.00962	0.007857
**11**	0.046668	0.038463	**329**	0.011431	0.008088	**647**	0.00962	0.007857
**12**	0.046668	0.03213	**330**	0.011431	0.008088	**648**	0.00962	0.007857
**13**	0.046668	0.03213	**331**	0.011431	0.008088	**649**	0.00962	0.007857
**14**	0.046668	0.02587	**332**	0.011431	0.008088	**650**	0.00962	0.007857
**15**	0.046668	0.02587	**333**	0.011431	0.008088	**651**	0.00962	0.007857
**16**	0.042393	0.02587	**334**	0.011431	0.008088	**652**	0.00962	0.007857
**17**	0.035256	0.02587	**335**	0.011431	0.008088	**653**	0.00962	0.007857
**18**	0.033253	0.02587	**336**	0.011431	0.008088	**654**	0.00962	0.007857
**19**	0.031486	0.02587	**337**	0.011431	0.008088	**655**	0.00962	0.007857
**20**	0.029319	0.024248	**338**	0.011431	0.008088	**656**	0.00962	0.007857
**21**	0.029319	0.021805	**339**	0.011431	0.008088	**657**	0.00962	0.007857
**22**	0.029319	0.018776	**340**	0.011431	0.008088	**658**	0.00962	0.007857
**23**	0.024074	0.018776	**341**	0.011431	0.008088	**659**	0.00962	0.007857
**24**	0.024074	0.018776	**342**	0.011431	0.008088	**660**	0.00962	0.007857
**25**	0.024074	0.017455	**343**	0.011431	0.008088	**661**	0.00962	0.007857
**26**	0.024074	0.017455	**344**	0.011431	0.008088	**662**	0.00962	0.007857
**27**	0.024074	0.017455	**345**	0.011431	0.008088	**663**	0.00962	0.007857
**28**	0.020756	0.017455	**346**	0.011431	0.008088	**664**	0.00962	0.007857
**29**	0.020756	0.017455	**347**	0.011431	0.008088	**665**	0.00962	0.007857
**30**	0.020756	0.017455	**348**	0.011431	0.008088	**666**	0.00962	0.007857
**31**	0.020756	0.017455	**349**	0.011431	0.008088	**667**	0.00962	0.007857
**32**	0.020756	0.01363	**350**	0.011431	0.008088	**668**	0.00962	0.007857
**33**	0.019984	0.01363	**351**	0.011431	0.008088	**669**	0.00962	0.007857
**34**	0.019984	0.01363	**352**	0.011431	0.008088	**670**	0.00962	0.007857
**35**	0.019984	0.01363	**353**	0.011431	0.008088	**671**	0.00962	0.007857
**36**	0.019984	0.01363	**354**	0.011431	0.008088	**672**	0.00962	0.007857
**37**	0.018976	0.01363	**355**	0.011431	0.008088	**673**	0.00962	0.007857
**38**	0.016763	0.01363	**356**	0.011431	0.008088	**674**	0.00962	0.007857
**39**	0.016763	0.01363	**357**	0.011431	0.008088	**675**	0.00962	0.007857
**40**	0.016763	0.01363	**358**	0.011431	0.008088	**676**	0.00962	0.007857
**41**	0.016763	0.01363	**359**	0.011431	0.008088	**677**	0.00962	0.007857
**42**	0.016763	0.01363	**360**	0.011431	0.008088	**678**	0.00962	0.007857

**43**	0.016763	0.01363	**361**	0.011431	0.008088	**679**	0.00962	0.007857
**44**	0.016763	0.01363	**362**	0.011431	0.008088	**680**	0.00962	0.007857
**45**	0.016763	0.01363	**363**	0.011431	0.008088	**681**	0.00962	0.007857
**46**	0.016763	0.01363	**364**	0.011431	0.008088	**682**	0.00962	0.007857
**47**	0.016763	0.01363	**365**	0.011431	0.008088	**683**	0.00962	0.007857
**48**	0.016763	0.01363	**366**	0.011431	0.008088	**684**	0.00962	0.007857
**49**	0.016763	0.01363	**367**	0.011431	0.008088	**685**	0.00962	0.007857
**50**	0.016763	0.01363	**368**	0.011431	0.008088	**686**	0.00962	0.007857
**51**	0.016763	0.01363	**369**	0.011431	0.008088	**687**	0.00962	0.007857
**52**	0.016763	0.01363	**370**	0.011431	0.008088	**688**	0.00962	0.007857
**53**	0.016763	0.01363	**371**	0.011431	0.008088	**689**	0.00962	0.007857
**54**	0.016763	0.01363	**372**	0.011431	0.008088	**690**	0.00962	0.007857
**55**	0.016763	0.01363	**373**	0.011431	0.008088	**691**	0.00962	0.007857
**56**	0.016763	0.013384	**374**	0.011431	0.008088	**692**	0.00962	0.007857
**57**	0.016763	0.011586	**375**	0.011431	0.008088	**693**	0.00962	0.007857
**58**	0.016763	0.011586	**376**	0.011431	0.008088	**694**	0.00962	0.007857
**59**	0.016763	0.011586	**377**	0.011431	0.008088	**695**	0.00962	0.007857
**60**	0.015877	0.011586	**378**	0.011431	0.008088	**696**	0.00962	0.007857
**61**	0.015877	0.011586	**379**	0.011431	0.008088	**697**	0.00962	0.007857
**62**	0.015877	0.010113	**380**	0.011431	0.008088	**698**	0.00962	0.007857
**63**	0.014837	0.010113	**381**	0.011431	0.008088	**699**	0.00962	0.007857
**64**	0.014837	0.010113	**382**	0.011431	0.008088	**700**	0.00962	0.007857
**65**	0.014837	0.010113	**383**	0.011431	0.008088	**701**	0.00962	0.007857
**66**	0.014837	0.010113	**384**	0.011431	0.008088	**702**	0.00962	0.007857
**67**	0.014837	0.010113	**385**	0.011431	0.008088	**703**	0.00962	0.007857
**68**	0.014837	0.010113	**386**	0.011431	0.008088	**704**	0.00962	0.007857
**69**	0.014837	0.010113	**387**	0.011431	0.008088	**705**	0.00962	0.007857
**70**	0.014837	0.010113	**388**	0.011431	0.008088	**706**	0.00962	0.007857
**71**	0.014837	0.010113	**389**	0.011431	0.008088	**707**	0.00962	0.007857
**72**	0.014837	0.010113	**390**	0.011431	0.008088	**708**	0.00962	0.007857
**73**	0.014837	0.009698	**391**	0.011431	0.008088	**709**	0.00962	0.007857
**74**	0.014837	0.009698	**392**	0.011431	0.008088	**710**	0.00962	0.007857
**75**	0.014837	0.009698	**393**	0.011431	0.008088	**711**	0.00962	0.007857
**76**	0.014837	0.009698	**394**	0.011431	0.008088	**712**	0.00962	0.007857
**77**	0.014837	0.009698	**395**	0.011431	0.008088	**713**	0.00962	0.007857
**78**	0.014837	0.009698	**396**	0.011431	0.008088	**714**	0.00962	0.007857
**79**	0.014837	0.009698	**397**	0.011431	0.008088	**715**	0.00962	0.007857
**80**	0.014837	0.009698	**398**	0.011431	0.008088	**716**	0.00962	0.007857
**81**	0.014837	0.009698	**399**	0.011431	0.008088	**717**	0.00962	0.007857
**82**	0.014837	0.009666	**400**	0.011431	0.008088	**718**	0.00962	0.007857
**83**	0.014837	0.009666	**401**	0.011431	0.008088	**719**	0.00962	0.007857
**84**	0.014837	0.009666	**402**	0.011431	0.008088	**720**	0.00962	0.007857
**85**	0.014837	0.009666	**403**	0.011431	0.008088	**721**	0.00962	0.007857
**86**	0.014837	0.009666	**404**	0.011431	0.008088	**722**	0.00962	0.007857
**87**	0.014837	0.009666	**405**	0.011431	0.008088	**723**	0.00962	0.007857
**88**	0.014837	0.009666	**406**	0.011431	0.008088	**724**	0.00962	0.007857

**89**	0.014837	0.009666	**407**	0.011431	0.008088	**725**	0.00962	0.007857
**90**	0.014837	0.009666	**408**	0.011431	0.008088	**726**	0.00962	0.007857
**91**	0.014837	0.009666	**409**	0.011431	0.008088	**727**	0.00962	0.007857
**92**	0.014837	0.009666	**410**	0.011431	0.008088	**728**	0.00962	0.007857
**93**	0.014837	0.009666	**411**	0.011431	0.008088	**729**	0.00962	0.007857
**94**	0.014837	0.009666	**412**	0.011431	0.008088	**730**	0.00962	0.007857
**95**	0.014837	0.009666	**413**	0.011431	0.008088	**731**	0.00962	0.007857
**96**	0.014837	0.009666	**414**	0.011431	0.008088	**732**	0.00962	0.007857
**97**	0.014837	0.009666	**415**	0.011431	0.008088	**733**	0.00962	0.007857
**98**	0.014837	0.009666	**416**	0.011431	0.008088	**734**	0.00962	0.007857
**99**	0.014837	0.009666	**417**	0.011431	0.008088	**735**	0.00962	0.007857
**100**	0.014837	0.009666	**418**	0.011431	0.008088	**736**	0.00962	0.007857
**101**	0.014837	0.009666	**419**	0.011431	0.008088	**737**	0.00962	0.007857
**102**	0.014837	0.009666	**420**	0.011431	0.008088	**738**	0.00962	0.007857
**103**	0.014837	0.009666	**421**	0.011431	0.008088	**739**	0.00962	0.007857
**104**	0.014837	0.009666	**422**	0.011431	0.008088	**740**	0.00962	0.007857
**105**	0.014837	0.009666	**423**	0.011431	0.008088	**741**	0.00962	0.007857
**106**	0.014837	0.009666	**424**	0.011431	0.008088	**742**	0.00962	0.007857
**107**	0.014837	0.009666	**425**	0.011431	0.008088	**743**	0.00962	0.007857
**108**	0.014837	0.009666	**426**	0.011431	0.008088	**744**	0.009234	0.007857
**109**	0.014837	0.009666	**427**	0.011431	0.008088	**745**	0.009234	0.007857
**110**	0.014837	0.009666	**428**	0.011431	0.008088	**746**	0.009234	0.007857
**111**	0.014837	0.009666	**429**	0.011431	0.008088	**747**	0.009234	0.007857
**112**	0.014837	0.009666	**430**	0.011431	0.008088	**748**	0.009234	0.007857
**113**	0.014837	0.009666	**431**	0.011431	0.008088	**749**	0.009234	0.007857
**114**	0.014837	0.009666	**432**	0.011431	0.008088	**750**	0.009234	0.007857
**115**	0.014837	0.009666	**433**	0.011431	0.008088	**751**	0.008691	0.007857
**116**	0.014837	0.009666	**434**	0.011431	0.008088	**752**	0.008691	0.007857
**117**	0.014837	0.009666	**435**	0.011431	0.008088	**753**	0.008691	0.007857
**118**	0.014837	0.009666	**436**	0.011431	0.008088	**754**	0.008691	0.007857
**119**	0.014837	0.009666	**437**	0.011431	0.008088	**755**	0.008691	0.007857
**120**	0.014837	0.009666	**438**	0.011431	0.008088	**756**	0.008691	0.007857
**121**	0.014837	0.009666	**439**	0.011431	0.008088	**757**	0.008691	0.007857
**122**	0.014837	0.009666	**440**	0.011431	0.008088	**758**	0.008691	0.007857
**123**	0.014837	0.009666	**441**	0.011431	0.008088	**759**	0.008691	0.007857
**124**	0.014837	0.009666	**442**	0.011431	0.008088	**760**	0.008691	0.007857
**125**	0.014837	0.009666	**443**	0.011431	0.008088	**761**	0.008691	0.007857
**126**	0.014837	0.009666	**444**	0.011431	0.008088	**762**	0.008691	0.007857
**127**	0.014837	0.009666	**445**	0.011431	0.008088	**763**	0.008691	0.007857
**128**	0.014837	0.009666	**446**	0.011431	0.008088	**764**	0.008691	0.007857
**129**	0.014837	0.009666	**447**	0.011431	0.008088	**765**	0.008691	0.007857
**130**	0.014837	0.009666	**448**	0.011431	0.008088	**766**	0.008691	0.007857
**131**	0.014837	0.009666	**449**	0.011431	0.008088	**767**	0.008691	0.007857
**132**	0.014837	0.009666	**450**	0.011431	0.008088	**768**	0.008691	0.007857
**133**	0.014837	0.009666	**451**	0.011431	0.008088	**769**	0.008691	0.007857
**134**	0.014837	0.009666	**452**	0.011431	0.008088	**770**	0.008691	0.007857

**135**	0.014837	0.009666	**453**	0.011431	0.008088	**771**	0.008691	0.007857
**136**	0.014837	0.009666	**454**	0.011431	0.008088	**772**	0.008691	0.007857
**137**	0.014837	0.009666	**455**	0.011431	0.008088	**773**	0.008691	0.007857
**138**	0.014837	0.009666	**456**	0.011431	0.008088	**774**	0.008691	0.007857
**139**	0.014837	0.009666	**457**	0.011431	0.008088	**775**	0.008691	0.007857
**140**	0.014837	0.009666	**458**	0.011431	0.008088	**776**	0.008691	0.007857
**141**	0.014837	0.009666	**459**	0.011431	0.008088	**777**	0.008691	0.007857
**142**	0.014837	0.009666	**460**	0.011431	0.008088	**778**	0.008691	0.007857
**143**	0.014837	0.009666	**461**	0.010862	0.008088	**779**	0.008691	0.007857
**144**	0.014837	0.009666	**462**	0.010862	0.007857	**780**	0.008691	0.007857
**145**	0.014837	0.009666	**463**	0.010862	0.007857	**781**	0.008691	0.007857
**146**	0.014837	0.009666	**464**	0.010862	0.007857	**782**	0.008691	0.007857
**147**	0.014837	0.009666	**465**	0.010862	0.007857	**783**	0.008691	0.007857
**148**	0.014837	0.009666	**466**	0.010862	0.007857	**784**	0.008691	0.007857
**149**	0.014837	0.009666	**467**	0.010862	0.007857	**785**	0.008691	0.007857
**150**	0.014837	0.009666	**468**	0.010862	0.007857	**786**	0.008691	0.007857
**151**	0.014837	0.009666	**469**	0.010862	0.007857	**787**	0.008691	0.007857
**152**	0.014837	0.009666	**470**	0.010862	0.007857	**788**	0.008691	0.007857
**153**	0.014837	0.009666	**471**	0.010862	0.007857	**789**	0.008691	0.007857
**154**	0.014837	0.009666	**472**	0.010862	0.007857	**790**	0.008691	0.007857
**155**	0.014837	0.009666	**473**	0.010862	0.007857	**791**	0.008691	0.007857
**156**	0.014837	0.009666	**474**	0.010862	0.007857	**792**	0.008691	0.007857
**157**	0.014837	0.009666	**475**	0.010862	0.007857	**793**	0.008691	0.007857
**158**	0.014837	0.009666	**476**	0.010862	0.007857	**794**	0.008691	0.007857
**159**	0.014837	0.009666	**477**	0.010862	0.007857	**795**	0.008691	0.007857
**160**	0.014837	0.009666	**478**	0.010862	0.007857	**796**	0.008691	0.007857
**161**	0.014837	0.009666	**479**	0.010862	0.007857	**797**	0.008691	0.007857
**162**	0.014837	0.009666	**480**	0.010862	0.007857	**798**	0.008431	0.007857
**163**	0.014837	0.009666	**481**	0.010862	0.007857	**799**	0.008428	0.007857
**164**	0.014837	0.009666	**482**	0.010862	0.007857	**800**	0.008428	0.007857
**165**	0.014837	0.009666	**483**	0.010862	0.007857	**801**	0.008428	0.007857
**166**	0.014837	0.009666	**484**	0.010862	0.007857	**802**	0.008428	0.007857
**167**	0.014837	0.009456	**485**	0.010862	0.007857	**803**	0.008428	0.007857
**168**	0.014837	0.009456	**486**	0.010862	0.007857	**804**	0.008428	0.007857
**169**	0.014837	0.009456	**487**	0.010862	0.007857	**805**	0.008428	0.007857
**170**	0.014837	0.009456	**488**	0.010862	0.007857	**806**	0.008428	0.007857
**171**	0.014837	0.009456	**489**	0.010862	0.007857	**807**	0.008428	0.007857
**172**	0.014837	0.009456	**490**	0.010862	0.007857	**808**	0.008428	0.007857
**173**	0.014837	0.009456	**491**	0.010862	0.007857	**809**	0.008428	0.007857
**174**	0.014837	0.009456	**492**	0.010862	0.007857	**810**	0.008428	0.007857
**175**	0.014837	0.009456	**493**	0.010862	0.007857	**811**	0.008428	0.007857
**176**	0.014837	0.009456	**494**	0.010862	0.007857	**812**	0.008428	0.007857
**177**	0.014837	0.009456	**495**	0.010862	0.007857	**813**	0.008428	0.007857
**178**	0.014837	0.009456	**496**	0.010862	0.007857	**814**	0.008428	0.007857
**179**	0.014837	0.009456	**497**	0.010862	0.007857	**815**	0.008428	0.007857
**180**	0.014837	0.009456	**498**	0.010862	0.007857	**816**	0.008428	0.007857

**181**	0.014837	0.00914	**499**	0.010862	0.007857	**817**	0.008428	0.007857
**182**	0.014837	0.00914	**500**	0.010862	0.007857	**818**	0.008428	0.007857
**183**	0.014837	0.00914	**501**	0.010862	0.007857	**819**	0.008428	0.007857
**184**	0.014837	0.00914	**502**	0.010862	0.007857	**820**	0.008428	0.007857
**185**	0.014837	0.00914	**503**	0.010862	0.007857	**821**	0.008428	0.007857
**186**	0.014837	0.00914	**504**	0.010862	0.007857	**822**	0.008428	0.007857
**187**	0.014837	0.00914	**505**	0.010862	0.007857	**823**	0.008428	0.007857
**188**	0.014837	0.00914	**506**	0.010862	0.007857	**824**	0.008428	0.007857
**189**	0.014837	0.00914	**507**	0.010862	0.007857	**825**	0.008428	0.007857
**190**	0.014837	0.00914	**508**	0.010862	0.007857	**826**	0.008428	0.007857
**191**	0.014837	0.00914	**509**	0.010862	0.007857	**827**	0.008428	0.007857
**192**	0.014837	0.00914	**510**	0.010862	0.007857	**828**	0.008428	0.007857
**193**	0.014837	0.00914	**511**	0.010862	0.007857	**829**	0.008428	0.007857
**194**	0.014837	0.00914	**512**	0.010862	0.007857	**830**	0.008428	0.007857
**195**	0.014837	0.00914	**513**	0.010862	0.007857	**831**	0.008428	0.007857
**196**	0.014837	0.00914	**514**	0.010862	0.007857	**832**	0.008428	0.007857
**197**	0.014837	0.00914	**515**	0.010862	0.007857	**833**	0.007354	0.007857
**198**	0.014837	0.00914	**516**	0.010862	0.007857	**834**	0.007354	0.007857
**199**	0.014837	0.00914	**517**	0.010862	0.007857	**835**	0.007354	0.007857
**200**	0.014837	0.00914	**518**	0.010862	0.007857	**836**	0.007354	0.007857
**201**	0.014837	0.00914	**519**	0.010862	0.007857	**837**	0.007354	0.007857
**202**	0.014837	0.00914	**520**	0.010862	0.007857	**838**	0.007354	0.007857
**203**	0.014837	0.00914	**521**	0.010862	0.007857	**839**	0.007354	0.007857
**204**	0.014837	0.00914	**522**	0.010862	0.007857	**840**	0.007354	0.007857
**205**	0.014837	0.00914	**523**	0.010862	0.007857	**841**	0.007354	0.007857
**206**	0.014837	0.00914	**524**	0.010862	0.007857	**842**	0.007354	0.007857
**207**	0.014837	0.00914	**525**	0.010862	0.007857	**843**	0.007354	0.007857
**208**	0.014837	0.00914	**526**	0.010862	0.007857	**844**	0.007354	0.007857
**209**	0.014837	0.00914	**527**	0.010862	0.007857	**845**	0.007354	0.007857
**210**	0.014837	0.00914	**528**	0.010862	0.007857	**846**	0.007354	0.007857
**211**	0.014837	0.00914	**529**	0.010862	0.007857	**847**	0.007354	0.007857
**212**	0.014837	0.00914	**530**	0.010862	0.007857	**848**	0.007354	0.007857
**213**	0.014837	0.00914	**531**	0.010862	0.007857	**849**	0.007354	0.007857
**214**	0.014837	0.00914	**532**	0.010862	0.007857	**850**	0.007354	0.007857
**215**	0.014837	0.00914	**533**	0.010862	0.007857	**851**	0.007354	0.007857
**216**	0.014837	0.00914	**534**	0.010862	0.007857	**852**	0.007354	0.007857
**217**	0.014837	0.00914	**535**	0.010862	0.007857	**853**	0.007354	0.007857
**218**	0.014837	0.00914	**536**	0.010862	0.007857	**854**	0.007354	0.007857
**219**	0.014837	0.00914	**537**	0.010862	0.007857	**855**	0.007354	0.007857
**220**	0.014837	0.00914	**538**	0.010862	0.007857	**856**	0.007354	0.007857
**221**	0.014837	0.00914	**539**	0.010862	0.007857	**857**	0.007354	0.007857
**222**	0.014837	0.00914	**540**	0.010862	0.007857	**858**	0.007354	0.007857
**223**	0.014837	0.00914	**541**	0.010862	0.007857	**859**	0.006463	0.007857
**224**	0.014837	0.00914	**542**	0.010862	0.007857	**860**	0.006463	0.007857
**225**	0.014837	0.00914	**543**	0.010862	0.007857	**861**	0.006463	0.007857
**226**	0.014837	0.00914	**544**	0.010862	0.007857	**862**	0.006463	0.007857

**227**	0.014837	0.00914	**545**	0.010862	0.007857	**863**	0.006463	0.007857
**228**	0.014837	0.00914	**546**	0.010862	0.007857	**864**	0.006463	0.007857
**229**	0.014837	0.00914	**547**	0.010862	0.007857	**865**	0.006463	0.007857
**230**	0.014837	0.00914	**548**	0.010862	0.007857	**866**	0.006463	0.007857
**231**	0.014837	0.00914	**549**	0.00962	0.007857	**867**	0.006463	0.007857
**232**	0.014837	0.00914	**550**	0.00962	0.007857	**868**	0.006463	0.007857
**233**	0.014837	0.00914	**551**	0.00962	0.007857	**869**	0.006463	0.007857
**234**	0.014837	0.00914	**552**	0.00962	0.007857	**870**	0.006463	0.007857
**235**	0.014837	0.00914	**553**	0.00962	0.007857	**871**	0.006463	0.007857
**236**	0.014837	0.00914	**554**	0.00962	0.007857	**872**	0.006463	0.007857
**237**	0.014837	0.00914	**555**	0.00962	0.007857	**873**	0.006463	0.007857
**238**	0.014837	0.00914	**556**	0.00962	0.007857	**874**	0.006463	0.007857
**239**	0.014837	0.00914	**557**	0.00962	0.007857	**875**	0.006463	0.007857
**240**	0.014837	0.00914	**558**	0.00962	0.007857	**876**	0.006463	0.007857
**241**	0.014484	0.00914	**559**	0.00962	0.007857	**877**	0.006463	0.007857
**242**	0.014484	0.008337	**560**	0.00962	0.007857	**878**	0.006463	0.007857
**243**	0.014484	0.008337	**561**	0.00962	0.007857	**879**	0.006463	0.007857
**244**	0.014484	0.008337	**562**	0.00962	0.007857	**880**	0.006463	0.007857
**245**	0.014484	0.008337	**563**	0.00962	0.007857	**881**	0.006463	0.007856
**246**	0.014484	0.008337	**564**	0.00962	0.007857	**882**	0.006463	0.007856
**247**	0.014484	0.008337	**565**	0.00962	0.007857	**883**	0.006463	0.007856
**248**	0.014484	0.008337	**566**	0.00962	0.007857	**884**	0.006463	0.007856
**249**	0.014484	0.008337	**567**	0.00962	0.007857	**885**	0.006463	0.007856
**250**	0.014484	0.008337	**568**	0.00962	0.007857	**886**	0.006463	0.007856
**251**	0.014484	0.008337	**569**	0.00962	0.007857	**887**	0.006463	0.007856
**252**	0.014484	0.008337	**570**	0.00962	0.007857	**888**	0.006463	0.007856
**253**	0.014484	0.008337	**571**	0.00962	0.007857	**889**	0.006463	0.007856
**254**	0.014484	0.008337	**572**	0.00962	0.007857	**890**	0.006463	0.007856
**255**	0.014484	0.008337	**573**	0.00962	0.007857	**891**	0.006463	0.007856
**256**	0.014484	0.008337	**574**	0.00962	0.007857	**892**	0.006463	0.007856
**257**	0.014484	0.008337	**575**	0.00962	0.007857	**893**	0.006463	0.007643
**258**	0.014484	0.008337	**576**	0.00962	0.007857	**894**	0.006463	0.007643
**259**	0.014484	0.008337	**577**	0.00962	0.007857	**895**	0.006463	0.007643
**260**	0.014484	0.008337	**578**	0.00962	0.007857	**896**	0.006463	0.007643
**261**	0.014484	0.008337	**579**	0.00962	0.007857	**897**	0.006463	0.007643
**262**	0.014267	0.008337	**580**	0.00962	0.007857	**898**	0.006463	0.007643
**263**	0.014267	0.008337	**581**	0.00962	0.007857	**899**	0.006463	0.007643
**264**	0.014267	0.008337	**582**	0.00962	0.007857	**900**	0.006463	0.007643
**265**	0.014267	0.008337	**583**	0.00962	0.007857	**901**	0.006463	0.007643
**266**	0.014267	0.008337	**584**	0.00962	0.007857	**902**	0.006463	0.007643
**267**	0.014267	0.008337	**585**	0.00962	0.007857	**903**	0.006463	0.007643
**268**	0.014267	0.008337	**586**	0.00962	0.007857	**904**	0.006463	0.007643
**269**	0.014267	0.008337	**587**	0.00962	0.007857	**905**	0.006128	0.007643
**270**	0.014267	0.008088	**588**	0.00962	0.007857	**906**	0.006128	0.007643
**271**	0.014267	0.008088	**589**	0.00962	0.007857	**907**	0.006128	0.007643
**272**	0.014267	0.008088	**590**	0.00962	0.007857	**908**	0.006128	0.007643

**273**	0.01414	0.008088	**591**	0.00962	0.007857	**909**	0.006128	0.007643
**274**	0.01414	0.008088	**592**	0.00962	0.007857	**910**	0.006128	0.007643
**275**	0.01414	0.008088	**593**	0.00962	0.007857	**911**	0.006128	0.007643
**276**	0.013626	0.008088	**594**	0.00962	0.007857	**912**	0.006128	0.007643
**277**	0.013626	0.008088	**595**	0.00962	0.007857	**913**	0.006128	0.007643
**278**	0.013626	0.008088	**596**	0.00962	0.007857	**914**	0.006128	0.007643
**279**	0.013626	0.008088	**597**	0.00962	0.007857	**915**	0.006128	0.007643
**280**	0.013626	0.008088	**598**	0.00962	0.007857	**916**	0.006128	0.007643
**281**	0.013626	0.008088	**599**	0.00962	0.007857	**917**	0.006128	0.007643
**282**	0.013626	0.008088	**600**	0.00962	0.007857	**918**	0.006128	0.007643
**283**	0.013626	0.008088	**601**	0.00962	0.007857	**919**	0.006128	0.007643
**284**	0.013626	0.008088	**602**	0.00962	0.007857	**920**	0.006128	0.007643
**285**	0.013626	0.008088	**603**	0.00962	0.007857	**921**	0.006128	0.007643
**286**	0.013626	0.008088	**604**	0.00962	0.007857	**922**	0.006128	0.007643
**287**	0.013626	0.008088	**605**	0.00962	0.007857	**923**	0.006128	0.007643
**288**	0.013626	0.008088	**606**	0.00962	0.007857	**924**	0.006128	0.007643
**289**	0.013626	0.008088	**607**	0.00962	0.007857	**925**	0.006128	0.007643
**290**	0.013626	0.008088	**608**	0.00962	0.007857	**926**	0.006128	0.007643
**291**	0.013626	0.008088	**609**	0.00962	0.007857	**927**	0.006128	0.007643
**292**	0.013626	0.008088	**610**	0.00962	0.007857	**928**	0.006128	0.007643
**293**	0.013626	0.008088	**611**	0.00962	0.007857	**929**	0.006052	0.007643
**294**	0.011443	0.008088	**612**	0.00962	0.007857	**930**	0.006052	0.007643
**295**	0.011443	0.008088	**613**	0.00962	0.007857	**931**	0.006052	0.007643
**296**	0.011443	0.008088	**614**	0.00962	0.007857	**932**	0.006052	0.007643
**297**	0.011443	0.008088	**615**	0.00962	0.007857	**933**	0.006052	0.007643
**298**	0.011443	0.008088	**616**	0.00962	0.007857	**934**	0.006052	0.007643
**299**	0.011443	0.008088	**617**	0.00962	0.007857	**935**	0.006052	0.007643
**300**	0.011443	0.008088	**618**	0.00962	0.007857	**936**	0.006052	0.007643
**301**	0.011443	0.008088	**619**	0.00962	0.007857	**937**	0.006052	0.007643
**302**	0.011431	0.008088	**620**	0.00962	0.007857	**938**	0.006052	0.007643
**303**	0.011431	0.008088	**621**	0.00962	0.007857	**939**	0.006052	0.007643
**304**	0.011431	0.008088	**622**	0.00962	0.007857	**940**	0.006052	0.007643
**305**	0.011431	0.008088	**623**	0.00962	0.007857	**941**	0.006052	0.007643
**306**	0.011431	0.008088	**624**	0.00962	0.007857	**942**	0.006052	0.007643
**307**	0.011431	0.008088	**625**	0.00962	0.007857	**943**	0.006052	0.007643
**308**	0.011431	0.008088	**626**	0.00962	0.007857	**944**	0.006052	0.007643
**309**	0.011431	0.008088	**627**	0.00962	0.007857	**945**	0.006052	0.007643
**310**	0.011431	0.008088	**628**	0.00962	0.007857	**946**	0.006052	0.007643
**311**	0.011431	0.008088	**629**	0.00962	0.007857	**947**	0.006052	0.007643
**312**	0.011431	0.008088	**630**	0.00962	0.007857	**948**	0.006052	0.007643
**313**	0.011431	0.008088	**631**	0.00962	0.007857	**949**	0.006052	0.007643
**314**	0.011431	0.008088	**632**	0.00962	0.007857	**950**	0.006052	0.007643
**315**	0.011431	0.008088	**633**	0.00962	0.007857			
**316**	0.011431	0.008088	**634**	0.00962	0.007857			
**317**	0.011431	0.008088	**635**	0.00962	0.007857			
**318**	0.011431	0.008088	**636**	0.00962	0.007857			

Table 19. Convergence of the third damage scenario in the absence of noise for first 1 and 3 modes

Iterations	1 Mode	3 Modes	Iterations	1 Modes	3 Modes	Iterations	1 Modes	3 Modes
**1**	0.31355	0.26322	**319**	0.023789	0.024258	**637**	0.020317	0.022034
**2**	0.25988	0.19488	**320**	0.023789	0.024258	**638**	0.020317	0.022034
**3**	0.2167	0.16147	**321**	0.023789	0.024258	**639**	0.020317	0.022034
**4**	0.18745	0.13379	**322**	0.023789	0.024258	**640**	0.020317	0.022034
**5**	0.15331	0.088143	**323**	0.023789	0.024258	**641**	0.020317	0.022034
**6**	0.14567	0.06619	**324**	0.023789	0.024258	**642**	0.020317	0.022034
**7**	0.12098	0.06619	**325**	0.023789	0.024258	**643**	0.020317	0.022034
**8**	0.1126	0.061683	**326**	0.023789	0.024258	**644**	0.020317	0.022034
**9**	0.089663	0.055658	**327**	0.023789	0.024258	**645**	0.020317	0.022034
**10**	0.054751	0.050939	**328**	0.023789	0.024258	**646**	0.020317	0.022034
**11**	0.054751	0.043701	**329**	0.023789	0.024258	**647**	0.020317	0.022034
**12**	0.054751	0.043701	**330**	0.023789	0.024258	**648**	0.020317	0.022034
**13**	0.050396	0.043701	**331**	0.023789	0.024258	**649**	0.020317	0.022034
**14**	0.050396	0.037779	**332**	0.023789	0.024258	**650**	0.020317	0.022034
**15**	0.050396	0.037779	**333**	0.023789	0.024258	**651**	0.020317	0.022034
**16**	0.050396	0.037779	**334**	0.023789	0.024258	**652**	0.020317	0.022034
**17**	0.050396	0.037779	**335**	0.023789	0.024258	**653**	0.020317	0.022034
**18**	0.050396	0.037512	**336**	0.023789	0.024258	**654**	0.020317	0.022034
**19**	0.035266	0.037512	**337**	0.023789	0.024258	**655**	0.020317	0.022034
**20**	0.035266	0.03458	**338**	0.023789	0.024258	**656**	0.020317	0.022034
**21**	0.035266	0.03458	**339**	0.023789	0.024258	**657**	0.020317	0.022034
**22**	0.035266	0.034149	**340**	0.023789	0.024258	**658**	0.020317	0.022034
**23**	0.035266	0.034149	**341**	0.023789	0.024258	**659**	0.020317	0.022034
**24**	0.035266	0.03304	**342**	0.023789	0.024258	**660**	0.020317	0.022034
**25**	0.035266	0.032725	**343**	0.023789	0.024258	**661**	0.020317	0.022034
**26**	0.035266	0.031795	**344**	0.023789	0.024258	**662**	0.020317	0.022034
**27**	0.035266	0.029669	**345**	0.023789	0.024258	**663**	0.020317	0.022034
**28**	0.035266	0.023527	**346**	0.023789	0.024258	**664**	0.020317	0.022034
**29**	0.035266	0.022013	**347**	0.023789	0.024258	**665**	0.020317	0.022034
**30**	0.035266	0.012978	**348**	0.023789	0.024258	**666**	0.020317	0.022034
**31**	0.035266	0.012978	**349**	0.022354	0.024223	**667**	0.020317	0.022034
**32**	0.035266	0.012978	**350**	0.022354	0.024223	**668**	0.020317	0.022034
**33**	0.035266	0.012978	**351**	0.022354	0.024223	**669**	0.020317	0.022034
**34**	0.035266	0.012978	**352**	0.022354	0.024223	**670**	0.020317	0.022034
**35**	0.035266	0.012978	**353**	0.022354	0.024223	**671**	0.020317	0.022034
**36**	0.029598	0.012978	**354**	0.022354	0.024223	**672**	0.020317	0.022034
**37**	0.029598	0.012978	**355**	0.022354	0.024223	**673**	0.020317	0.022034
**38**	0.029598	0.012978	**356**	0.022354	0.024223	**674**	0.020317	0.022034
**39**	0.029598	0.012978	**357**	0.022354	0.024223	**675**	0.020317	0.022034
**40**	0.029598	0.012978	**358**	0.022354	0.024223	**676**	0.020317	0.022034
**41**	0.029263	0.012978	**359**	0.022354	0.024223	**677**	0.020317	0.022034
**42**	0.029263	0.012978	**360**	0.022354	0.024223	**678**	0.020317	0.022034
**43**	0.029263	0.012978	**361**	0.022354	0.023969	**679**	0.020317	0.022034
**44**	0.029263	0.012978	**362**	0.022354	0.023969	**680**	0.020317	0.022034

**45**	0.029263	0.012978	**363**	0.022354	0.023969	**681**	0.020317	0.022034
**46**	0.025839	0.012978	**364**	0.022354	0.023969	**682**	0.020317	0.022034
**47**	0.025839	0.012978	**365**	0.022354	0.023969	**683**	0.020317	0.022034
**48**	0.025839	0.012978	**366**	0.022354	0.023969	**684**	0.020317	0.022034
**49**	0.025839	0.012978	**367**	0.022354	0.023969	**685**	0.020317	0.022034
**50**	0.025839	0.012978	**368**	0.022354	0.023969	**686**	0.020317	0.022034
**51**	0.025839	0.012978	**369**	0.022354	0.023969	**687**	0.020317	0.022034
**52**	0.025839	0.012978	**370**	0.022354	0.023969	**688**	0.020317	0.022034
**53**	0.025839	0.012978	**371**	0.022354	0.023969	**689**	0.020317	0.022034
**54**	0.025839	0.012978	**372**	0.022354	0.023969	**690**	0.020317	0.022034
**55**	0.025839	0.012978	**373**	0.022354	0.023969	**691**	0.020317	0.022034
**56**	0.025839	0.012978	**374**	0.022354	0.023969	**692**	0.020317	0.022034
**57**	0.025839	0.012978	**375**	0.022354	0.023969	**693**	0.020317	0.022034
**58**	0.025839	0.012727	**376**	0.022354	0.023969	**694**	0.020317	0.022034
**59**	0.025839	0.012727	**377**	0.022354	0.023969	**695**	0.020317	0.022034
**60**	0.025839	0.012727	**378**	0.022354	0.023969	**696**	0.020317	0.022034
**61**	0.025839	0.012727	**379**	0.022354	0.023969	**697**	0.020317	0.022034
**62**	0.025839	0.012727	**380**	0.022354	0.023969	**698**	0.020317	0.022034
**63**	0.025839	0.012727	**381**	0.022354	0.023969	**699**	0.020317	0.022034
**64**	0.025839	0.010524	**382**	0.022354	0.023969	**700**	0.020317	0.022034
**65**	0.025839	0.010524	**383**	0.022354	0.023969	**701**	0.020317	0.022034
**66**	0.025839	0.010524	**384**	0.022354	0.023969	**702**	0.020317	0.022034
**67**	0.025839	0.010524	**385**	0.022354	0.023969	**703**	0.020317	0.022034
**68**	0.025839	0.009843	**386**	0.022354	0.023969	**704**	0.020317	0.022034
**69**	0.025839	0.009843	**387**	0.022354	0.023969	**705**	0.020317	0.022034
**70**	0.025839	0.009843	**388**	0.022354	0.023969	**706**	0.020317	0.022034
**71**	0.025839	0.009843	**389**	0.022354	0.023969	**707**	0.020317	0.022034
**72**	0.025839	0.009843	**390**	0.022354	0.023969	**708**	0.020317	0.022034
**73**	0.025839	0.009843	**391**	0.022354	0.023969	**709**	0.020317	0.022034
**74**	0.025839	0.009843	**392**	0.022354	0.023969	**710**	0.020317	0.022034
**75**	0.025839	0.008766	**393**	0.022354	0.023969	**711**	0.020317	0.022034
**76**	0.025839	0.008766	**394**	0.022354	0.023969	**712**	0.020317	0.022034
**77**	0.025839	0.008766	**395**	0.022354	0.023969	**713**	0.020317	0.022034
**78**	0.025839	0.008766	**396**	0.022354	0.023969	**714**	0.020317	0.022034
**79**	0.025839	0.008766	**397**	0.022354	0.023206	**715**	0.020317	0.022034
**80**	0.025839	0.008766	**398**	0.022354	0.023206	**716**	0.020317	0.022034
**81**	0.025839	0.008766	**399**	0.022354	0.023206	**717**	0.020317	0.022034
**82**	0.025839	0.008766	**400**	0.022354	0.023206	**718**	0.020317	0.022034
**83**	0.025839	0.008766	**401**	0.022354	0.023206	**719**	0.020317	0.022034
**84**	0.019617	0.008766	**402**	0.022354	0.023206	**720**	0.020317	0.022034
**85**	0.019617	0.008766	**403**	0.022354	0.023206	**721**	0.020317	0.022034
**86**	0.019617	0.008766	**404**	0.022354	0.023206	**722**	0.020317	0.022034
**87**	0.019617	0.008766	**405**	0.022354	0.023206	**723**	0.020317	0.022034
**88**	0.019617	0.008766	**406**	0.022354	0.023206	**724**	0.020317	0.022034
**89**	0.019617	0.008766	**407**	0.022354	0.023206	**725**	0.020317	0.022034
**90**	0.019617	0.008766	**408**	0.022354	0.023206	**726**	0.020317	0.022034

**91**	0.019617	0.008766	**409**	0.022354	0.023206	**727**	0.020317	0.022034
**92**	0.019617	0.008766	**410**	0.022354	0.023206	**728**	0.020317	0.022034
**93**	0.019617	0.008766	**411**	0.022354	0.023206	**729**	0.020317	0.022034
**94**	0.019617	0.008766	**412**	0.022354	0.023206	**730**	0.020317	0.022034
**95**	0.019617	0.008766	**413**	0.022354	0.023206	**731**	0.020317	0.022034
**96**	0.019617	0.008766	**414**	0.022354	0.023206	**732**	0.020317	0.022034
**97**	0.019617	0.008766	**415**	0.022354	0.023206	**733**	0.020317	0.022034
**98**	0.019617	0.008766	**416**	0.022354	0.023206	**734**	0.020317	0.022034
**99**	0.019617	0.008766	**417**	0.022354	0.023206	**735**	0.020317	0.022034
**100**	0.018013	0.008766	**418**	0.022354	0.023206	**736**	0.020317	0.022034
**101**	0.018013	0.008766	**419**	0.022354	0.023206	**737**	0.020317	0.022034
**102**	0.018013	0.008766	**420**	0.022354	0.023206	**738**	0.020317	0.022034
**103**	0.018013	0.008766	**421**	0.022354	0.023206	**739**	0.020317	0.022034
**104**	0.018013	0.008766	**422**	0.022354	0.023206	**740**	0.020317	0.022034
**105**	0.018013	0.008766	**423**	0.022354	0.023206	**741**	0.020317	0.022034
**106**	0.018013	0.008766	**424**	0.022354	0.023206	**742**	0.020317	0.022034
**107**	0.018013	0.008766	**425**	0.022354	0.023206	**743**	0.020317	0.022034
**108**	0.018013	0.008766	**426**	0.022354	0.023206	**744**	0.020317	0.022034
**109**	0.018013	0.008766	**427**	0.022354	0.023206	**745**	0.020317	0.022034
**110**	0.018013	0.008766	**428**	0.022354	0.023206	**746**	0.020317	0.022034
**111**	0.018013	0.008766	**429**	0.022354	0.023206	**747**	0.020317	0.022034
**112**	0.018013	0.008766	**430**	0.022354	0.023206	**748**	0.020317	0.022034
**113**	0.018013	0.008766	**431**	0.022354	0.023206	**749**	0.020317	0.022034
**114**	0.018013	0.008766	**432**	0.022354	0.023206	**750**	0.020317	0.022034
**115**	0.018013	0.008766	**433**	0.022354	0.023206	**751**	0.020317	0.022034
**116**	0.018013	0.008766	**434**	0.022354	0.023206	**752**	0.020317	0.022034
**117**	0.018013	0.008766	**435**	0.022354	0.023206	**753**	0.020317	0.022034
**118**	0.018013	0.008766	**436**	0.022354	0.023206	**754**	0.020317	0.022034
**119**	0.018013	0.008766	**437**	0.022354	0.023206	**755**	0.020317	0.022034
**120**	0.018013	0.008766	**438**	0.022354	0.023206	**756**	0.020317	0.022034
**121**	0.018013	0.008766	**439**	0.022354	0.023206	**757**	0.020317	0.022034
**122**	0.018013	0.008766	**440**	0.022354	0.023206	**758**	0.020317	0.022034
**123**	0.018013	0.008766	**441**	0.022354	0.023206	**759**	0.020317	0.022034
**124**	0.018013	0.008766	**442**	0.022354	0.023206	**760**	0.020317	0.022034
**125**	0.018013	0.008766	**443**	0.022354	0.023206	**761**	0.020317	0.022034
**126**	0.018013	0.008766	**444**	0.022354	0.023206	**762**	0.020317	0.022034
**127**	0.018013	0.008766	**445**	0.022354	0.023206	**763**	0.020317	0.022034
**128**	0.018013	0.008766	**446**	0.022354	0.023206	**764**	0.020317	0.022034
**129**	0.018013	0.008766	**447**	0.022354	0.023206	**765**	0.020317	0.022034
**130**	0.018013	0.008766	**448**	0.022354	0.023206	**766**	0.020317	0.022034
**131**	0.018013	0.008766	**449**	0.022354	0.023206	**767**	0.020317	0.022034
**132**	0.018013	0.008766	**450**	0.022354	0.023206	**768**	0.020317	0.022034
**133**	0.018013	0.008766	**451**	0.022354	0.023206	**769**	0.020317	0.022034
**134**	0.018013	0.008766	**452**	0.022354	0.023206	**770**	0.020317	0.022034
**135**	0.01672	0.008766	**453**	0.022354	0.023206	**771**	0.020317	0.022034
**136**	0.01672	0.008766	**454**	0.022354	0.023206	**772**	0.020317	0.022034

**137**	0.01672	0.008766	**455**	0.022354	0.023206	**773**	0.020317	0.022034
**138**	0.01672	0.008766	**456**	0.022354	0.023206	**774**	0.020317	0.022034
**139**	0.01672	0.008766	**457**	0.022354	0.023206	**775**	0.020317	0.022034
**140**	0.01672	0.008766	**458**	0.022354	0.023206	**776**	0.020317	0.022034
**141**	0.01672	0.008766	**459**	0.022354	0.023206	**777**	0.020317	0.022034
**142**	0.01672	0.008766	**460**	0.022354	0.023206	**778**	0.020317	0.022034
**143**	0.01672	0.008766	**461**	0.022354	0.023206	**779**	0.020317	0.022034
**144**	0.01672	0.008766	**462**	0.022354	0.023206	**780**	0.020317	0.022034
**145**	0.01672	0.008766	**463**	0.022354	0.023206	**781**	0.020317	0.022034
**146**	0.01672	0.008766	**464**	0.022354	0.023206	**782**	0.020317	0.022034
**147**	0.01672	0.008766	**465**	0.022354	0.023206	**783**	0.020317	0.022034
**148**	0.01672	0.008766	**466**	0.022354	0.023206	**784**	0.020317	0.022034
**149**	0.01672	0.008766	**467**	0.022354	0.023206	**785**	0.020317	0.022034
**150**	0.01672	0.008766	**468**	0.022354	0.023206	**786**	0.020317	0.022034
**151**	0.01672	0.008766	**469**	0.022354	0.023206	**787**	0.020317	0.022034
**152**	0.01672	0.008766	**470**	0.022354	0.023206	**788**	0.020317	0.022034
**153**	0.01672	0.008766	**471**	0.022354	0.023206	**789**	0.020317	0.022034
**154**	0.01672	0.008766	**472**	0.022354	0.023206	**790**	0.020317	0.022034
**155**	0.01672	0.008766	**473**	0.022354	0.023206	**791**	0.020317	0.022034
**156**	0.01672	0.008766	**474**	0.022354	0.023206	**792**	0.020317	0.022034
**157**	0.01672	0.008766	**475**	0.022354	0.023206	**793**	0.020317	0.022034
**158**	0.01672	0.008766	**476**	0.022354	0.023206	**794**	0.020317	0.022034
**159**	0.01672	0.008766	**477**	0.022354	0.023206	**795**	0.020317	0.022034
**160**	0.01672	0.008766	**478**	0.022354	0.023206	**796**	0.020317	0.022034
**161**	0.01672	0.008766	**479**	0.022354	0.02229	**797**	0.020317	0.022034
**162**	0.01672	0.008766	**480**	0.022354	0.02229	**798**	0.020317	0.022034
**163**	0.01672	0.008766	**481**	0.022354	0.02229	**799**	0.020317	0.022034
**164**	0.01672	0.008766	**482**	0.022354	0.02229	**800**	0.020317	0.022034
**165**	0.01672	0.008766	**483**	0.022354	0.02229	**801**	0.020317	0.022034
**166**	0.01672	0.008766	**484**	0.022354	0.02229	**802**	0.020317	0.022034
**167**	0.01672	0.008766	**485**	0.022354	0.02229	**803**	0.020317	0.022034
**168**	0.01672	0.008766	**486**	0.022354	0.02229	**804**	0.020317	0.022034
**169**	0.01672	0.008766	**487**	0.022354	0.02229	**805**	0.020317	0.022034
**170**	0.01672	0.008766	**488**	0.022354	0.02229	**806**	0.020317	0.022034
**171**	0.01672	0.008766	**489**	0.022354	0.02229	**807**	0.020317	0.022034
**172**	0.01672	0.008766	**490**	0.022354	0.02229	**808**	0.020317	0.022034
**173**	0.01672	0.008766	**491**	0.022354	0.02229	**809**	0.020317	0.022034
**174**	0.01672	0.008766	**492**	0.022354	0.02229	**810**	0.020317	0.022034
**175**	0.01672	0.008766	**493**	0.022354	0.02229	**811**	0.020317	0.022034
**176**	0.01672	0.008766	**494**	0.022354	0.02229	**812**	0.020317	0.022034
**177**	0.01672	0.008766	**495**	0.022354	0.02229	**813**	0.020317	0.021901
**178**	0.01672	0.008766	**496**	0.022354	0.02229	**814**	0.020317	0.021901
**179**	0.01672	0.008766	**497**	0.022354	0.02229	**815**	0.020317	0.021901
**180**	0.01672	0.008766	**498**	0.022354	0.02229	**816**	0.020317	0.021901
**181**	0.01672	0.008766	**499**	0.022354	0.02229	**817**	0.020317	0.021901
**182**	0.01672	0.008766	**500**	0.022354	0.02229	**818**	0.020317	0.021901

**183**	0.01672	0.008766	**501**	0.022354	0.02229	**819**	0.020317	0.021901
**184**	0.01672	0.008766	**502**	0.022354	0.02229	**820**	0.020317	0.021901
**185**	0.01672	0.008766	**503**	0.022354	0.02229	**821**	0.020317	0.021901
**186**	0.01672	0.008766	**504**	0.022354	0.02229	**822**	0.020317	0.021901
**187**	0.01672	0.008766	**505**	0.022354	0.02229	**823**	0.020317	0.021901
**188**	0.01672	0.008766	**506**	0.022354	0.02229	**824**	0.020317	0.021901
**189**	0.01672	0.008766	**507**	0.022354	0.02229	**825**	0.020317	0.021901
**190**	0.01672	0.008766	**508**	0.022354	0.02229	**826**	0.020317	0.021901
**191**	0.01672	0.008766	**509**	0.022354	0.02229	**827**	0.020317	0.021901
**192**	0.01672	0.008766	**510**	0.022354	0.02229	**828**	0.020317	0.021901
**193**	0.01672	0.008766	**511**	0.022354	0.02229	**829**	0.020317	0.021901
**194**	0.01672	0.008766	**512**	0.022354	0.02229	**830**	0.020317	0.021901
**195**	0.01672	0.008766	**513**	0.022354	0.02229	**831**	0.020317	0.021901
**196**	0.01672	0.008766	**514**	0.022354	0.02229	**832**	0.020317	0.021901
**197**	0.01672	0.008766	**515**	0.022354	0.02229	**833**	0.020317	0.021901
**198**	0.01672	0.008766	**516**	0.022354	0.02229	**834**	0.020317	0.021901
**199**	0.01672	0.008766	**517**	0.022354	0.02229	**835**	0.020317	0.021901
**200**	0.01672	0.008766	**518**	0.022354	0.02229	**836**	0.020317	0.021901
**201**	0.01672	0.008766	**519**	0.022354	0.02229	**837**	0.020317	0.021901
**202**	0.01672	0.008766	**520**	0.021297	0.02229	**838**	0.020317	0.021901
**203**	0.01672	0.008766	**521**	0.021297	0.02229	**839**	0.020317	0.021901
**204**	0.01672	0.008766	**522**	0.021297	0.02229	**840**	0.020317	0.021901
**205**	0.01672	0.008766	**523**	0.021297	0.02229	**841**	0.020317	0.021901
**206**	0.01672	0.008766	**524**	0.021297	0.02229	**842**	0.020317	0.021901
**207**	0.01672	0.008766	**525**	0.021297	0.02229	**843**	0.020317	0.021901
**208**	0.01672	0.008766	**526**	0.021297	0.02229	**844**	0.020317	0.021901
**209**	0.01672	0.008766	**527**	0.021297	0.02229	**845**	0.020317	0.021901
**210**	0.01672	0.008766	**528**	0.021297	0.02229	**846**	0.020317	0.021901
**211**	0.01672	0.008766	**529**	0.021297	0.02229	**847**	0.020317	0.021901
**212**	0.01672	0.008766	**530**	0.021297	0.02229	**848**	0.020317	0.021901
**213**	0.013725	0.008766	**531**	0.021297	0.02229	**849**	0.020317	0.021901
**214**	0.013725	0.008766	**532**	0.021297	0.02229	**850**	0.020317	0.021901
**215**	0.013725	0.008766	**533**	0.021297	0.02229	**851**	0.020317	0.021901
**216**	0.013725	0.008766	**534**	0.021297	0.02229	**852**	0.020317	0.021901
**217**	0.013725	0.008766	**535**	0.021297	0.02229	**853**	0.020317	0.021901
**218**	0.013725	0.008766	**536**	0.021297	0.02229	**854**	0.020317	0.021901
**219**	0.013725	0.008766	**537**	0.021297	0.02229	**855**	0.020317	0.021901
**220**	0.013725	0.008766	**538**	0.021297	0.02229	**856**	0.020317	0.021901
**221**	0.013725	0.008766	**539**	0.021297	0.02229	**857**	0.020317	0.021901
**222**	0.013725	0.008766	**540**	0.021297	0.02229	**858**	0.020317	0.021901
**223**	0.013725	0.008766	**541**	0.021297	0.02229	**859**	0.020317	0.021901
**224**	0.013725	0.008766	**542**	0.021297	0.02229	**860**	0.020317	0.021901
**225**	0.013725	0.008766	**543**	0.021297	0.02229	**861**	0.020317	0.021901
**226**	0.013725	0.00786	**544**	0.021297	0.02229	**862**	0.020317	0.021901
**227**	0.013725	0.00786	**545**	0.021297	0.02229	**863**	0.020317	0.021901
**228**	0.013725	0.00786	**546**	0.021297	0.02229	**864**	0.020317	0.021901

**229**	0.013725	0.00786	**547**	0.021297	0.02229	**865**	0.020317	0.021901
**230**	0.013725	0.00786	**548**	0.021297	0.02229	**866**	0.020317	0.021901
**231**	0.013725	0.00786	**549**	0.021297	0.02229	**867**	0.020317	0.021901
**232**	0.013725	0.00786	**550**	0.021297	0.02229	**868**	0.020317	0.021901
**233**	0.013725	0.00786	**551**	0.021297	0.02229	**869**	0.020317	0.021901
**234**	0.013725	0.00786	**552**	0.021297	0.02229	**870**	0.020317	0.021901
**235**	0.013725	0.00786	**553**	0.021297	0.02229	**871**	0.020317	0.021901
**236**	0.013725	0.00786	**554**	0.021297	0.02229	**872**	0.020317	0.021901
**237**	0.013725	0.00786	**555**	0.021297	0.02229	**873**	0.020317	0.021901
**238**	0.013725	0.00786	**556**	0.021297	0.02229	**874**	0.018707	0.021901
**239**	0.013725	0.00786	**557**	0.021297	0.02229	**875**	0.018707	0.021901
**240**	0.013725	0.00786	**558**	0.021297	0.02229	**876**	0.018707	0.021901
**241**	0.013725	0.00786	**559**	0.021297	0.02229	**877**	0.018707	0.021901
**242**	0.013725	0.00786	**560**	0.021297	0.02229	**878**	0.018707	0.021901
**243**	0.013725	0.00786	**561**	0.021297	0.02229	**879**	0.018707	0.021901
**244**	0.013725	0.00786	**562**	0.021297	0.02229	**880**	0.018707	0.021901
**245**	0.013725	0.00786	**563**	0.021297	0.02229	**881**	0.018707	0.021901
**246**	0.013725	0.00786	**564**	0.021297	0.02229	**882**	0.018707	0.021901
**247**	0.013725	0.00786	**565**	0.021297	0.02229	**883**	0.018707	0.021901
**248**	0.013725	0.00786	**566**	0.021297	0.02229	**884**	0.018707	0.021901
**249**	0.013725	0.00786	**567**	0.021297	0.02229	**885**	0.018707	0.021901
**250**	0.013725	0.00786	**568**	0.021297	0.02229	**886**	0.018707	0.021901
**251**	0.013725	0.00786	**569**	0.021297	0.02229	**887**	0.018707	0.021901
**252**	0.013725	0.00786	**570**	0.021297	0.02229	**888**	0.018707	0.021901
**253**	0.013725	0.00786	**571**	0.021297	0.02229	**889**	0.018707	0.021901
**254**	0.013725	0.00786	**572**	0.021297	0.02229	**890**	0.018707	0.021901
**255**	0.013725	0.00786	**573**	0.021297	0.02229	**891**	0.018707	0.021901
**256**	0.013725	0.00786	**574**	0.021297	0.02229	**892**	0.018707	0.021901
**257**	0.013725	0.00786	**575**	0.021297	0.02229	**893**	0.018707	0.021901
**258**	0.013725	0.00786	**576**	0.021297	0.02229	**894**	0.018707	0.021901
**259**	0.013725	0.00786	**577**	0.021297	0.02229	**895**	0.018707	0.021901
**260**	0.013725	0.00786	**578**	0.021297	0.02229	**896**	0.018707	0.021901
**261**	0.013725	0.00786	**579**	0.020317	0.02229	**897**	0.018707	0.021901
**262**	0.013725	0.00786	**580**	0.020317	0.02229	**898**	0.018707	0.021901
**263**	0.013725	0.00786	**581**	0.020317	0.02229	**899**	0.018707	0.021901
**264**	0.013725	0.00786	**582**	0.020317	0.02229	**900**	0.018707	0.021901
**265**	0.011895	0.00786	**583**	0.020317	0.02229	**901**	0.018707	0.021901
**266**	0.011895	0.00786	**584**	0.020317	0.02229	**902**	0.018707	0.021901
**267**	0.011608	0.00786	**585**	0.020317	0.02229	**903**	0.018707	0.021901
**268**	0.011608	0.00786	**586**	0.020317	0.02229	**904**	0.018707	0.021901
**269**	0.011608	0.00786	**587**	0.020317	0.02229	**905**	0.018707	0.021901
**270**	0.011608	0.00786	**588**	0.020317	0.02229	**906**	0.018707	0.021901
**271**	0.011608	0.00786	**589**	0.020317	0.02229	**907**	0.018707	0.021901
**272**	0.011608	0.00786	**590**	0.020317	0.02229	**908**	0.018707	0.021901
**273**	0.011608	0.00786	**591**	0.020317	0.02229	**909**	0.018707	0.021901
**274**	0.011608	0.00786	**592**	0.020317	0.02229	**910**	0.018707	0.021901

**275**	0.011608	0.00786	**593**	0.020317	0.02229	**911**	0.018707	0.021901
**276**	0.011608	0.00786	**594**	0.020317	0.02229	**912**	0.018707	0.021901
**277**	0.011608	0.00786	**595**	0.020317	0.02229	**913**	0.018707	0.021901
**278**	0.011608	0.00786	**596**	0.020317	0.02229	**914**	0.018707	0.021901
**279**	0.011608	0.00786	**597**	0.020317	0.02229	**915**	0.018707	0.021901
**280**	0.011608	0.00786	**598**	0.020317	0.02229	**916**	0.018697	0.021901
**281**	0.011608	0.00786	**599**	0.020317	0.02229	**917**	0.018697	0.021901
**282**	0.011608	0.00786	**600**	0.020317	0.02229	**918**	0.018697	0.021901
**283**	0.011608	0.00786	**601**	0.020317	0.02229	**919**	0.018697	0.021901
**284**	0.011608	0.00786	**602**	0.020317	0.02229	**920**	0.018697	0.021901
**285**	0.011608	0.00786	**603**	0.020317	0.02229	**921**	0.018697	0.021901
**286**	0.011608	0.00786	**604**	0.020317	0.02229	**922**	0.018697	0.021901
**287**	0.011608	0.00786	**605**	0.020317	0.02229	**923**	0.018697	0.021901
**288**	0.011608	0.00786	**606**	0.020317	0.02229	**924**	0.018697	0.021901
**289**	0.011608	0.00786	**607**	0.020317	0.02229	**925**	0.018697	0.021901
**290**	0.011608	0.00786	**608**	0.020317	0.02229	**926**	0.018697	0.021901
**291**	0.011608	0.00786	**609**	0.020317	0.02229	**927**	0.018697	0.021901
**292**	0.011608	0.00786	**610**	0.020317	0.02229	**928**	0.018697	0.021901
**293**	0.011608	0.00786	**611**	0.020317	0.022034	**929**	0.018697	0.021901
**294**	0.011608	0.00786	**612**	0.020317	0.022034	**930**	0.018697	0.021901
**295**	0.011608	0.00786	**613**	0.020317	0.022034	**931**	0.018697	0.021901
**296**	0.011608	0.00786	**614**	0.020317	0.022034	**932**	0.018697	0.021901
**297**	0.011608	0.00786	**615**	0.020317	0.022034	**933**	0.018697	0.021901
**298**	0.011608	0.00786	**616**	0.020317	0.022034	**934**	0.018697	0.021901
**299**	0.011608	0.00786	**617**	0.020317	0.022034	**935**	0.018697	0.021901
**300**	0.011608	0.00786	**618**	0.020317	0.022034	**936**	0.018697	0.021901
**301**	0.011608	0.00786	**619**	0.020317	0.022034	**937**	0.018697	0.021901
**302**	0.011608	0.00786	**620**	0.020317	0.022034	**938**	0.018697	0.021901
**303**	0.011608	0.006154	**621**	0.020317	0.022034	**939**	0.018697	0.021901
**304**	0.011608	0.006154	**622**	0.020317	0.022034	**940**	0.018697	0.021901
**305**	0.011608	0.006154	**623**	0.020317	0.022034	**941**	0.018697	0.021901
**306**	0.011608	0.006154	**624**	0.020317	0.022034	**942**	0.018697	0.021901
**307**	0.011608	0.006154	**625**	0.020317	0.022034	**943**	0.018697	0.021901
**308**	0.011608	0.006154	**626**	0.020317	0.022034	**944**	0.018697	0.021901
**309**	0.011608	0.006154	**627**	0.020317	0.022034	**945**	0.018697	0.021901
**310**	0.011608	0.006154	**628**	0.020317	0.022034	**946**	0.018697	0.021901
**311**	0.011608	0.006154	**629**	0.020317	0.022034	**947**	0.018697	0.021901
**312**	0.011608	0.006154	**630**	0.020317	0.022034	**948**	0.018697	0.021901
**313**	0.011608	0.006154	**631**	0.020317	0.022034	**949**	0.018697	0.021901
**314**	0.011608	0.006154	**632**	0.020317	0.022034	**950**	0.017928	0.021901
**315**	0.011608	0.006154	**633**	0.020317	0.022034			
**316**	0.011608	0.006154	**634**	0.020317	0.022034			
**317**	0.011608	0.006154	**635**	0.020317	0.022034			
**318**	0.011608	0.006154	**636**	0.020317	0.022034			

Table 20. Convergence of the third damage scenario in the presence of 5% noise for first 1 and 3 modes

Iterations	1 Mode	3 Modes	Iterations	1 Modes	3 Modes	Iterations	1 Modes	3 Modes
**1**	0.29387	0.27324	**137**	0.003579	0.001751	**637**	0.011608	0.003763
**2**	0.21728	0.20852	**138**	0.003579	0.001751	**638**	0.011608	0.003763
**3**	0.15174	0.15454	**139**	0.003579	0.001477	**639**	0.011608	0.003763
**4**	0.12417	0.10902	**140**	0.003579	0.001477	**640**	0.011608	0.003763
**5**	0.11206	0.1029	**141**	0.003579	0.001477	**641**	0.011608	0.003763
**6**	0.091515	0.095722	**142**	0.003579	0.001477	**642**	0.011608	0.003763
**7**	0.091515	0.084705	**143**	0.003579	0.001477	**643**	0.011608	0.003763
**8**	0.058718	0.072416	**144**	0.003579	0.001477	**644**	0.011608	0.003763
**9**	0.058718	0.06211	**145**	0.003579	0.001477	**645**	0.011608	0.003763
**10**	0.058718	0.049497	**146**	0.003579	0.001477	**646**	0.011608	0.003763
**11**	0.058718	0.042026	**147**	0.003441	0.001477	**647**	0.011608	0.003763
**12**	0.05238	0.042026	**148**	0.003441	0.001477	**648**	0.011608	0.003763
**13**	0.05238	0.042026	**149**	0.003441	0.001477	**649**	0.011608	0.003763
**14**	0.05238	0.042026	**150**	0.003441	0.001477	**650**	0.011608	0.003763
**15**	0.05238	0.03619	**151**	0.002817	0.001477	**651**	0.011608	0.003763
**16**	0.051993	0.03619	**152**	0.002817	0.001477	**652**	0.011608	0.003763
**17**	0.051993	0.031836	**153**	0.002817	0.001477	**653**	0.011608	0.003763
**18**	0.051993	0.029643	**154**	0.002817	0.001477	**654**	0.011608	0.003763
**19**	0.051993	0.029643	**155**	0.002817	0.001477	**655**	0.011608	0.003763
**20**	0.051993	0.029643	**156**	0.002817	0.001477	**656**	0.011608	0.003763
**21**	0.051993	0.029643	**157**	0.002817	0.001477	**657**	0.011608	0.003763
**22**	0.051993	0.029643	**158**	0.002817	0.001441	**658**	0.011608	0.003763
**23**	0.051993	0.029643	**159**	0.002817	0.001441	**659**	0.011608	0.003763
**24**	0.051993	0.028287	**160**	0.002817	0.001368	**660**	0.011608	0.003763
**25**	0.051993	0.028287	**161**	0.002817	0.001368	**661**	0.011608	0.003763
**26**	0.049006	0.026025	**162**	0.002817	0.00133	**662**	0.011608	0.003763
**27**	0.049006	0.026025	**163**	0.002817	0.00133	**663**	0.011608	0.003763
**28**	0.049006	0.026025	**164**	0.002817	0.00133	**664**	0.011608	0.003763
**29**	0.049006	0.026025	**165**	0.002817	0.00133	**665**	0.011608	0.003763
**30**	0.049006	0.026025	**166**	0.002817	0.00133	**666**	0.011608	0.003763
**31**	0.049006	0.026025	**167**	0.002817	0.00133	**667**	0.011608	0.003763
**32**	0.049006	0.026025	**168**	0.002817	0.00133	**668**	0.011608	0.003763
**33**	0.049006	0.026025	**169**	0.002817	0.00133	**669**	0.011608	0.003763
**34**	0.049006	0.026025	**170**	0.002817	0.00133	**670**	0.011608	0.003763
**35**	0.049006	0.026025	**171**	0.002817	0.001322	**671**	0.011608	0.003763
**36**	0.049006	0.026025	**172**	0.002817	0.001322	**672**	0.011608	0.003763
**37**	0.049006	0.026025	**173**	0.002322	0.001318	**673**	0.011145	0.003763
**38**	0.049006	0.026025	**174**	0.002322	0.001318	**674**	0.011145	0.003763
**39**	0.049006	0.026025	**175**	0.002322	0.001318	**675**	0.00931	0.003763
**40**	0.049006	0.026025	**176**	0.002322	0.001318	**676**	0.00931	0.003763
**41**	0.049006	0.026025	**177**	0.002322	0.001318	**677**	0.00931	0.003763
**42**	0.049006	0.026025	**178**	0.002322	0.001318	**678**	0.00931	0.003763

**43**	0.049006	0.026025	**179**	0.002322	0.001148	**679**	0.00931	0.003763
**44**	0.049006	0.026025	**180**	0.002322	0.001148	**680**	0.00931	0.003763
**45**	0.049006	0.026025	**181**	0.002322	0.001148	**681**	0.00931	0.003763
**46**	0.049006	0.026025	**182**	0.002322	0.001148	**682**	0.00931	0.003763
**47**	0.049006	0.026025	**183**	0.002322	0.001148	**683**	0.00931	0.003763
**48**	0.049006	0.026025	**184**	0.002322	0.001148	**684**	0.00931	0.003763
**49**	0.049006	0.026025	**185**	0.002322	0.001146	**685**	0.00931	0.003763
**50**	0.049006	0.026025	**186**	0.002322	0.001146	**686**	0.00931	0.003763
**51**	0.049006	0.026025	**187**	0.002322	0.001146	**687**	0.00931	0.003763
**52**	0.049006	0.026025	**188**	0.002322	0.001146	**688**	0.00931	0.003763
**53**	0.049006	0.026025	**189**	0.002322	0.001146	**689**	0.00931	0.003763
**54**	0.049006	0.026025	**190**	0.002322	0.001146	**690**	0.00931	0.003763
**55**	0.047219	0.026025	**191**	0.002322	0.001146	**691**	0.00931	0.003763
**56**	0.047219	0.026025	**192**	0.002322	0.001146	**692**	0.00931	0.003763
**57**	0.047219	0.026025	**193**	0.002322	0.000987	**693**	0.00931	0.003763
**58**	0.047219	0.026025	**194**	0.002322	0.000987	**694**	0.00931	0.003763
**59**	0.047219	0.026025	**195**	0.002322	0.000987	**695**	0.00931	0.003763
**60**	0.047219	0.026025	**196**	0.002322	0.000987	**696**	0.00931	0.003763
**61**	0.047219	0.026025	**197**	0.002149	0.000987	**697**	0.00931	0.003763
**62**	0.047219	0.026025	**198**	0.002149	0.000987	**698**	0.00931	0.003763
**63**	0.047219	0.026025	**199**	0.002149	0.000987	**699**	0.00931	0.003763
**64**	0.047219	0.026025	**200**	0.002149	0.000987	**700**	0.00931	0.003763
**65**	0.047219	0.026025	**201**	0.002149	0.000987	**701**	0.00931	0.002801
**66**	0.047219	0.026025	**202**	0.002149	0.000987	**702**	0.00931	0.002801
**67**	0.047219	0.026025	**203**	0.002149	0.000987	**703**	0.00931	0.002801
**68**	0.047219	0.026025	**204**	0.002149	0.000983	**704**	0.00931	0.002801
**69**	0.047219	0.026025	**205**	0.002149	0.000983	**705**	0.00931	0.002801
**70**	0.047219	0.026025	**206**	0.002149	0.000983	**706**	0.00931	0.002801
**71**	0.047219	0.026025	**207**	0.002149	0.000983	**707**	0.00931	0.002801
**72**	0.047219	0.026025	**208**	0.002004	0.000964	**708**	0.00931	0.002801
**73**	0.047219	0.026025	**209**	0.002004	0.000943	**709**	0.00931	0.002801
**74**	0.047219	0.026025	**210**	0.002004	0.000943	**710**	0.00931	0.002801
**75**	0.047219	0.026025	**211**	0.002004	0.000921	**711**	0.00931	0.002801
**76**	0.047219	0.026025	**212**	0.002004	0.000921	**712**	0.00931	0.002801
**77**	0.041814	0.024273	**213**	0.002004	0.000921	**713**	0.00931	0.002801
**78**	0.041814	0.024273	**214**	0.002004	0.000921	**714**	0.00931	0.002801
**79**	0.041814	0.024273	**215**	0.002004	0.000921	**715**	0.00931	0.002801
**80**	0.041814	0.024273	**216**	0.002004	0.000921	**716**	0.00931	0.002801
**81**	0.041814	0.024273	**217**	0.002004	0.000921	**717**	0.00931	0.002801
**82**	0.041556	0.024273	**218**	0.002004	0.000915	**718**	0.00931	0.002801
**83**	0.041556	0.024273	**219**	0.002004	0.000915	**719**	0.00931	0.002801
**84**	0.041556	0.024273	**220**	0.002004	0.000915	**720**	0.00931	0.002801
**85**	0.041556	0.024273	**221**	0.002004	0.000845	**721**	0.00931	0.002801
**86**	0.041556	0.024273	**222**	0.002004	0.000845	**722**	0.00931	0.002801
**87**	0.041556	0.024273	**223**	0.002004	0.000845	**723**	0.00931	0.002801
**88**	0.041556	0.024273	**224**	0.002004	0.000845	**724**	0.00931	0.002801

**89**	0.041556	0.024273	**225**	0.002004	0.00084	**725**	0.00931	0.002801
**90**	0.041556	0.024273	**226**	0.002004	0.00084	**726**	0.00931	0.002801
**91**	0.041556	0.024273	**227**	0.002004	0.00084	**727**	0.00931	0.002801
**92**	0.041556	0.024273	**228**	0.002004	0.00084	**728**	0.00931	0.002801
**93**	0.041556	0.024273	**229**	0.002004	0.00084	**729**	0.00931	0.002801
**94**	0.041556	0.024273	**230**	0.002004	0.00084	**730**	0.00931	0.002801
**95**	0.041556	0.024273	**231**	0.002004	0.00084	**731**	0.00931	0.002801
**96**	0.039698	0.024273	**232**	0.002004	0.000819	**732**	0.00931	0.002801
**97**	0.039698	0.024273	**233**	0.002004	0.000753	**733**	0.00931	0.002801
**98**	0.039698	0.024273	**234**	0.002004	0.000753	**734**	0.00931	0.002801
**99**	0.035714	0.024273	**235**	0.002004	0.000753	**735**	0.00931	0.002801
**100**	0.035714	0.024273	**236**	0.002004	0.000753	**736**	0.00931	0.002801
**101**	0.035714	0.024273	**237**	0.002004	0.000753	**737**	0.00931	0.002801
**102**	0.035714	0.024273	**238**	0.002004	0.000753	**738**	0.00931	0.002801
**103**	0.035714	0.024273	**239**	0.002004	0.000753	**739**	0.00931	0.002801
**104**	0.035714	0.024273	**240**	0.002004	0.000753	**740**	0.00931	0.002801
**105**	0.035714	0.024273	**241**	0.002004	0.000737	**741**	0.00931	0.002801
**106**	0.035714	0.024273	**242**	0.002004	0.000737	**742**	0.00931	0.002801
**107**	0.035714	0.024273	**243**	0.002004	0.000731	**743**	0.00931	0.002801
**108**	0.035714	0.024273	**244**	0.002004	0.000731	**744**	0.00931	0.002801
**109**	0.035714	0.024273	**245**	0.001938	0.000731	**745**	0.00931	0.002801
**110**	0.035714	0.024273	**246**	0.001938	0.000731	**746**	0.00931	0.002801
**111**	0.035714	0.024273	**247**	0.001938	0.000731	**747**	0.00931	0.002801
**112**	0.035714	0.024273	**248**	0.001938	0.000731	**748**	0.00931	0.002801
**113**	0.035714	0.024273	**249**	0.001938	0.000731	**749**	0.00931	0.002801
**114**	0.035714	0.024273	**250**	0.001938	0.000731	**750**	0.00931	0.002801
**115**	0.035714	0.024273	**251**	0.001938	0.00072	**751**	0.00931	0.002801
**116**	0.035714	0.024273	**252**	0.001938	0.00072	**752**	0.00931	0.002801
**117**	0.035714	0.024273	**253**	0.001938	0.00072	**753**	0.00931	0.002801
**118**	0.035714	0.024273	**254**	0.001871	0.00072	**754**	0.00931	0.002801
**119**	0.035714	0.024273	**255**	0.001871	0.00072	**755**	0.00931	0.002801
**120**	0.035714	0.024273	**256**	0.001871	0.00072	**756**	0.00931	0.002801
**121**	0.035714	0.024273	**257**	0.001871	0.00072	**757**	0.00931	0.002801
**122**	0.034946	0.024273	**258**	0.001871	0.00072	**758**	0.00931	0.002801
**123**	0.034946	0.024273	**259**	0.001871	0.00072	**759**	0.00931	0.002801
**124**	0.034946	0.024273	**260**	0.001871	0.000717	**760**	0.00931	0.002801
**125**	0.034946	0.024273	**261**	0.001871	0.000717	**761**	0.00931	0.002801
**126**	0.034946	0.024273	**262**	0.001871	0.000717	**762**	0.00931	0.002801
**127**	0.034946	0.024273	**263**	0.001871	0.000717	**763**	0.00931	0.002801
**128**	0.034946	0.024273	**264**	0.00184	0.000706	**764**	0.00931	0.002801
**129**	0.031671	0.024273	**265**	0.00184	0.000706	**765**	0.00931	0.002801
**130**	0.031671	0.024273	**266**	0.00184	0.000701	**766**	0.00931	0.002801
**131**	0.031671	0.024273	**267**	0.001804	0.000674	**767**	0.00931	0.002801
**132**	0.031671	0.024273	**268**	0.001804	0.000663	**768**	0.00931	0.002801
**133**	0.031671	0.024273	**269**	0.001804	0.000663	**769**	0.00931	0.002801
**134**	0.031671	0.024273	**270**	0.001804	0.000659	**770**	0.00931	0.002801

**135**	0.031671	0.024273	**271**	0.001804	0.000657	**771**	0.00931	0.002801
**136**	0.031671	0.024273	**272**	0.001804	0.000657	**772**	0.00931	0.002801
**137**	0.031671	0.024273	**273**	0.001804	0.000657	**773**	0.00931	0.002801
**138**	0.031671	0.024273	**274**	0.001804	0.000637	**774**	0.00931	0.002801
**139**	0.031671	0.024273	**275**	0.0018	0.000637	**775**	0.00931	0.002801
**140**	0.031671	0.024273	**276**	0.0018	0.000637	**776**	0.00931	0.002801
**141**	0.031671	0.024273	**277**	0.0018	0.000627	**777**	0.00931	0.002801
**142**	0.031263	0.024273	**278**	0.0018	0.000617	**778**	0.00931	0.002801
**143**	0.031263	0.024273	**279**	0.0018	0.000617	**779**	0.00931	0.002801
**144**	0.031263	0.024273	**280**	0.001789	0.000617	**780**	0.00931	0.002801
**145**	0.031263	0.024273	**281**	0.001789	0.000613	**781**	0.00931	0.002801
**146**	0.031263	0.024273	**282**	0.001789	0.000609	**782**	0.00931	0.002801
**147**	0.031263	0.024273	**283**	0.001771	0.000609	**783**	0.00931	0.002801
**148**	0.031263	0.024273	**284**	0.001771	0.000609	**784**	0.00931	0.002801
**149**	0.031263	0.024273	**285**	0.001771	0.000608	**785**	0.00931	0.002801
**150**	0.031263	0.024273	**286**	0.001771	0.000607	**786**	0.00931	0.002801
**151**	0.031263	0.024273	**287**	0.001771	0.000597	**787**	0.00931	0.002801
**152**	0.031263	0.024273	**288**	0.001771	0.000593	**788**	0.00931	0.002801
**153**	0.031263	0.024273	**289**	0.001738	0.000593	**789**	0.00931	0.002801
**154**	0.031263	0.024273	**290**	0.001738	0.000585	**790**	0.00931	0.002801
**155**	0.031263	0.024273	**291**	0.001738	0.000583	**791**	0.00931	0.002801
**156**	0.031263	0.024273	**292**	0.001738	0.000583	**792**	0.00931	0.002801
**157**	0.031263	0.024273	**293**	0.001738	0.000579	**793**	0.00931	0.002801
**158**	0.031263	0.024273	**294**	0.001732	0.000577	**794**	0.00931	0.002801
**159**	0.031263	0.024273	**295**	0.001729	0.000577	**795**	0.00931	0.002801
**160**	0.031263	0.024273	**296**	0.001729	0.000576	**796**	0.00931	0.002801
**161**	0.031263	0.024273	**297**	0.001729	0.000576	**797**	0.00931	0.002801
**162**	0.031263	0.024273	**298**	0.001729	0.000575	**798**	0.00931	0.002801
**163**	0.031263	0.024273	**299**	0.001729	0.000574	**799**	0.00931	0.002801
**164**	0.031263	0.024273	**300**	0.001725	0.000574	**800**	0.00931	0.002801
**165**	0.031263	0.024273	**137**	0.003579	0.001751	**801**	0.00931	0.002801
**166**	0.031263	0.024273	**138**	0.003579	0.001751	**802**	0.00931	0.002801
**167**	0.025361	0.024273	**139**	0.003579	0.001477	**803**	0.00931	0.002801
**168**	0.025361	0.024273	**140**	0.003579	0.001477	**804**	0.00931	0.002801
**169**	0.025361	0.024273	**141**	0.003579	0.001477	**805**	0.00931	0.002801
**170**	0.025361	0.024273	**142**	0.003579	0.001477	**806**	0.00931	0.002801
**171**	0.025361	0.024273	**143**	0.003579	0.001477	**807**	0.00931	0.002801
**172**	0.025361	0.024273	**144**	0.003579	0.001477	**808**	0.00931	0.002801
**173**	0.025361	0.024273	**145**	0.003579	0.001477	**809**	0.00931	0.002801
**174**	0.025361	0.024273	**146**	0.003579	0.001477	**810**	0.00931	0.002801
**175**	0.025361	0.024273	**147**	0.003441	0.001477	**811**	0.00931	0.002801
**176**	0.025361	0.024273	**148**	0.003441	0.001477	**812**	0.00931	0.002801
**177**	0.025361	0.024273	**149**	0.003441	0.001477	**813**	0.00931	0.002801
**178**	0.025361	0.024258	**150**	0.003441	0.001477	**814**	0.00931	0.002801
**179**	0.025361	0.024258	**151**	0.002817	0.001477	**815**	0.00931	0.002801
**180**	0.025361	0.024258	**152**	0.002817	0.001477	**816**	0.00931	0.002801

**181**	0.025361	0.024258	**153**	0.002817	0.001477	**817**	0.00931	0.002801
**182**	0.025361	0.024258	**154**	0.002817	0.001477	**818**	0.00931	0.002801
**183**	0.025361	0.024258	**155**	0.002817	0.001477	**819**	0.00931	0.002801
**184**	0.025361	0.024258	**156**	0.002817	0.001477	**820**	0.00931	0.002801
**185**	0.025361	0.024258	**157**	0.002817	0.001477	**821**	0.00931	0.002801
**186**	0.025361	0.024258	**158**	0.002817	0.001441	**822**	0.00931	0.002801
**187**	0.025361	0.024258	**159**	0.002817	0.001441	**823**	0.00931	0.002801
**188**	0.025361	0.024258	**160**	0.002817	0.001368	**824**	0.00931	0.002801
**189**	0.025361	0.024258	**161**	0.002817	0.001368	**825**	0.00931	0.002801
**190**	0.025361	0.024258	**162**	0.002817	0.00133	**826**	0.00931	0.002801
**191**	0.025361	0.024258	**163**	0.002817	0.00133	**827**	0.00931	0.002801
**192**	0.025361	0.024258	**164**	0.002817	0.00133	**828**	0.00931	0.002801
**193**	0.025361	0.024258	**165**	0.002817	0.00133	**829**	0.00931	0.002801
**194**	0.023863	0.024258	**166**	0.002817	0.00133	**830**	0.00931	0.002801
**195**	0.023863	0.024258	**167**	0.002817	0.00133	**831**	0.00931	0.002801
**196**	0.023863	0.024258	**168**	0.002817	0.00133	**832**	0.00931	0.002801
**197**	0.023863	0.024258	**169**	0.002817	0.00133	**833**	0.00931	0.002801
**198**	0.023863	0.024258	**170**	0.002817	0.00133	**834**	0.00931	0.002801
**199**	0.023863	0.024258	**171**	0.002817	0.001322	**835**	0.00931	0.002801
**200**	0.023863	0.024258	**172**	0.002817	0.001322	**836**	0.00931	0.002801
**201**	0.023863	0.024258	**173**	0.002322	0.001318	**837**	0.00931	0.002801
**202**	0.023863	0.024258	**174**	0.002322	0.001318	**838**	0.00931	0.002801
**203**	0.023863	0.024258	**175**	0.002322	0.001318	**839**	0.00931	0.002801
**204**	0.023863	0.024258	**176**	0.002322	0.001318	**840**	0.00931	0.002801
**205**	0.023863	0.024258	**177**	0.002322	0.001318	**841**	0.00931	0.002801
**206**	0.023863	0.024258	**178**	0.002322	0.001318	**842**	0.00931	0.002801
**207**	0.023863	0.024258	**179**	0.002322	0.001148	**843**	0.00931	0.002801
**208**	0.023863	0.024258	**180**	0.002322	0.001148	**844**	0.00931	0.002801
**209**	0.023863	0.024258	**181**	0.002322	0.001148	**845**	0.00931	0.002801
**210**	0.023863	0.024258	**182**	0.002322	0.001148	**846**	0.00931	0.002801
**211**	0.023863	0.024258	**183**	0.002322	0.001148	**847**	0.00931	0.002801
**212**	0.023863	0.024258	**184**	0.002322	0.001148	**848**	0.00931	0.002801
**213**	0.023863	0.024258	**185**	0.002322	0.001146	**849**	0.00931	0.002801
**214**	0.023863	0.024258	**186**	0.002322	0.001146	**850**	0.00931	0.002801
**215**	0.023863	0.024258	**187**	0.002322	0.001146	**851**	0.00931	0.002801
**216**	0.023863	0.024258	**188**	0.002322	0.001146	**852**	0.00931	0.002801
**217**	0.023863	0.024258	**189**	0.002322	0.001146	**853**	0.00931	0.002801
**218**	0.023863	0.024258	**190**	0.002322	0.001146	**854**	0.00931	0.002801
**219**	0.023863	0.024258	**191**	0.002322	0.001146	**855**	0.00931	0.002801
**220**	0.023863	0.024258	**192**	0.002322	0.001146	**856**	0.009295	0.002801
**221**	0.023789	0.024258	**193**	0.002322	0.000987	**857**	0.009295	0.002801
**222**	0.023789	0.024258	**194**	0.002322	0.000987	**858**	0.009295	0.002801
**223**	0.023789	0.024258	**195**	0.002322	0.000987	**859**	0.009295	0.002801
**224**	0.023789	0.024258	**196**	0.002322	0.000987	**860**	0.009295	0.002801
**225**	0.023789	0.024258	**197**	0.002149	0.000987	**861**	0.009295	0.002801
**226**	0.023789	0.024258	**198**	0.002149	0.000987	**862**	0.009295	0.002801

**227**	0.023789	0.024258	**199**	0.002149	0.000987	**863**	0.009295	0.002801
**228**	0.023789	0.024258	**200**	0.002149	0.000987	**864**	0.009295	0.002801
**229**	0.023789	0.024258	**201**	0.002149	0.000987	**865**	0.009295	0.002801
**230**	0.023789	0.024258	**202**	0.002149	0.000987	**866**	0.009295	0.002801
**231**	0.023789	0.024258	**203**	0.002149	0.000987	**867**	0.009295	0.002801
**232**	0.023789	0.024258	**204**	0.002149	0.000983	**868**	0.009295	0.002801
**233**	0.023789	0.024258	**205**	0.002149	0.000983	**869**	0.009295	0.002801
**234**	0.023789	0.024258	**206**	0.002149	0.000983	**870**	0.009295	0.002801
**235**	0.023789	0.024258	**207**	0.002149	0.000983	**871**	0.008846	0.002801
**236**	0.023789	0.024258	**208**	0.002004	0.000964	**872**	0.008846	0.002801
**237**	0.023789	0.024258	**209**	0.002004	0.000943	**873**	0.008846	0.002801
**238**	0.023789	0.024258	**210**	0.002004	0.000943	**874**	0.008846	0.002801
**239**	0.023789	0.024258	**211**	0.002004	0.000921	**875**	0.008846	0.002801
**240**	0.023789	0.024258	**212**	0.002004	0.000921	**876**	0.008846	0.002801
**241**	0.023789	0.024258	**213**	0.002004	0.000921	**877**	0.008846	0.002801
**242**	0.023789	0.024258	**214**	0.002004	0.000921	**878**	0.008846	0.002801
**243**	0.023789	0.024258	**215**	0.002004	0.000921	**879**	0.008846	0.002801
**244**	0.023789	0.024258	**216**	0.002004	0.000921	**880**	0.008846	0.002801
**245**	0.023789	0.024258	**217**	0.002004	0.000921	**881**	0.008846	0.002801
**246**	0.023789	0.024258	**218**	0.002004	0.000915	**882**	0.008846	0.002801
**247**	0.023789	0.024258	**219**	0.002004	0.000915	**883**	0.008846	0.002801
**248**	0.023789	0.024258	**220**	0.002004	0.000915	**884**	0.008846	0.002801
**249**	0.023789	0.024258	**221**	0.002004	0.000845	**885**	0.008846	0.002801
**250**	0.023789	0.024258	**222**	0.002004	0.000845	**886**	0.008846	0.002801
**251**	0.023789	0.024258	**223**	0.002004	0.000845	**887**	0.008846	0.002801
**252**	0.023789	0.024258	**224**	0.002004	0.000845	**888**	0.008846	0.002801
**253**	0.023789	0.024258	**225**	0.002004	0.00084	**889**	0.008846	0.002801
**254**	0.023789	0.024258	**226**	0.002004	0.00084	**890**	0.008846	0.002801
**255**	0.023789	0.024258	**227**	0.002004	0.00084	**891**	0.008846	0.002801
**256**	0.023789	0.024258	**228**	0.002004	0.00084	**892**	0.008846	0.002801
**257**	0.023789	0.024258	**229**	0.002004	0.00084	**893**	0.008846	0.002801
**258**	0.023789	0.024258	**230**	0.002004	0.00084	**894**	0.008846	0.002801
**259**	0.023789	0.024258	**231**	0.002004	0.00084	**895**	0.008846	0.002801
**260**	0.023789	0.024258	**232**	0.002004	0.000819	**896**	0.008846	0.002801
**261**	0.023789	0.024258	**233**	0.002004	0.000753	**897**	0.008846	0.002801
**262**	0.023789	0.024258	**234**	0.002004	0.000753	**898**	0.008846	0.002801
**263**	0.023789	0.024258	**235**	0.002004	0.000753	**899**	0.008846	0.002801
**264**	0.023789	0.024258	**236**	0.002004	0.000753	**900**	0.008846	0.002801
**265**	0.023789	0.024258	**237**	0.002004	0.000753	**901**	0.008846	0.002801
**266**	0.023789	0.024258	**238**	0.002004	0.000753	**902**	0.008846	0.002801
**267**	0.023789	0.024258	**239**	0.002004	0.000753	**903**	0.008846	0.002801
**268**	0.023789	0.024258	**240**	0.002004	0.000753	**904**	0.008846	0.002801
**269**	0.023789	0.024258	**241**	0.002004	0.000737	**905**	0.008846	0.002801
**270**	0.023789	0.024258	**242**	0.002004	0.000737	**906**	0.008846	0.002801
**271**	0.023789	0.024258	**243**	0.002004	0.000731	**907**	0.008846	0.002801
**272**	0.023789	0.024258	**244**	0.002004	0.000731	**908**	0.008846	0.002801

**273**	0.023789	0.024258	**245**	0.001938	0.000731	**909**	0.008846	0.002801
**274**	0.023789	0.024258	**246**	0.001938	0.000731	**910**	0.008846	0.002801
**275**	0.023789	0.024258	**247**	0.001938	0.000731	**911**	0.008846	0.002801
**276**	0.023789	0.024258	**248**	0.001938	0.000731	**912**	0.008846	0.002801
**277**	0.023789	0.024258	**249**	0.001938	0.000731	**913**	0.008846	0.002801
**278**	0.023789	0.024258	**250**	0.001938	0.000731	**914**	0.008846	0.002801
**279**	0.023789	0.024258	**251**	0.001938	0.00072	**915**	0.008846	0.002801
**280**	0.023789	0.024258	**252**	0.001938	0.00072	**916**	0.008846	0.002801
**281**	0.023789	0.024258	**253**	0.001938	0.00072	**917**	0.008846	0.002801
**282**	0.023789	0.024258	**254**	0.001871	0.00072	**918**	0.008846	0.002801
**283**	0.023789	0.024258	**255**	0.001871	0.00072	**919**	0.008846	0.002801
**284**	0.023789	0.024258	**256**	0.001871	0.00072	**920**	0.008846	0.002801
**285**	0.023789	0.024258	**257**	0.001871	0.00072	**921**	0.008846	0.002801
**286**	0.023789	0.024258	**258**	0.001871	0.00072	**922**	0.008846	0.002801
**287**	0.023789	0.024258	**259**	0.001871	0.00072	**923**	0.008846	0.002801
**288**	0.023789	0.024258	**260**	0.001871	0.000717	**924**	0.008846	0.002801
**289**	0.023789	0.024258	**261**	0.001871	0.000717	**925**	0.008846	0.002801
**290**	0.023789	0.024258	**262**	0.001871	0.000717	**926**	0.008846	0.002801
**291**	0.023789	0.024258	**263**	0.001871	0.000717	**927**	0.008846	0.002801
**292**	0.023789	0.024258	**264**	0.00184	0.000706	**928**	0.008846	0.002801
**293**	0.023789	0.024258	**265**	0.00184	0.000706	**929**	0.008846	0.002801
**294**	0.023789	0.024258	**266**	0.00184	0.000701	**930**	0.008846	0.002801
**295**	0.023789	0.024258	**267**	0.001804	0.000674	**931**	0.008846	0.002801
**296**	0.023789	0.024258	**268**	0.001804	0.000663	**932**	0.008846	0.002801
**297**	0.023789	0.024258	**269**	0.001804	0.000663	**933**	0.008846	0.002801
**298**	0.023789	0.024258	**270**	0.001804	0.000659	**934**	0.008846	0.002801
**299**	0.023789	0.024258	**271**	0.001804	0.000657	**935**	0.008846	0.002801
**300**	0.023789	0.024258	**272**	0.001804	0.000657	**936**	0.008846	0.002801
**301**	0.023789	0.024258	**273**	0.001804	0.000657	**937**	0.008846	0.002801
**302**	0.023789	0.024258	**274**	0.001804	0.000637	**938**	0.008846	0.002801
**303**	0.023789	0.024258	**275**	0.0018	0.000637	**939**	0.008846	0.002801
**304**	0.023789	0.024258	**276**	0.0018	0.000637	**940**	0.008846	0.002801
**305**	0.023789	0.024258	**277**	0.0018	0.000627	**941**	0.008846	0.002801
**306**	0.023789	0.024258	**278**	0.0018	0.000617	**942**	0.008846	0.002801
**307**	0.023789	0.024258	**279**	0.0018	0.000617	**943**	0.008846	0.002801
**308**	0.023789	0.024258	**280**	0.001789	0.000617	**944**	0.008846	0.002801
**309**	0.023789	0.024258	**281**	0.001789	0.000613	**945**	0.008846	0.002801
**310**	0.023789	0.024258	**282**	0.001789	0.000609	**946**	0.008846	0.002801
**311**	0.023789	0.024258	**283**	0.001771	0.000609	**947**	0.008846	0.002801
**312**	0.023789	0.024258	**284**	0.001771	0.000609	**948**	0.008846	0.002801
**313**	0.023789	0.024258	**285**	0.001771	0.000608	**949**	0.008846	0.002801
**314**	0.023789	0.024258	**286**	0.001771	0.000607	**950**	0.008846	0.002801
**315**	0.023789	0.024258	**287**	0.001771	0.000597			
**316**	0.023789	0.024258	**288**	0.001771	0.000593			
**317**	0.023789	0.024258	**289**	0.001738	0.000593			
**318**	0.023789	0.024258	**290**	0.001738	0.000585			

Table 21. Damage identification results of shear frame in the first damage scenario using the information of the first mode

Story	Actual	1 Mode 0% noise	1 Mode 3% noise	1 Mode 8% noise
**1**	0	0.2	0.04	0.34
**2**	10	9.98	10.4	10.3
**3**	0	0.1	0.47	0.48
**4**	0	0	0.45	0
**5**	0	0.1	0.63	0.74
**6**	0	0.1	0	0.12
**7**	0	0	0.67	0.96
**8**	0	0.2	0.98	0
**9**	0	0	0	0
**10**	0	0.1	0	0
**11**	0	0	0	1.1
**12**	0	0	0	0
**13**	0	0	1.5	0
**14**	0	0.1	0.43	1.26
**15**	0	0.2	1.86	0.13

Table 22. Damage identification results of shear frame in the second damage scenario using the information of the first mode

Story	Actual	1 Mode 0% noise	1 Mode 3% noise	1 Mode 8% noise
**1**	0	0	0	0.1
**2**	0	0	0.1	0
**3**	0	0	0	0.3
**4**	5	4.91	5	5.7
**5**	0	0	0.1	0.1
**6**	0	0	0	0.1
**7**	0	0	0	0.1
**8**	15	14.94	15.1	15
**9**	0	0	0	0.2
**10**	0	0	0	0
**10**	0	0	0	0
**12**	0	0	0	0.2
**13**	0	0	0	0.1
**14**	0	0	0	0.1
**15**	0	0.1	0.1	0

Table 23. Damage identification results of shear frame in the third damage scenario using the information of the first mode

Story	Actual	1 Mode 0% noise	1 Mode 3% noise	1 Mode 8% noise
**1**	0	0	0	0.1
**2**	0	0	0.1	0
**3**	0	0	0	0.3
**4**	5	4.91	5	5.7
**5**	0	0	0.1	0.1
**6**	0	0	0	0.1
**7**	0	0	0	0.1
**8**	15	14.94	15.1	15
**9**	0	0	0	0.2
**10**	0	0	0	0
**10**	0	0	0	0
**12**	0	0	0	0.2
**13**	0	0	0	0.1
**14**	0	0	0	0.1
**15**	0	0.1	0.1	0

Table 24. Damage identification results of shear frame in the first damage scenario using the information of the first 3 modes

Story	Actual	3 Modes 0% Noise	3 Modes 3% Noise	3 Modes 8% Noise
**1**	0	0.1	0.2	0.12
**2**	10	9.97	9.28	11.14
**3**	0	0	0	1.24
**4**	0	0.1	0	1.55
**5**	0	0	0	0.85
**6**	0	0	0.75	0.81
**7**	0	0	0	1.7
**8**	0	0	0.76	0.29
**9**	0	0	0	2.25
**10**	0	0	0	0.15
**11**	0	0	0	2.57
**12**	0	0	0.37	1.36
**13**	0	0	0	3.79
**14**	0	0	0	1.34
**15**	0	0	0.13	0

Table 25. Damage identification results of shear frame in the second damage scenario using the information of the first 3 modes

Story	Actual	3 Modes 0% Noise	3 Modes 3% Noise	3 Modes 8% Noise
**1**	0	0	0.35	0
**2**	0	0	0.35	0
**3**	0	0	0	0
**4**	5	4.77	5.5	6
**5**	0	0.1	0.61	0.21
**6**	0	0	0	0
**7**	0	0	0	0
**8**	15	14.99	15.89	15.53
**9**	0	0	0	0
**10**	0	0	0.59	0.64
**10**	0	0	0.22	0
**12**	0	0	0.67	0.94
**13**	0	0	0.57	0.41
**14**	0	0	0	0
**15**	0	0	0.75	0.78

Table 26. Damage identification results of shear frame in the third damage scenario using the information of the first 3 modes

Story	Actual	3 Modes 0% Noise	3 Modes 3% Noise	3 Modes 8% Noise
**1**	10	9.85	9.33	8.93
**2**	0	0	0	0.43
**3**	0	0.37	0.1	0.22
**4**	0	0.77	0.7	3.76
**5**	25	25.07	25.08	23.96
**6**	0	0	0.19	1
**7**	0	0.46	0	0.82
**8**	0	0	1.23	0
**9**	15	15.32	12.62	16.95
**10**	0	0.39	0	2.89
**10**	0	0.23	0	0
**12**	0	0.24	0.47	1.67
**13**	20	20.57	18.75	22.05
**14**	0	0	0.55	0.43
**15**	0	0	0.92	0.96

Table 27. Convergence of the first damage scenario in the absence of noise for first 1 and 3 modes

Iterations	1 Mode	3 Modes	Iterations	1 Modes	3 Modes	Iterations	1 Modes	3 Modes
**1**	0.26395	0.22125	**137**	0.003579	0.001751	**273**	0.001804	0.000657
**2**	0.18351	0.14521	**138**	0.003579	0.001751	**274**	0.001804	0.000637
**3**	0.18351	0.12934	**139**	0.003579	0.001477	**275**	0.0018	0.000637
**4**	0.17012	0.077742	**140**	0.003579	0.001477	**276**	0.0018	0.000637
**5**	0.12417	0.077742	**141**	0.003579	0.001477	**277**	0.0018	0.000627
**6**	0.1171	0.070377	**142**	0.003579	0.001477	**278**	0.0018	0.000617
**7**	0.093362	0.066534	**143**	0.003579	0.001477	**279**	0.0018	0.000617
**8**	0.093362	0.059616	**144**	0.003579	0.001477	**280**	0.001789	0.000617
**9**	0.093362	0.054243	**145**	0.003579	0.001477	**281**	0.001789	0.000613
**10**	0.068475	0.05064	**146**	0.003579	0.001477	**282**	0.001789	0.000609
**11**	0.068475	0.046154	**147**	0.003441	0.001477	**283**	0.001771	0.000609
**12**	0.059404	0.042276	**148**	0.003441	0.001477	**284**	0.001771	0.000609
**13**	0.059404	0.028876	**149**	0.003441	0.001477	**285**	0.001771	0.000608
**14**	0.057301	0.028876	**150**	0.003441	0.001477	**286**	0.001771	0.000607
**15**	0.032047	0.028071	**151**	0.002817	0.001477	**287**	0.001771	0.000597
**16**	0.032047	0.02637	**152**	0.002817	0.001477	**288**	0.001771	0.000593
**17**	0.032047	0.018802	**153**	0.002817	0.001477	**289**	0.001738	0.000593
**18**	0.032047	0.018802	**154**	0.002817	0.001477	**290**	0.001738	0.000585
**19**	0.030671	0.018802	**155**	0.002817	0.001477	**291**	0.001738	0.000583
**20**	0.024392	0.018224	**156**	0.002817	0.001477	**292**	0.001738	0.000583
**21**	0.024392	0.016296	**157**	0.002817	0.001477	**293**	0.001738	0.000579
**22**	0.024392	0.016296	**158**	0.002817	0.001441	**294**	0.001732	0.000577
**23**	0.024392	0.009464	**159**	0.002817	0.001441	**295**	0.001729	0.000577
**24**	0.024392	0.009464	**160**	0.002817	0.001368	**296**	0.001729	0.000576
**25**	0.024392	0.009464	**161**	0.002817	0.001368	**297**	0.001729	0.000576
**26**	0.020544	0.007757	**162**	0.002817	0.00133	**298**	0.001729	0.000575
**27**	0.020544	0.007626	**163**	0.002817	0.00133	**299**	0.001729	0.000574
**28**	0.020544	0.005778	**164**	0.002817	0.00133	**300**	0.001725	0.000574
**29**	0.020544	0.005279	**165**	0.002817	0.00133			
**30**	0.020544	0.005279	**166**	0.002817	0.00133			
**31**	0.020544	0.005279	**167**	0.002817	0.00133			
**32**	0.020544	0.005279	**168**	0.002817	0.00133			
**33**	0.020544	0.005279	**169**	0.002817	0.00133			
**34**	0.020544	0.004987	**170**	0.002817	0.00133			
**35**	0.019134	0.004987	**171**	0.002817	0.001322			
**36**	0.019134	0.003377	**172**	0.002817	0.001322			
**37**	0.016242	0.003377	**173**	0.002322	0.001318			
**38**	0.016242	0.003377	**174**	0.002322	0.001318			
**39**	0.016242	0.003377	**175**	0.002322	0.001318			
**40**	0.016242	0.003377	**176**	0.002322	0.001318			
**41**	0.016242	0.003377	**177**	0.002322	0.001318			
**42**	0.016242	0.003377	**178**	0.002322	0.001318			
**43**	0.016242	0.003377	**179**	0.002322	0.001148			
**44**	0.012603	0.003154	**180**	0.002322	0.001148			

**45**	0.012603	0.003154	**181**	0.002322	0.001148			
**46**	0.012603	0.003154	**182**	0.002322	0.001148			
**47**	0.012171	0.003154	**183**	0.002322	0.001148			
**48**	0.012171	0.003154	**184**	0.002322	0.001148			
**49**	0.012171	0.003154	**185**	0.002322	0.001146			
**50**	0.012171	0.003154	**186**	0.002322	0.001146			
**51**	0.012171	0.003154	**187**	0.002322	0.001146			
**52**	0.012171	0.003154	**188**	0.002322	0.001146			
**53**	0.012171	0.003154	**189**	0.002322	0.001146			
**54**	0.012171	0.003154	**190**	0.002322	0.001146			
**55**	0.012171	0.003154	**191**	0.002322	0.001146			
**56**	0.012171	0.003154	**192**	0.002322	0.001146			
**57**	0.012171	0.00294	**193**	0.002322	0.000987			
**58**	0.012171	0.00294	**194**	0.002322	0.000987			
**59**	0.012171	0.00294	**195**	0.002322	0.000987			
**60**	0.012171	0.00294	**196**	0.002322	0.000987			
**61**	0.012171	0.00294	**197**	0.002149	0.000987			
**62**	0.012171	0.00294	**198**	0.002149	0.000987			
**63**	0.008727	0.00294	**199**	0.002149	0.000987			
**64**	0.008727	0.00294	**200**	0.002149	0.000987			
**65**	0.008727	0.002852	**201**	0.002149	0.000987			
**66**	0.008727	0.002852	**202**	0.002149	0.000987			
**67**	0.008446	0.002852	**203**	0.002149	0.000987			
**68**	0.008446	0.002852	**204**	0.002149	0.000983			
**69**	0.008446	0.002852	**205**	0.002149	0.000983			
**70**	0.008446	0.002852	**206**	0.002149	0.000983			
**71**	0.008446	0.002852	**207**	0.002149	0.000983			
**72**	0.008446	0.002554	**208**	0.002004	0.000964			
**73**	0.008446	0.002554	**209**	0.002004	0.000943			
**74**	0.008446	0.002554	**210**	0.002004	0.000943			
**75**	0.008446	0.002554	**211**	0.002004	0.000921			
**76**	0.008446	0.002554	**212**	0.002004	0.000921			
**77**	0.006365	0.002554	**213**	0.002004	0.000921			
**78**	0.006365	0.002554	**214**	0.002004	0.000921			
**79**	0.006365	0.002554	**215**	0.002004	0.000921			
**80**	0.006113	0.002554	**216**	0.002004	0.000921			
**81**	0.006113	0.002554	**217**	0.002004	0.000921			
**82**	0.006113	0.002554	**218**	0.002004	0.000915			
**83**	0.006113	0.002399	**219**	0.002004	0.000915			
**84**	0.006113	0.002399	**220**	0.002004	0.000915			
**85**	0.006113	0.002399	**221**	0.002004	0.000845			
**86**	0.006113	0.002399	**222**	0.002004	0.000845			
**87**	0.006113	0.002399	**223**	0.002004	0.000845			
**88**	0.006113	0.002399	**224**	0.002004	0.000845			
**89**	0.005796	0.002265	**225**	0.002004	0.00084			
**90**	0.005796	0.002265	**226**	0.002004	0.00084			

**91**	0.005796	0.002265	**227**	0.002004	0.00084			
**92**	0.005796	0.002265	**228**	0.002004	0.00084			
**93**	0.005796	0.002265	**229**	0.002004	0.00084			
**94**	0.005796	0.002265	**230**	0.002004	0.00084			
**95**	0.005796	0.002265	**231**	0.002004	0.00084			
**96**	0.005796	0.002265	**232**	0.002004	0.000819			
**97**	0.005796	0.002265	**233**	0.002004	0.000753			
**98**	0.004818	0.002265	**234**	0.002004	0.000753			
**99**	0.004818	0.002265	**235**	0.002004	0.000753			
**100**	0.004818	0.002265	**236**	0.002004	0.000753			
**101**	0.004818	0.002265	**237**	0.002004	0.000753			
**102**	0.004818	0.002265	**238**	0.002004	0.000753			
**103**	0.004818	0.002265	**239**	0.002004	0.000753			
**104**	0.004818	0.002265	**240**	0.002004	0.000753			
**105**	0.004818	0.002265	**241**	0.002004	0.000737			
**106**	0.004818	0.002265	**242**	0.002004	0.000737			
**107**	0.004818	0.002265	**243**	0.002004	0.000731			
**108**	0.003913	0.002265	**244**	0.002004	0.000731			
**109**	0.003913	0.002265	**245**	0.001938	0.000731			
**110**	0.003913	0.002265	**246**	0.001938	0.000731			
**111**	0.003913	0.002265	**247**	0.001938	0.000731			
**112**	0.003913	0.002265	**248**	0.001938	0.000731			
**113**	0.003913	0.002265	**249**	0.001938	0.000731			
**114**	0.003913	0.001795	**250**	0.001938	0.000731			
**115**	0.003913	0.001795	**251**	0.001938	0.00072			
**116**	0.003913	0.001795	**252**	0.001938	0.00072			
**117**	0.003913	0.001795	**253**	0.001938	0.00072			
**118**	0.003913	0.001795	**254**	0.001871	0.00072			
**119**	0.003913	0.001795	**255**	0.001871	0.00072			
**120**	0.003704	0.001795	**256**	0.001871	0.00072			
**121**	0.003704	0.001795	**257**	0.001871	0.00072			
**122**	0.003704	0.001795	**258**	0.001871	0.00072			
**123**	0.003704	0.001795	**259**	0.001871	0.00072			
**124**	0.003611	0.001795	**260**	0.001871	0.000717			
**125**	0.003611	0.001795	**261**	0.001871	0.000717			
**126**	0.003611	0.001795	**262**	0.001871	0.000717			
**127**	0.003611	0.001795	**263**	0.001871	0.000717			
**128**	0.003611	0.001795	**264**	0.00184	0.000706			
**129**	0.003611	0.001795	**265**	0.00184	0.000706			
**130**	0.003611	0.001751	**266**	0.00184	0.000701			
**131**	0.003611	0.001751	**267**	0.001804	0.000674			
**132**	0.003579	0.001751	**268**	0.001804	0.000663			
**133**	0.003579	0.001751	**269**	0.001804	0.000663			
**134**	0.003579	0.001751	**270**	0.001804	0.000659			
**135**	0.003579	0.001751	**271**	0.001804	0.000657			
**136**	0.003579	0.001751	**272**	0.001804	0.000657			

Table 28. Convergence of the first damage scenario in the presence of 8% noise for first 1 and 3 modes

Iterations	1 Mode	3 Modes	Iterations	1 Modes	3 Modes	Iterations	1 Modes	3 Modes
**1**	0.13583	0.17544	**137**	0.000564	0.001441	**273**	0.000495	0.000841
**2**	0.074456	0.10179	**138**	0.000564	0.001441	**274**	0.000495	0.000841
**3**	0.03082	0.066028	**139**	0.000564	0.001441	**275**	0.000492	0.000841
**4**	0.019041	0.026899	**140**	0.000564	0.001441	**276**	0.000492	0.000841
**5**	0.008904	0.017097	**141**	0.000564	0.001441	**277**	0.000492	0.000833
**6**	0.006504	0.008094	**142**	0.000542	0.001441	**278**	0.000492	0.000833
**7**	0.003476	0.006119	**143**	0.000542	0.001441	**279**	0.000491	0.000833
**8**	0.003476	0.006091	**144**	0.000542	0.001441	**280**	0.000491	0.000833
**9**	0.002878	0.005116	**145**	0.000542	0.001441	**281**	0.000491	0.000833
**10**	0.002341	0.002712	**146**	0.000542	0.001441	**282**	0.000491	0.000833
**11**	0.001702	0.002712	**147**	0.000542	0.001441	**283**	0.000491	0.000814
**12**	0.001702	0.002712	**148**	0.000542	0.001441	**284**	0.000491	0.000814
**13**	0.001325	0.002712	**149**	0.000542	0.001441	**285**	0.000479	0.000814
**14**	0.001325	0.002712	**150**	0.000542	0.001441	**286**	0.000479	0.000804
**15**	0.001325	0.002558	**151**	0.000542	0.001441	**287**	0.000479	0.0008
**16**	0.001325	0.002558	**152**	0.000542	0.001441	**288**	0.000479	0.000785
**17**	0.001325	0.002558	**153**	0.000542	0.001441	**289**	0.000479	0.000765
**18**	0.001325	0.002558	**154**	0.000542	0.001441	**290**	0.000479	0.000765
**19**	0.00124	0.002375	**155**	0.000542	0.001441	**291**	0.000479	0.000765
**20**	0.00124	0.002375	**156**	0.000542	0.001441	**292**	0.000479	0.000765
**21**	0.00124	0.002375	**157**	0.000542	0.001441	**293**	0.000479	0.000765
**22**	0.00124	0.002375	**158**	0.000542	0.001441	**294**	0.000479	0.000765
**23**	0.00124	0.002375	**159**	0.000542	0.001378	**295**	0.000479	0.000759
**24**	0.00124	0.002375	**160**	0.000542	0.001378	**296**	0.000479	0.000759
**25**	0.001232	0.002375	**161**	0.000542	0.001378	**297**	0.000479	0.000759
**26**	0.001232	0.002375	**162**	0.000542	0.001378	**298**	0.000477	0.000749
**27**	0.001232	0.002232	**163**	0.000542	0.001378	**299**	0.000477	0.000749
**28**	0.001191	0.002232	**164**	0.000542	0.001378	**300**	0.000474	0.000749
**29**	0.001191	0.002232	**165**	0.000542	0.001378			
**30**	0.001191	0.002232	**166**	0.000542	0.001378			
**31**	0.001191	0.002232	**167**	0.000542	0.001378			
**32**	0.001191	0.002232	**168**	0.000542	0.001378			
**33**	0.001191	0.002232	**169**	0.000542	0.001378			
**34**	0.001191	0.002232	**170**	0.000542	0.001378			
**35**	0.000956	0.002232	**171**	0.000542	0.001378			
**36**	0.000956	0.002232	**172**	0.000542	0.001378			
**37**	0.000944	0.002232	**173**	0.000542	0.001378			
**38**	0.000944	0.002232	**174**	0.000542	0.001378			
**39**	0.000813	0.002231	**175**	0.000542	0.001378			
**40**	0.000813	0.002231	**176**	0.000542	0.001378			
**41**	0.000813	0.002231	**177**	0.000542	0.001378			
**42**	0.000813	0.002231	**178**	0.000542	0.001378			
**43**	0.000813	0.002146	**179**	0.000542	0.001361			
**44**	0.000813	0.002146	**180**	0.000542	0.001361			

**45**	0.000813	0.002146	**181**	0.000542	0.001361			
**46**	0.000813	0.002146	**182**	0.000542	0.001361			
**47**	0.000813	0.002146	**183**	0.000542	0.001361			
**48**	0.000813	0.002146	**184**	0.000542	0.001361			
**49**	0.000813	0.002146	**185**	0.000542	0.001361			
**50**	0.000813	0.002146	**186**	0.000542	0.001361			
**51**	0.000813	0.002146	**187**	0.000542	0.001361			
**52**	0.000813	0.002146	**188**	0.000542	0.001361			
**53**	0.000813	0.002146	**189**	0.000542	0.001326			
**54**	0.000813	0.002146	**190**	0.000542	0.001326			
**55**	0.000813	0.002146	**191**	0.000542	0.001326			
**56**	0.000813	0.002146	**192**	0.000542	0.001213			
**57**	0.000813	0.002146	**193**	0.000542	0.001213			
**58**	0.000813	0.002146	**194**	0.000542	0.001213			
**59**	0.000813	0.001975	**195**	0.000532	0.001213			
**60**	0.000813	0.001975	**196**	0.000532	0.001213			
**61**	0.000813	0.001975	**197**	0.000532	0.001178			
**62**	0.000813	0.001975	**198**	0.000532	0.001178			
**63**	0.000813	0.001799	**199**	0.000514	0.001086			
**64**	0.000813	0.001799	**200**	0.000514	0.001086			
**65**	0.000813	0.001799	**201**	0.000514	0.001086			
**66**	0.000813	0.001799	**202**	0.000514	0.001086			
**67**	0.000813	0.001799	**203**	0.000514	0.001086			
**68**	0.000813	0.001799	**204**	0.000514	0.001086			
**69**	0.000813	0.001799	**205**	0.000514	0.001086			
**70**	0.000813	0.001799	**206**	0.000514	0.001086			
**71**	0.000744	0.001799	**207**	0.000514	0.001086			
**72**	0.000744	0.001799	**208**	0.000514	0.001086			
**73**	0.000744	0.001799	**209**	0.000514	0.001086			
**74**	0.000744	0.001799	**210**	0.000514	0.001086			
**75**	0.000744	0.001799	**211**	0.000514	0.001086			
**76**	0.000744	0.001799	**212**	0.000514	0.001086			
**77**	0.000744	0.001799	**213**	0.000514	0.001086			
**78**	0.000744	0.001799	**214**	0.000514	0.001086			
**79**	0.000744	0.001799	**215**	0.000514	0.001072			
**80**	0.000744	0.001799	**216**	0.000514	0.001072			
**81**	0.000744	0.001799	**217**	0.000514	0.001072			
**82**	0.000744	0.001722	**218**	0.000514	0.001072			
**83**	0.000744	0.001722	**219**	0.000514	0.001072			
**84**	0.000744	0.001722	**220**	0.000514	0.001072			
**85**	0.000744	0.001722	**221**	0.000514	0.001072			
**86**	0.000744	0.001722	**222**	0.000514	0.001072			
**87**	0.000645	0.001722	**223**	0.000514	0.001072			
**88**	0.000645	0.001722	**224**	0.000514	0.001072			
**89**	0.000645	0.001722	**225**	0.000514	0.001072			
**90**	0.000645	0.001722	**226**	0.000514	0.001072			

**91**	0.000645	0.001722	**227**	0.000514	0.001072			
**92**	0.000645	0.001722	**228**	0.000514	0.001072			
**93**	0.000645	0.001722	**229**	0.000514	0.001041			
**94**	0.000645	0.001722	**230**	0.000514	0.001041			
**95**	0.000645	0.001722	**231**	0.000514	0.001041			
**96**	0.000645	0.001722	**232**	0.000514	0.001041			
**97**	0.000645	0.001722	**233**	0.000514	0.001041			
**98**	0.000645	0.001722	**234**	0.000514	0.001041			
**99**	0.000645	0.001722	**235**	0.000514	0.001041			
**100**	0.000645	0.001704	**236**	0.000514	0.001041			
**101**	0.000645	0.001704	**237**	0.000514	0.001041			
**102**	0.000645	0.001704	**238**	0.000514	0.001041			
**103**	0.000645	0.001704	**239**	0.000514	0.001001			
**104**	0.000645	0.001441	**240**	0.000514	0.001001			
**105**	0.000645	0.001441	**241**	0.000514	0.000996			
**106**	0.000645	0.001441	**242**	0.000514	0.000996			
**107**	0.000645	0.001441	**243**	0.000514	0.000996			
**108**	0.000645	0.001441	**244**	0.000514	0.000996			
**109**	0.000645	0.001441	**245**	0.000514	0.000996			
**110**	0.000645	0.001441	**246**	0.000514	0.000996			
**111**	0.000645	0.001441	**247**	0.000514	0.000969			
**112**	0.000645	0.001441	**248**	0.000514	0.000969			
**113**	0.000645	0.001441	**249**	0.000514	0.000969			
**114**	0.000645	0.001441	**250**	0.000514	0.000969			
**115**	0.000645	0.001441	**251**	0.000514	0.000969			
**116**	0.000645	0.001441	**252**	0.000514	0.000969			
**117**	0.000645	0.001441	**253**	0.000514	0.000969			
**118**	0.00064	0.001441	**254**	0.000514	0.000969			
**119**	0.00064	0.001441	**255**	0.000499	0.000969			
**120**	0.000587	0.001441	**256**	0.000499	0.000969			
**121**	0.000587	0.001441	**257**	0.000499	0.000969			
**122**	0.000587	0.001441	**258**	0.000499	0.000969			
**123**	0.000587	0.001441	**259**	0.000499	0.000969			
**124**	0.000587	0.001441	**260**	0.000499	0.000939			
**125**	0.000587	0.001441	**261**	0.000499	0.000939			
**126**	0.000587	0.001441	**262**	0.000499	0.000939			
**127**	0.000587	0.001441	**263**	0.000499	0.0009			
**128**	0.000587	0.001441	**264**	0.000499	0.000841			
**129**	0.000587	0.001441	**265**	0.000499	0.000841			
**130**	0.000587	0.001441	**266**	0.000499	0.000841			
**131**	0.000587	0.001441	**267**	0.000499	0.000841			
**132**	0.000587	0.001441	**268**	0.000499	0.000841			
**133**	0.000587	0.001441	**269**	0.000495	0.000841			
**134**	0.000587	0.001441	**270**	0.000495	0.000841			
**135**	0.000564	0.001441	**271**	0.000495	0.000841			
**136**	0.000564	0.001441	**272**	0.000495	0.000841			

Table 29. Convergence of the third damage scenario in the absence of noise for first 1 and 3 modes

Iterations	1 Mode	3 Modes	Iterations	1 Modes	3 Modes	Iterations	1 Modes	3 Modes
**1**	0.27104	0.23909	**137**	0.016286	0.009089	**273**	0.010185	0.00645
**2**	0.22304	0.19288	**138**	0.016286	0.009089	**274**	0.010185	0.00645
**3**	0.22304	0.19288	**139**	0.016286	0.009089	**275**	0.010185	0.006418
**4**	0.21699	0.16541	**140**	0.016286	0.009089	**276**	0.010185	0.006418
**5**	0.16703	0.14942	**141**	0.016286	0.009089	**277**	0.010185	0.006418
**6**	0.12973	0.13156	**142**	0.016286	0.009089	**278**	0.010185	0.006418
**7**	0.11606	0.11642	**143**	0.014893	0.009089	**279**	0.010185	0.006418
**8**	0.10552	0.11642	**144**	0.014893	0.009089	**280**	0.00977	0.006299
**9**	0.099874	0.11642	**145**	0.014893	0.009089	**281**	0.00977	0.006299
**10**	0.075116	0.10461	**146**	0.014893	0.009089	**282**	0.00977	0.006299
**11**	0.075116	0.07299	**147**	0.014893	0.009089	**283**	0.00977	0.006299
**12**	0.075116	0.07299	**148**	0.014893	0.009089	**284**	0.00977	0.006299
**13**	0.063191	0.069046	**149**	0.014893	0.009089	**285**	0.00977	0.006299
**14**	0.063191	0.061076	**150**	0.014893	0.009089	**286**	0.00977	0.006299
**15**	0.060659	0.055739	**151**	0.014893	0.009089	**287**	0.00977	0.006299
**16**	0.060659	0.048747	**152**	0.014893	0.009089	**288**	0.00977	0.006121
**17**	0.060659	0.04085	**153**	0.014893	0.009089	**289**	0.00977	0.006037
**18**	0.055299	0.04085	**154**	0.014893	0.009089	**290**	0.00977	0.005645
**19**	0.055299	0.04085	**155**	0.014893	0.009089	**291**	0.00977	0.005645
**20**	0.055299	0.04085	**156**	0.014893	0.009089	**292**	0.009692	0.005645
**21**	0.055299	0.033073	**157**	0.014893	0.009089	**293**	0.009662	0.005645
**22**	0.055299	0.033073	**158**	0.014893	0.009089	**294**	0.009605	0.005645
**23**	0.055299	0.033073	**159**	0.014893	0.009089	**295**	0.009605	0.005645
**24**	0.055299	0.033073	**160**	0.014561	0.009089	**296**	0.009605	0.005645
**25**	0.048062	0.029637	**161**	0.014561	0.009089	**297**	0.009599	0.005645
**26**	0.048062	0.029637	**162**	0.014561	0.009089	**298**	0.009597	0.005645
**27**	0.048062	0.029637	**163**	0.014491	0.009089	**299**	0.009586	0.005605
**28**	0.048062	0.029637	**164**	0.011748	0.009089	**300**	0.00958	0.00559
**29**	0.048062	0.029637	**165**	0.011748	0.009089			
**30**	0.048062	0.029637	**166**	0.011748	0.009089			
**31**	0.045049	0.029637	**167**	0.011748	0.009089			
**32**	0.045049	0.029637	**168**	0.011748	0.009089			
**33**	0.045049	0.029637	**169**	0.011748	0.009089			
**34**	0.045049	0.029637	**170**	0.011748	0.009089			
**35**	0.045049	0.029637	**171**	0.011748	0.009089			
**36**	0.045049	0.029637	**172**	0.011748	0.009089			
**37**	0.045049	0.029637	**173**	0.011748	0.009089			
**38**	0.042266	0.029637	**174**	0.011748	0.009089			
**39**	0.042266	0.029637	**175**	0.011748	0.009089			
**40**	0.042266	0.0261	**176**	0.011748	0.009089			
**41**	0.042266	0.0261	**177**	0.011748	0.009089			
**42**	0.042266	0.0261	**178**	0.011748	0.009089			
**43**	0.042266	0.0261	**179**	0.011748	0.009089			
**44**	0.042266	0.0261	**180**	0.011748	0.009089			

**45**	0.042266	0.0261	**181**	0.011748	0.009089			
**46**	0.042266	0.0261	**182**	0.011748	0.009089			
**47**	0.042266	0.0261	**183**	0.011748	0.009089			
**48**	0.042266	0.0261	**184**	0.011748	0.007072			
**49**	0.042266	0.0261	**185**	0.011748	0.007072			
**50**	0.042266	0.0261	**186**	0.011748	0.007072			
**51**	0.042266	0.0261	**187**	0.011748	0.007072			
**52**	0.042266	0.0261	**188**	0.011748	0.007072			
**53**	0.042266	0.0261	**189**	0.011748	0.007072			
**54**	0.042266	0.0261	**190**	0.011748	0.007072			
**55**	0.042266	0.018308	**191**	0.011748	0.007072			
**56**	0.042266	0.018308	**192**	0.011748	0.007072			
**57**	0.042266	0.018308	**193**	0.011748	0.007072			
**58**	0.04215	0.018308	**194**	0.011748	0.007072			
**59**	0.042035	0.018308	**195**	0.011748	0.007072			
**60**	0.042035	0.018308	**196**	0.011748	0.007072			
**61**	0.042035	0.018308	**197**	0.011748	0.007072			
**62**	0.035384	0.018308	**198**	0.011748	0.007072			
**63**	0.035384	0.018308	**199**	0.011748	0.007072			
**64**	0.025382	0.018308	**200**	0.011748	0.007072			
**65**	0.025382	0.018308	**201**	0.011748	0.007072			
**66**	0.025382	0.018086	**202**	0.011748	0.007072			
**67**	0.025382	0.018086	**203**	0.011748	0.007072			
**68**	0.025382	0.018086	**204**	0.011748	0.007072			
**69**	0.025382	0.018086	**205**	0.011748	0.007072			
**70**	0.025382	0.018086	**206**	0.011748	0.007072			
**71**	0.025382	0.018086	**207**	0.011748	0.007072			
**72**	0.025382	0.018086	**208**	0.011748	0.007072			
**73**	0.025382	0.018086	**209**	0.011748	0.007072			
**74**	0.02418	0.018086	**210**	0.011748	0.007072			
**75**	0.02418	0.017743	**211**	0.010404	0.007072			
**76**	0.02418	0.017743	**212**	0.010404	0.007072			
**77**	0.02418	0.017743	**213**	0.010404	0.007072			
**78**	0.02418	0.017743	**214**	0.010404	0.007072			
**79**	0.02418	0.017743	**215**	0.010404	0.007072			
**80**	0.02418	0.017743	**216**	0.010404	0.007072			
**81**	0.022849	0.017743	**217**	0.010404	0.007072			
**82**	0.022849	0.017743	**218**	0.010404	0.007072			
**83**	0.022849	0.017743	**219**	0.010404	0.007072			
**84**	0.022849	0.017743	**220**	0.010404	0.007072			
**85**	0.020905	0.017743	**221**	0.010404	0.007072			
**86**	0.020905	0.017743	**222**	0.010404	0.007072			
**87**	0.020905	0.017743	**223**	0.010404	0.007072			
**88**	0.020905	0.017743	**224**	0.010404	0.007072			
**89**	0.020905	0.017743	**225**	0.010404	0.007072			
**90**	0.020905	0.017743	**226**	0.010404	0.007072			

**91**	0.020894	0.017743	**227**	0.010404	0.007072			
**92**	0.020894	0.017743	**228**	0.010404	0.007072			
**93**	0.020894	0.017743	**229**	0.010404	0.007072			
**94**	0.020894	0.017743	**230**	0.010404	0.007072			
**95**	0.019457	0.017743	**231**	0.010404	0.007072			
**96**	0.019457	0.017743	**232**	0.010404	0.007072			
**97**	0.019457	0.016798	**233**	0.010404	0.007072			
**98**	0.019457	0.016798	**234**	0.010404	0.007072			
**99**	0.019457	0.016798	**235**	0.010404	0.007072			
**100**	0.019457	0.016798	**236**	0.010404	0.007072			
**101**	0.019457	0.016798	**237**	0.010404	0.007072			
**102**	0.019457	0.016798	**238**	0.010404	0.007072			
**103**	0.019457	0.016798	**239**	0.010404	0.007072			
**104**	0.019457	0.016798	**240**	0.010404	0.007072			
**105**	0.019457	0.016798	**241**	0.010404	0.007072			
**106**	0.019457	0.016798	**242**	0.010404	0.007072			
**107**	0.019457	0.016798	**243**	0.010404	0.007072			
**108**	0.019457	0.016798	**244**	0.010404	0.007072			
**109**	0.019457	0.016798	**245**	0.010404	0.007072			
**110**	0.019457	0.016798	**246**	0.010404	0.007072			
**111**	0.019457	0.016798	**247**	0.010404	0.00645			
**112**	0.019457	0.016798	**248**	0.010404	0.00645			
**113**	0.019457	0.016798	**249**	0.010404	0.00645			
**114**	0.019457	0.016798	**250**	0.010404	0.00645			
**115**	0.019457	0.016798	**251**	0.010404	0.00645			
**116**	0.019457	0.009089	**252**	0.010404	0.00645			
**117**	0.019457	0.009089	**253**	0.010404	0.00645			
**118**	0.019457	0.009089	**254**	0.010404	0.00645			
**119**	0.019457	0.009089	**255**	0.010404	0.00645			
**120**	0.019457	0.009089	**256**	0.010404	0.00645			
**121**	0.019457	0.009089	**257**	0.010404	0.00645			
**122**	0.019457	0.009089	**258**	0.010404	0.00645			
**123**	0.019457	0.009089	**259**	0.010404	0.00645			
**124**	0.019457	0.009089	**260**	0.010404	0.00645			
**125**	0.019457	0.009089	**261**	0.010404	0.00645			
**126**	0.019457	0.009089	**262**	0.010404	0.00645			
**127**	0.016286	0.009089	**263**	0.010404	0.00645			
**128**	0.016286	0.009089	**264**	0.010404	0.00645			
**129**	0.016286	0.009089	**265**	0.010404	0.00645			
**130**	0.016286	0.009089	**266**	0.010404	0.00645			
**131**	0.016286	0.009089	**267**	0.010404	0.00645			
**132**	0.016286	0.009089	**268**	0.010404	0.00645			
**133**	0.016286	0.009089	**269**	0.010242	0.00645			
**134**	0.016286	0.009089	**270**	0.010242	0.00645			
**135**	0.016286	0.009089	**271**	0.010242	0.00645			
**136**	0.016286	0.009089	**272**	0.010185	0.00645			

Table 30. Convergence of the third damage scenario in the presence of 8% noise for first 1 and 3 modes

Iterations	1 Mode	3 Modes	Iterations	1 Modes	3 Modes	Iterations	1 Modes	3 Modes
**1**	0.30534	0.2258	**137**	0.018087	0.041637	**273**	0.009141	0.037119
**2**	0.24368	0.18575	**138**	0.018087	0.041637	**274**	0.009141	0.037119
**3**	0.16986	0.15962	**139**	0.018087	0.041637	**275**	0.009141	0.0367
**4**	0.1468	0.15962	**140**	0.018087	0.041637	**276**	0.009141	0.0367
**5**	0.11978	0.15414	**141**	0.018087	0.041637	**277**	0.009141	0.0367
**6**	0.087483	0.102	**142**	0.018087	0.041637	**278**	0.009141	0.0367
**7**	0.087483	0.102	**143**	0.01732	0.041637	**279**	0.009141	0.0367
**8**	0.072184	0.102	**144**	0.01732	0.041637	**280**	0.009141	0.0367
**9**	0.059821	0.102	**145**	0.01732	0.041637	**281**	0.009141	0.0367
**10**	0.059821	0.083557	**146**	0.01732	0.041637	**282**	0.009141	0.0367
**11**	0.058074	0.083557	**147**	0.01732	0.041637	**283**	0.009141	0.0367
**12**	0.056064	0.083557	**148**	0.01732	0.040081	**284**	0.008867	0.0367
**13**	0.056064	0.077499	**149**	0.01732	0.040081	**285**	0.008867	0.0367
**14**	0.056064	0.077499	**150**	0.01732	0.040081	**286**	0.008867	0.036668
**15**	0.056064	0.077499	**151**	0.01732	0.040081	**287**	0.008867	0.036668
**16**	0.042634	0.077499	**152**	0.01732	0.040081	**288**	0.008851	0.036668
**17**	0.038291	0.077499	**153**	0.015435	0.040081	**289**	0.008851	0.036668
**18**	0.038291	0.077499	**154**	0.015435	0.040081	**290**	0.008851	0.036643
**19**	0.038291	0.071109	**155**	0.015435	0.040081	**291**	0.008746	0.036643
**20**	0.038291	0.071109	**156**	0.015435	0.040081	**292**	0.008746	0.036552
**21**	0.038291	0.071109	**157**	0.015435	0.040081	**293**	0.008612	0.036552
**22**	0.038291	0.071109	**158**	0.015435	0.040081	**294**	0.008587	0.036552
**23**	0.038291	0.071109	**159**	0.015435	0.040081	**295**	0.008587	0.036552
**24**	0.038291	0.071109	**160**	0.015435	0.040081	**296**	0.008587	0.036552
**25**	0.038291	0.070952	**161**	0.015435	0.040081	**297**	0.008447	0.036492
**26**	0.038291	0.065307	**162**	0.015435	0.040081	**298**	0.008447	0.036467
**27**	0.034256	0.065307	**163**	0.015435	0.040081	**299**	0.008412	0.036419
**28**	0.034256	0.065307	**164**	0.013703	0.040081	**300**	0.0084	0.036399
**29**	0.022415	0.065307	**165**	0.013703	0.040081			
**30**	0.022415	0.065307	**166**	0.013703	0.040081			
**31**	0.022415	0.062094	**167**	0.013703	0.040081			
**32**	0.022415	0.062094	**168**	0.013703	0.040081			
**33**	0.022415	0.062094	**169**	0.013703	0.040081			
**34**	0.022415	0.062094	**170**	0.013703	0.040081			
**35**	0.022415	0.062094	**171**	0.013703	0.040081			
**36**	0.022415	0.062094	**172**	0.013703	0.040081			
**37**	0.022415	0.062094	**173**	0.013703	0.040081			
**38**	0.022415	0.062094	**174**	0.013703	0.040081			
**39**	0.022415	0.062094	**175**	0.013487	0.040081			
**40**	0.022415	0.062094	**176**	0.013487	0.040081			
**41**	0.022415	0.062094	**177**	0.013487	0.040081			
**42**	0.022415	0.062094	**178**	0.013487	0.040081			
**43**	0.022415	0.062094	**179**	0.013487	0.040081			
**44**	0.022415	0.062094	**180**	0.013487	0.040081			

**45**	0.022415	0.062094	**181**	0.013487	0.040081			
**46**	0.022415	0.062094	**182**	0.013487	0.040081			
**47**	0.022415	0.059694	**183**	0.013487	0.040081			
**48**	0.022415	0.059694	**184**	0.013487	0.040081			
**49**	0.022415	0.059694	**185**	0.013487	0.040081			
**50**	0.022415	0.059694	**186**	0.013487	0.040081			
**51**	0.022415	0.059694	**187**	0.013487	0.040081			
**52**	0.022415	0.059694	**188**	0.013487	0.040081			
**53**	0.022415	0.050456	**189**	0.013487	0.040081			
**54**	0.022415	0.050456	**190**	0.013487	0.040081			
**55**	0.022415	0.050456	**191**	0.013487	0.039635			
**56**	0.022415	0.050456	**192**	0.013487	0.039635			
**57**	0.022415	0.050456	**193**	0.013487	0.039635			
**58**	0.022415	0.050456	**194**	0.013487	0.039635			
**59**	0.022415	0.050456	**195**	0.013487	0.039635			
**60**	0.022415	0.050456	**196**	0.013487	0.039635			
**61**	0.022415	0.050456	**197**	0.013487	0.039635			
**62**	0.022415	0.050456	**198**	0.013487	0.039635			
**63**	0.022415	0.050456	**199**	0.013487	0.039635			
**64**	0.022415	0.050456	**200**	0.013487	0.039635			
**65**	0.022415	0.050456	**201**	0.013487	0.039635			
**66**	0.022058	0.050456	**202**	0.013099	0.039635			
**67**	0.022058	0.050456	**203**	0.013099	0.039635			
**68**	0.022058	0.050392	**204**	0.013099	0.039635			
**69**	0.022058	0.050392	**205**	0.013099	0.039635			
**70**	0.022058	0.050392	**206**	0.013099	0.039635			
**71**	0.022058	0.050392	**207**	0.013099	0.039635			
**72**	0.022058	0.050392	**208**	0.013099	0.039635			
**73**	0.022058	0.050392	**209**	0.013099	0.039635			
**74**	0.022058	0.050392	**210**	0.013099	0.039635			
**75**	0.022058	0.050392	**211**	0.013099	0.038884			
**76**	0.022058	0.050392	**212**	0.013099	0.038884			
**77**	0.01927	0.050392	**213**	0.013099	0.038884			
**78**	0.01927	0.050392	**214**	0.013099	0.038884			
**79**	0.01927	0.050392	**215**	0.013099	0.038884			
**80**	0.01927	0.050392	**216**	0.013099	0.038884			
**81**	0.01927	0.050392	**217**	0.013099	0.038884			
**82**	0.01927	0.050392	**218**	0.013099	0.038884			
**83**	0.01927	0.050392	**219**	0.013099	0.038884			
**84**	0.01927	0.050392	**220**	0.013099	0.038884			
**85**	0.01927	0.050392	**221**	0.013099	0.038884			
**86**	0.01927	0.050392	**222**	0.013099	0.038884			
**87**	0.01927	0.050392	**223**	0.013099	0.038884			
**88**	0.01927	0.050392	**224**	0.013099	0.038884			
**89**	0.01927	0.050392	**225**	0.013099	0.038884			
**90**	0.01927	0.050392	**226**	0.013099	0.038884			

**91**	0.01927	0.050392	**227**	0.013099	0.038884			
**92**	0.01927	0.050392	**228**	0.013099	0.038884			
**93**	0.01819	0.045535	**229**	0.013099	0.038884			
**94**	0.01819	0.045535	**230**	0.012481	0.038884			
**95**	0.01819	0.045535	**231**	0.012481	0.038884			
**96**	0.01819	0.045535	**232**	0.012481	0.038884			
**97**	0.018087	0.045535	**233**	0.012481	0.038884			
**98**	0.018087	0.045535	**234**	0.012481	0.038884			
**99**	0.018087	0.045535	**235**	0.012481	0.038884			
**100**	0.018087	0.045535	**236**	0.012481	0.037968			
**101**	0.018087	0.045535	**237**	0.012481	0.037968			
**102**	0.018087	0.045535	**238**	0.012481	0.037968			
**103**	0.018087	0.045535	**239**	0.012481	0.037968			
**104**	0.018087	0.045535	**240**	0.012481	0.037968			
**105**	0.018087	0.045535	**241**	0.012481	0.037968			
**106**	0.018087	0.045535	**242**	0.009916	0.037968			
**107**	0.018087	0.045535	**243**	0.009916	0.037968			
**108**	0.018087	0.045535	**244**	0.009916	0.037968			
**109**	0.018087	0.045535	**245**	0.009916	0.037968			
**110**	0.018087	0.045535	**246**	0.009916	0.037968			
**111**	0.018087	0.045535	**247**	0.009916	0.037968			
**112**	0.018087	0.045535	**248**	0.009916	0.037968			
**113**	0.018087	0.045535	**249**	0.009916	0.037968			
**114**	0.018087	0.045535	**250**	0.009916	0.037968			
**115**	0.018087	0.045535	**251**	0.009916	0.037928			
**116**	0.018087	0.045535	**252**	0.009916	0.037928			
**117**	0.018087	0.045535	**253**	0.009916	0.037812			
**118**	0.018087	0.045535	**254**	0.009916	0.037812			
**119**	0.018087	0.045535	**255**	0.009916	0.037812			
**120**	0.018087	0.045535	**256**	0.009795	0.037812			
**121**	0.018087	0.045535	**257**	0.009795	0.037812			
**122**	0.018087	0.045535	**258**	0.009795	0.037499			
**123**	0.018087	0.045535	**259**	0.009751	0.037499			
**124**	0.018087	0.045213	**260**	0.009751	0.037499			
**125**	0.018087	0.045213	**261**	0.009751	0.037499			
**126**	0.018087	0.045213	**262**	0.009751	0.037119			
**127**	0.018087	0.045213	**263**	0.009751	0.037119			
**128**	0.018087	0.045213	**264**	0.009751	0.037119			
**129**	0.018087	0.044328	**265**	0.009751	0.037119			
**130**	0.018087	0.044328	**266**	0.009366	0.037119			
**131**	0.018087	0.044328	**267**	0.009366	0.037119			
**132**	0.018087	0.044328	**268**	0.009366	0.037119			
**133**	0.018087	0.044328	**269**	0.009366	0.037119			
**134**	0.018087	0.041637	**270**	0.009366	0.037119			
**135**	0.018087	0.041637	**271**	0.009141	0.037119			
**136**	0.018087	0.041637	**272**	0.009141	0.037119			
